# Remarkably coherent population structure for a dominant Antarctic *Chlorobium* species

**DOI:** 10.1186/s40168-021-01173-z

**Published:** 2021-11-26

**Authors:** Pratibha Panwar, Michelle A. Allen, Timothy J. Williams, Sabrina Haque, Sarah Brazendale, Alyce M. Hancock, David Paez-Espino, Ricardo Cavicchioli

**Affiliations:** 1 School of Biotechnology and Biomolecular Sciences, UNSW Sydney, Sydney, New South Wales 2052 Australia; 2 Present address: Department of Molecular Sciences, Macquarie University, Sydney, New South Wales 2109 Australia; 3 Present address: Pegarah, Australia; 4 Present address: Institute for Marine and Antarctic Studies, University of Tasmania, 20 Castray Esplanade, Battery Point, Tasmania Australia; 5 Department of Energy Joint Genome Institute, Berkeley, CA USA; 6 Present address: Mammoth Biosciences, Inc., 1000 Marina Blvd. Suite 600, Brisbane, CA USA

**Keywords:** Antarctic microbiology, Green sulphur bacteria, Chlorobi, Vitamin B12, Metagenome-assembled genomes, Phylotype, Ecotype, Population structure, Host-virus interactions, Generalist virus, Meromictic lake, Microbial food web

## Abstract

**Background:**

In Antarctica, summer sunlight enables phototrophic microorganisms to drive primary production, thereby “feeding” ecosystems to enable their persistence through the long, dark winter months. In Ace Lake, a stratified marine-derived system in the Vestfold Hills of East Antarctica, a *Chlorobium* species of green sulphur bacteria (GSB) is the dominant phototroph, although its seasonal abundance changes more than 100-fold. Here, we analysed 413 Gb of Antarctic metagenome data including 59 *Chlorobium* metagenome-assembled genomes (MAGs) from Ace Lake and nearby stratified marine basins to determine how genome variation and population structure across a 7-year period impacted ecosystem function.

**Results:**

A single species, *Candidatus* Chlorobium antarcticum (most similar to *Chlorobium phaeovibrioides* DSM265) prevails in all three aquatic systems and harbours very little genomic variation (≥ 99% average nucleotide identity). A notable feature of variation that did exist related to the genomic capacity to biosynthesize cobalamin. The abundance of phylotypes with this capacity changed seasonally ~ 2-fold, consistent with the population balancing the value of a bolstered photosynthetic capacity in summer against an energetic cost in winter. The very high GSB concentration (> 10^8^ cells ml^−1^ in Ace Lake) and seasonal cycle of cell lysis likely make *Ca.* Chlorobium antarcticum a major provider of cobalamin to the food web. Analysis of *Ca.* Chlorobium antarcticum viruses revealed the species to be infected by generalist (rather than specialist) viruses with a broad host range (e.g., infecting Gammaproteobacteria) that were present in diverse Antarctic lakes. The marked seasonal decrease in *Ca.* Chlorobium antarcticum abundance may restrict specialist viruses from establishing effective lifecycles, whereas generalist viruses may augment their proliferation using other hosts.

**Conclusion:**

The factors shaping Antarctic microbial communities are gradually being defined. In addition to the cold, the annual variation in sunlight hours dictates which phototrophic species can grow and the extent to which they contribute to ecosystem processes. The *Chlorobium* population studied was inferred to provide cobalamin, in addition to carbon, nitrogen, hydrogen, and sulphur cycling, as critical ecosystem services. The specific Antarctic environmental factors and major ecosystem benefits afforded by this GSB likely explain why such a coherent population structure has developed in this *Chlorobium* species.

**Video abstract**

**Supplementary Information:**

The online version contains supplementary material available at 10.1186/s40168-021-01173-z.

## Background

The Chlorobiaceae family, including the *Chlorobium* genus, are green sulphur bacteria (GSB) that fix CO_2_ anaerobically using the reverse tricarboxylic acid cycle by performing anoxygenic photosynthesis using sulphide or other reduced sulphur compounds as electron donors [1–3]. GSB can perform primary production under conditions of low photosynthetically active radiation as they have very sensitive and efficient light-harvesting antennae in their photosynthetic apparatus [4–6]. Members of the *Chlorobium* genus have global representation, making important contributions to thermally diverse ecosystems, typically residing at the oxic-anoxic interface of the water column in stratified aquatic systems, and within benthic mats [7–19]. Their growth requirements, physiology, and ecology have been well studied [3, 5, 7–9, 11, 16, 18, 20–23], including the use of comparative genomics [24, 25] and metagenomics [13, 14, 19, 26] to study their roles in environmental communities.

**Fig. 1:**
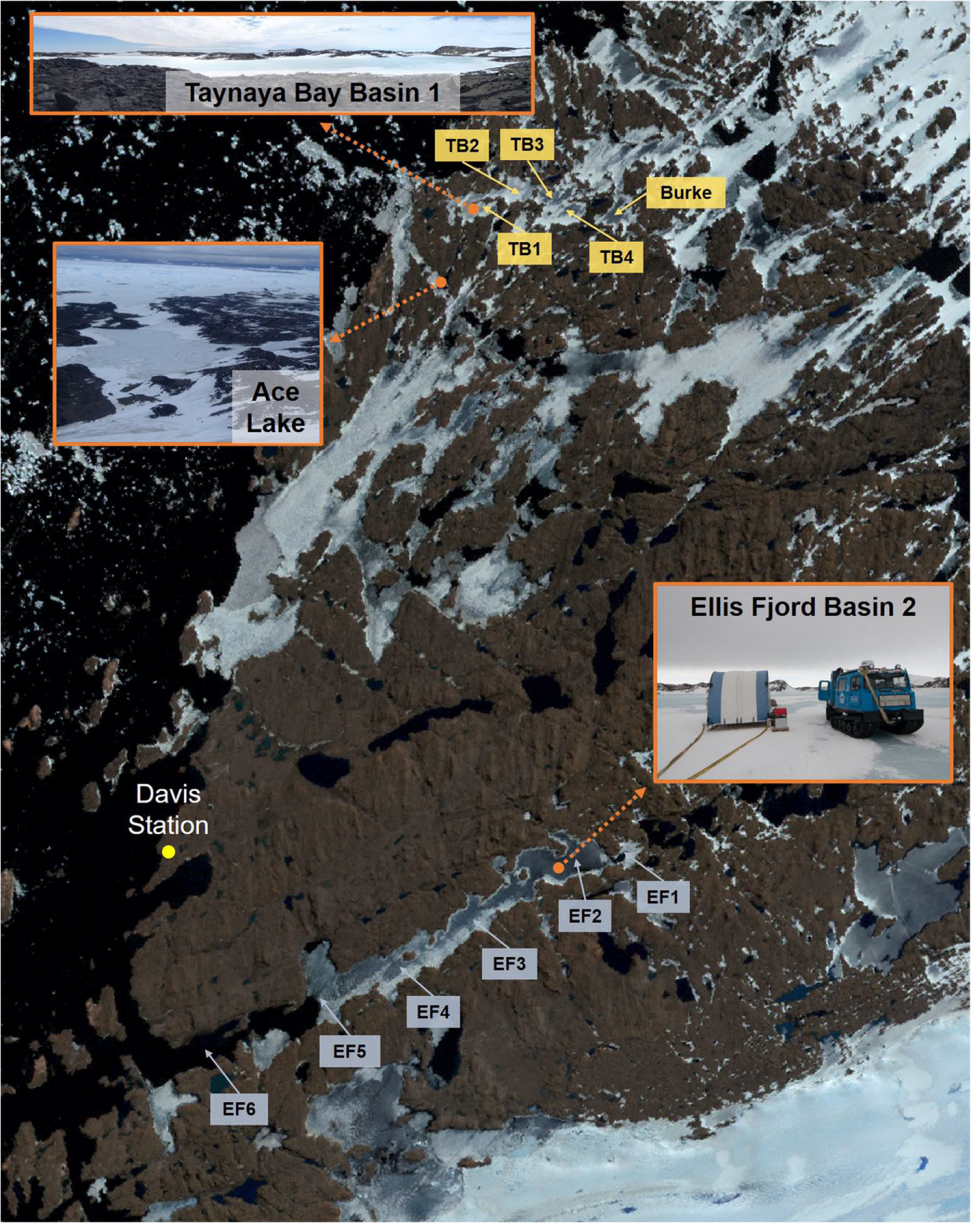
Location of Ace Lake, Ellis Fjord, and Taynaya Bay in the Vestfold Hills, East Antarctica. Ace Lake (68° 28′ S, 78° 11′ E) is 25 m deep with a strong halocline and chemocline that coincides with the oxic-anoxic interface at a depth of 12–15m, and supports the growth of a microbial community that was derived from the Southern Ocean about 5,000 years ago [19, 27, 29, 30]. Ellis Fjord (68° 36′ S, 78° 07′ E) is an ~ 10-km-long, narrow water inlet that contains six basins (EF1–EF6) that are up to 117 m deep, with the two inner basins (EF1 and EF2) being meromictic [31]. The sill at the entrance to Ellis Fjord is 4 m deep and the six marine basins are separated by sills of different depths (1–30 m) [8, 29, 31, 32]. Taynaya Bay (68° 27′ S, 78° 17′ E) is a marine water inlet with a maximum depth of up to 80 m, containing six basins, of which five (Burke and TB1–TB4) are meromictic [29, 31]. Ace Lake and Taynaya Bay Basin 1 are ~ 2 km apart, and Ellis Fjord Basin 2 is ~ 14 km to the west of Ace Lake. All three systems are covered by ice for much of the year. The satellite map of the Vestfold Hills and the distance measurements were produced using the interactive atlas available on Landsat Image Mosaic of Antarctica website [33]. The locations of Ellis Fjord and Taynaya Bay basins were from published data [29, 31]. The photos of the aquatic systems were taken by Sarah Brazendale and Rick Cavicchioli

In Antarctica, summer sunlight can persist for 24 hours and deliver intense photosynthetically active radiation to drive primary production by phototrophic communities of phytoplankton; levels as high as 1225 μE m^−2^ S^−1^ have been recorded [27, 28]. While photosynthetic algae are known to play key phototrophic roles in the Southern Ocean, as do cyanobacteria in continental aquatic systems, comparatively little is known about Antarctic GSB [7, 9, 19, 27]. The most well-characterized Antarctic GSB are *Chlorobium* from Ace Lake [13, 14, 19]. Ace Lake is one of many meromictic (stratified) lakes within East Antarctica, Vestfold Hills [29], a region that harbours Chlorobiaceae [8, 9] (Fig. 1). Using microscopy, growth and isolation approaches, Chlorobiaceae were identified in a number of lakes and fjords, including Ellis Fjord and Taynaya Bay [8, 9] (Fig. 1). Ellis Fjord and Taynaya Bay contain marine basins where shallow sills restrict water flow from the Southern Ocean thereby permitting stratification of the water column and the development of stable oxic-anoxic interfaces [29, 31, 32]. While Ace Lake is one of the most extensively studied systems in Antarctica in terms of microbiology [19, 30, 34], Ellis Fjord [8, 35, 36] and Taynaya Bay [8, 37] have had little study and no metagenomic assessments.

In Ace Lake, the *Chlorobium* abundance exhibits marked seasonal variation, with highest abundance in summer, numbers falling during winter, and lowest abundance in early spring (> 100-fold lower than summer) before a rebound back into summer [19]. The seasonal fluctuation was attributed primarily to changes in light hours rather than to the possible controlling effects of viral predation [19]. Despite the availability of very large metagenome datasets and associated metagenome-assembled genomes (MAGs) for *Chlorobium* from Ace Lake, genomic variation and population structure have not been examined.

Insight into Antarctic haloarchaea genomic variation has been gained from analyses of single nucleotide polymorphisms (SNPs), and low coverage regions (LCRs) generated from mapping metagenome reads to reference genomes or MAGs [38–41]. LCRs arise from phylotypes that do not possess the sequences or have sufficiently diverged sequences that do not recruit. Phylotypes that contain genes in LCRs possess a unique genomic capacity compared to phylotypes that lack the genes. Highly divergent genes within LCRs can also confer distinct functional traits by conferring altered protein functions such as specificity for substrates or substrate preference, altered specificity for viral attachment to cell surface proteins, and so forth. Examining the function of genes from variable regions can determine whether phylotypes represent ecotypes that may occupy distinct ecological niches within an ecosystem.

In this study, the MAGs of *Chlorobium* from Ace Lake, Ellis Fjord, and Taynaya Bay were compared to each other and to non-Antarctic *Chlorobium* species in order to determine the following: (i) which *Chlorobium* species characterize the individual Antarctic systems; (ii) whether the species are endemic to Antarctica; (iii) what genomic traits characterize phylotypes within and between the Antarctic systems, including seasonal populations in Ace Lake. *Chlorobium* phylotypes and *Chlorobium* viruses were examined to determine: (i) the biogeographic distribution of *Chlorobium* viruses in the Vestfold Hills; (ii) the types of viral defence systems possessed by the *Chlorobium*; (iii) the characteristics of virus-host dynamics in each system. As a result, we greatly expanded knowledge of Antarctic Chlorobiaceae and learned how the unique Antarctic environment controls the evolution of these primary producers.

## Results and discussion

### Overview of metagenomes and *Chlorobium* MAGs

Ace Lake, Ellis Fjord, and Taynaya Bay are herein referred to as AL, EF, and TB, respectively. Biomass was collected by filtration through a 20-μm pre-filter onto large format filters (3, 0.8, and 0.1 μm) for AL and EF, and into Sterivex cartridges (0.22 μm) for TB (see the “Methods” section). The filtered reads from 18 AL (~ 99 Gb), three EF (~48 Gb) and one TB (~ 12 Gb) oxic-anoxic interface metagenomes were used for fragment recruitment (FR) analyses (Additional file 1: Table S1); for these analyses, the AL and EF metagenome reads from the three filter fractions representing a single sample (date and depth) were pooled to form merged metagenomes (see the “Methods” section). The assembled contigs from individual AL (~ 6 Gb), EF (~ 7 Gb) and TB (~ 700 Mb) metagenomes (Additional file 1: Table S1) were used to determine the *Chlorobium* OTU abundance distribution in the three Vestfold Hills systems, and for viral analyses.

A total of 59 high or medium quality MAGs were analysed, of which 31 AL, five EF, and two TB high-quality MAGs had ≥ 99% genome completeness (Additional file 1: Table S2; Additional file 2: Dataset S1). The MAGs represented 67,265 genes on 1124 *Chlorobium* contigs, and both 16S rRNA gene and FmoA (Fenna-Matthews-Olson protein; bacteriochlorophyll A) protein sequences were used as phylogenetic markers [42]. For FR analyses the AL_ref MAG (Dec 2014, 19 m depth, 0.1 μm-filter) contained 27 contigs and 1,797 genes and was 99% complete (1,812,610 bp), and the EF_ref MAG (Oct 2014, 45-m depth, 3-μm filter) contained 32 contigs and 1807 genes and was 99% complete (1,836,564 bp) (Additional file 1: Tables S2 and S3; Additional file 2: Dataset S1).

### *Chlorobium* species present in EF and TB

**Fig. 2:**
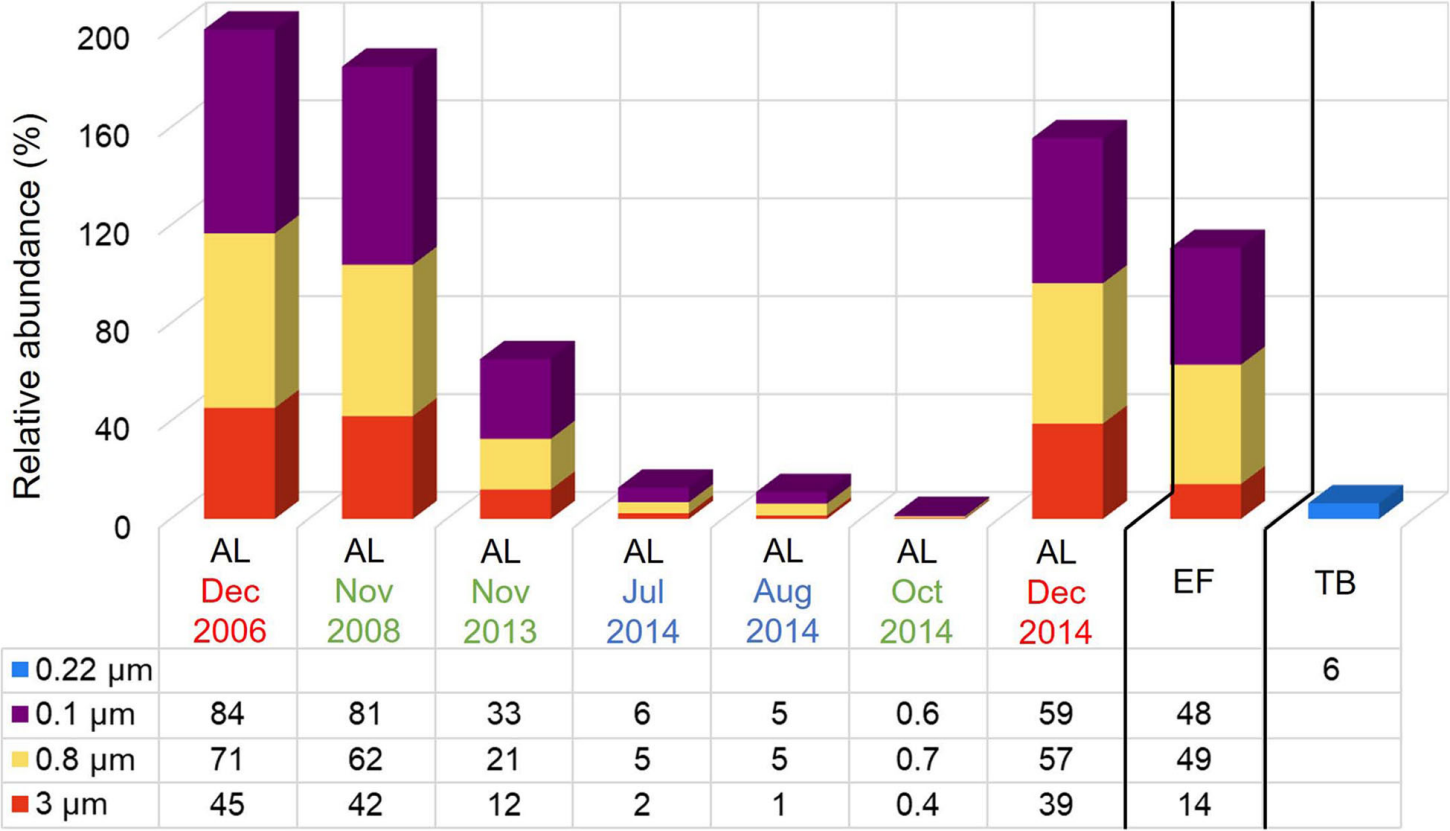
*Ca.* Chlorobium antarcticum abundance distribution in the Vestfold Hills. The stacked bar chart shows the relative abundance of *Ca.* Chlorobium antarcticum in the oxic-anoxic interface of Ace Lake (AL), Ellis Fjord (EF), and Taynaya Bay (TB). The AL abundances were generated from a time-series of metagenomes from different seasons (*x*-axis: Dec summer, red font; Jul and Aug winter, blue font; Oct and Nov spring, green font), whereas the EF and TB abundances were from metagenomes from spring (EF, Oct 2014; TB, Nov 2014) (Additional file 1: Table S1). The AL and EF data were from samples collected on large format filters (*y*-axis: 3 μm, red; 0.8 μm, yellow; 0.1 μm, purple), whereas the TB data were from samples collected using Sterivex cartridges (*y*-axis: 0.22 μm, blue). Due to the dynamic range of the data (0.4–84%), the percentage abundance values for *Ca.* Chlorobium antarcticum in metagenomes from each filter fraction and time period (see relative abundance calculation in the “Methods” section) are shown below the bar chart. Filter fractions: 0.22, 0.22–20 μm; 0.1, 0.1–0.8 μm; 0.8, 0.8–3 μm; 3, 3–20 μm

*Chlorobium* OTUs were most abundant in EF (45 m) and TB (11 m) at depths where oxic-anoxic interfaces have previously been recorded [8, 29, 31], with a relative abundance (EF, ≤ 49%; TB, 6%) comparable to the range of abundances observed in AL (< 1–84%; Fig. 2) [19]. In TB where *Chlorobium* had lower relative abundance than EF or AL, the Simpson’s index of diversity was higher (1 − *λ*′ > 0.9 compared to ≤ 0.7).

All 16S rRNA genes from AL, EF, and TB *Chlorobium* MAGs had identical sequences (1505 bp), as did all FmoA protein sequences (366 aa) (Additional file 1: Fig. S1). The pair-wise, average nucleotide identify (ANI) of all *Chlorobium* MAGs was ≥ 99.9% over ≥ 92% alignment fraction. FR of AL, EF, and TB metagenome reads to the *Chlorobium* 16S rRNA gene (EF_ref MAG) revealed a number of SNPs with variant frequency ≥ 0.01 (i.e., at least 1% of the aligned reads contained the SNP) (Additional file 3: Dataset S2). All of these SNPs, except one from the AL Dec 2014 merged metagenome, two from the EF merged metagenome, and four from the TB metagenome, had very low read depth (on average < 5) and could represent sequencing errors (Additional file 3: Dataset S2). In contrast, the read depth of the *Chlorobium* 16S rRNA gene sequence (lacking SNPs) was > 80 in all AL (except Oct 2014, read depth 31), EF and TB metagenomes, and > 11,000 in some metagenomes (Additional file 3: Dataset S2). These data indicate that the same species of *Chlorobium* was present in all three Vestfold Hills systems, representing at least 97% of AL, 97% of EF, and 98% of TB *Chlorobium* population, and was the only detectable *Chlorobium* species in AL throughout a seasonal cycle (also see below in “*Ca.* Chlorobium antarcticum population variation between AL, EF, and TB”).

**Fig. 3:**
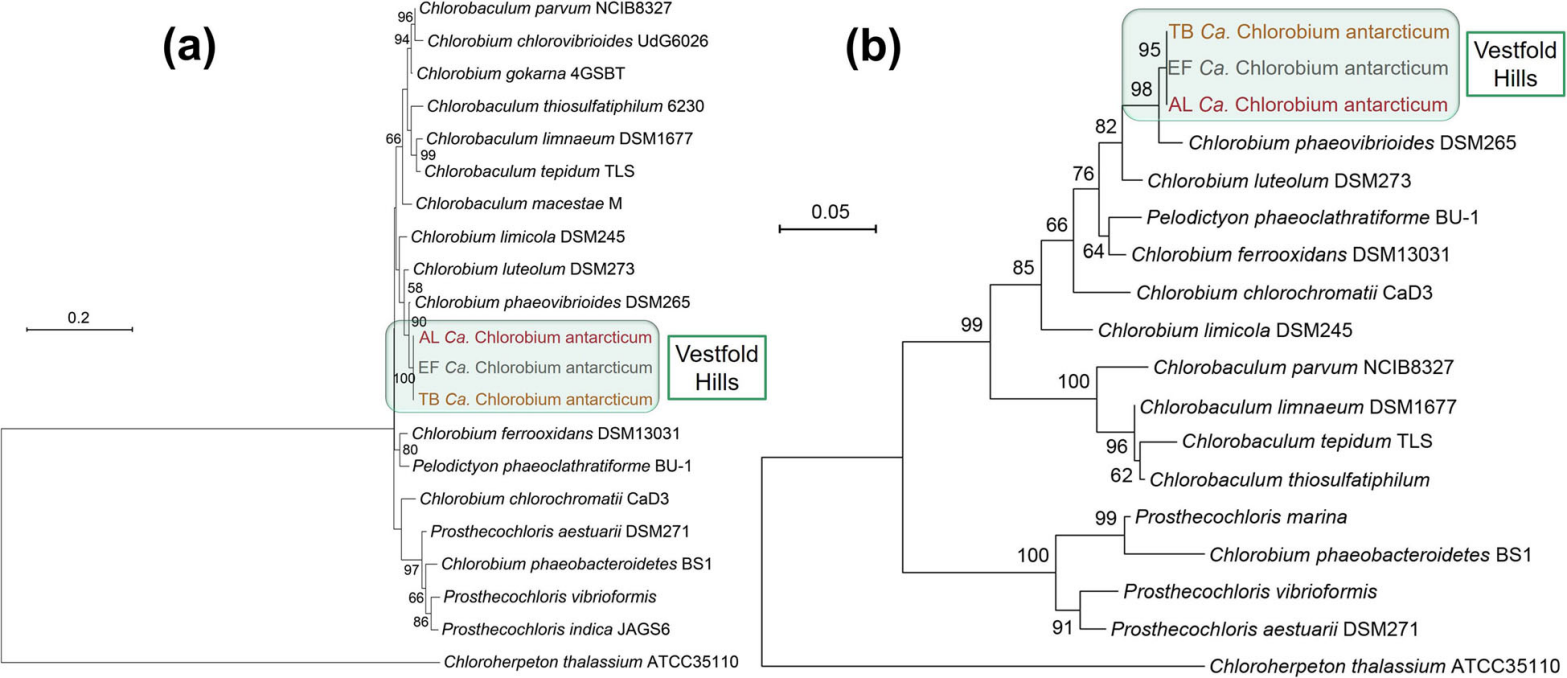
Phylogeny of *Ca.* Chlorobium antarcticum from the Vestfold Hills. Maximum-likelihood trees for **a** 16S rRNA gene and **b** FmoA protein sequences of members of the Chlorobiaceae family. The phylogenetic trees were prepared with MEGA X v10.1.7 using 1,000 bootstrap values. Trees are drawn to scale, and branch-length scale bars are provided in each panel. Numbers next to branches represent bootstrap values showing the percentage of trees in which the taxa clustered together. Only bootstrap values greater than 50% are shown. *Ca.* Chlorobium antarcticum from the Vestfold Hills systems, AL, EF, and TB, are highlighted

IMG (Integrated Microbial Genomes) taxonomy denoted all MAGs as most closely related to *Chlorobium phaeovibrioides* DSM 265 (herein referred to as Cpv-DSM265). The 16S rRNA gene identity (99%; 17 nt mismatches; Additional file 1: Fig. S1a), FmoA protein identity (98%; six aa mutations; Additional file 1: Fig. S1b), ANI (85% over 80–86% alignment fraction), and average amino acid identity (AAI; 89%) distinguish the Antarctic species from Cpv-DSM265, and these differences are reflected in 16S rRNA gene and FmoA protein trees (Fig. 3) (also see below in “Comparison of *Ca.* Chlorobium antarcticum to Cpv-DSM265 and global representation”). In view of the genomic and phylogenetic differences we name the Antarctic species, *Candidatus* Chlorobium antarcticum sp. nov. (from ant.arc'ti.cum. L. neut. adj. *antarcticum* southern, Antarctic) (type MAG AL_ref MAG = 3300023061_2; 99% complete; 0.55% contamination) (Additional file 1: Table S2; Additional file 2: Dataset S1).

### *Ca.* Chlorobium antarcticum population variation within AL

**Fig. 4:**
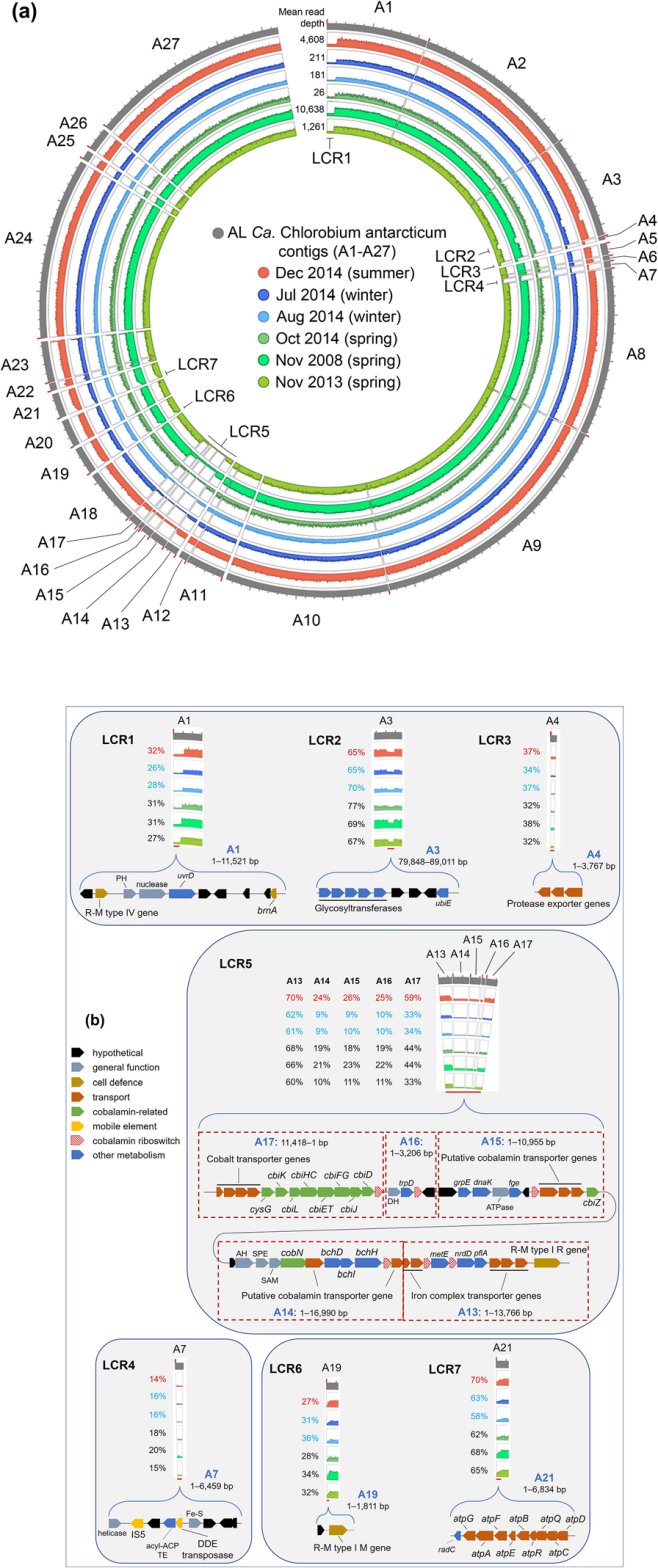
Genomic variation within the AL *Ca*. Chlorobium antarcticum population from different seasons. **a** Circos plot depicts read depth distribution of *Ca*. Chlorobium antarcticum in AL oxic-anoxic interface in summer (Dec), winter (Jul, Aug) and spring (Oct, Nov). The mean read depth of the AL_ref MAG in each merged metagenome (Additional file 1: Table S14) is shown at the beginning of each annulus (top). Outer to inner annuli and *y*-axis range: AL_ref MAG contigs A1–A27 ( ); Dec 2014 (, 0–10,000); Jul 2014 (, 0–800); Aug 2014 (, 0–800); Oct 2014 (, 0–100); Nov 2008 (, 0–20,000); Nov 2013 (, 0–3,000). The *x*-axis is shown on the outermost annulus: beginning of each contig, red tick; 10-kb length, grey tick; LCRs, labelled 1–7. Scaffolds (separated by large gaps) were represented by contigs (separated by small gaps): A1–4, A5–10, A11–12, A13–18, A19, A20, A21, A22–23, A24, A25, A26 and A27 (see MAG contigs ordering in the “Methods” section; Additional file 1: Table S3). **b** The relative coverages and gene composition of LCR1–7. The percentages indicate the proportion of the *Ca*. Chlorobium antarcticum population from each merged metagenome, including summer (red font) and winter (blue font) populations that contain the genes in the LCR. In LCR5, the arrangement of contigs A13–A17 was determined through sequence comparisons with other AL, EF and TB *Ca*. Chlorobium antarcticum MAGs (see MAG contigs ordering in the “Methods” section). Genes: acyl-ACP TE, acyl-acyl carrier protein thioesterase; *atpA*, ATP synthase subunit alpha; *atpB*, ATP synthase subunit a; *atpC*, ATP synthase subunit epsilon; *atpD*, ATP synthase subunit beta; *atpE*, ATP synthase subunit c; *atpF*, ATP synthase subunit b; *atpG*, ATP synthase subunit gamma; *atpQ*, ATP synthase N subunit Q; *atpR*, ATP synthase N subunit R; *bchD*, magnesium chelatase subunit D; *bchH*, magnesium chelatase subunit H; *bchI*, magnesium chelatase subunit I; *brnA*, antitoxin component of BrnTA type II T-A system; *cbiD*, cobalt-precorrin-5B C(1)-methyltransferase; *cbiET*, cobalamin biosynthesis bifunctional protein CbiET; *cbiFG*, cobalt-precorrin-4 C(11)-methyltransferase/cobalt-precorrin-5A hydrolase; *cbiHC*, cobalamin biosynthesis protein CbiHC; *cbiJ*, cobalt-precorrin-6A reductase; *cbiK*, sirohydrochlorin cobaltochelatase; *cbiL*, cobalt-precorrin-2 C(20)-methyltransferase; *cbiZ*, adenosylcobinamide amidohydrolase; *cobN*, cobaltochelatase subunit N; *cysG*, uroporphyrinogen-III C-methyltransferase; *dnaK*, molecular chaperone DnaK; *fge*, formylglycine-generating enzyme required for sulphatase activity; *grpE*, molecular chaperone GrpE; IS5, IS5 family transposase; *metE*, 5-methyltetrahydropteroyltriglutamate-homocysteine methyltransferase; *nrdD*, ribonucleoside-triphosphate reductase; *pflA*, pyruvate formate lyase activating enzyme; *radC*, DNA repair protein RadC; *trpD*, anthranilate phosphoribosyltransferase; *ubiE*, ubiquinone/menaquinone biosynthesis C-methylase UbiE; *uvrD*, DNA helicase UvrD. General function genes: AH, amidohydrolase; ATPase, AAA domain-containing ATPase; DH, dehydrogenase; Fe-S, ferredoxin domain-containing protein; helicase, superfamily I DNA and/or RNA helicase; nuclease, PD-(D/E)XK nuclease superfamily protein; PH, Pleckstrin Homology domain-containing protein; SAM, radical S-adenosyl-l-methionine superfamily protein; SPE, sugar phosphate epimerase

Aligning AL metagenome filtered-reads to the AL_ref MAG to identify SNPs determined that no fixed mutations (variant frequency ≥ 0.9) were present. However, seven LCRs were identified (Fig. 4; Additional file 1: Table S4). The LCRs encoded cell wall modification, cell defence, transport, DNA repair, protein modification, metabolism, mobile element, and hypothetical genes (Additional file 1: Tables S4, S5, and S6). Metabolic genes included: (i) a cluster of nine genes representing the N-type rotary ATPase (N-ATPase) operon (*atpD*, *atpC*, *atpQ*, *atpR*, *atpB*, *atpE*, *atpF*, *atpA*, *atpG*), which codes for ATPase subunits involved in ATP-dependent efflux of Na^+^ or H^+^ ions; (ii) a cluster of eight single-copy genes involved in the anaerobic pathway for cobalamin biosynthesis (*cbiD*, *cbiJ*, *cbiL*, *cbiK*, *cysG*, and bifunctional *cbiFG*, *cbiET*, *cbiHC*), plus a single copy gene involved in cobinamide salvaging (*cbiZ*); (iii) a gene cluster containing one cobaltochelatase (*cobN*) and three magnesium chelatase (*bchD*, *bchH*, *bchI*) genes; (iv) TonB-dependent and ABC transporter proteins involved in the import of iron, cobalt, and cobalamin across the outer membrane and inner membrane, respectively; (v) a gene cluster for export of proteases (Additional file 1: Tables S5 and S6).

A seasonal pattern was observed, with the proportion of the *Ca.* Chlorobium antarcticum population that possessed the genes within LCRs tending to be higher in summer than in winter or spring (Additional file 1: Tables S4, S5, and S6), most notably for genes associated with cobalamin synthesis and transport (also see below in “Population structure of cobalamin biosynthesis and transport genes”).

The range of transport genes present in the LCRs of *Ca.* Chlorobium antarcticum MAGs is indicative of the population supporting a diversity of transport abilities (Additional file 1: Table S4 and S5). For example, protease export systems with similarity to *Pseudomonas aeruginosa* AprDEF were present in at least 28% of the *Ca.* Chlorobium antarcticum population, and abundance did not vary with season (Group 7 in Additional file 1: Table S5). For GSB, iron is an essential trace element required for the photosynthetic reaction centre [16]. The concentration of iron in AL increases with depth, being ~ 1 μM at the oxic-anoxic interface [30, 43]. TonB-dependent transporter and ABC transporter genes enable the uptake of both inorganic iron and organic forms of iron (siderophores, hemoproteins) [44]. All *Ca.* Chlorobium antarcticum MAGs contained two sets of ferrous iron transporter genes (*feoABC* and *feoAB*), and three TonB-dependent transporter genes potentially involved in iron complex import across the outer membrane. However, the ABC transporters associated with the uptake of iron complexes were only identified in LCRs (Groups 1 and 2 in Additional file 1: Table S5), indicating an augmented capacity for these phylotypes to source exogenous iron (at least 56% of the *Ca.* Chlorobium antarcticum population).

An N-ATPase operon (*atpDCQRBEFAG*) was present in at least 61% of the *Ca.* Chlorobium antarcticum population, with abundance varying only marginally by season (Group 8 in Additional file 1: Table S5); in addition, F_0_F_1_ ATP synthase genes were present throughout the *Ca.* Chlorobium antarcticum population. N-ATPases utilize ATP to actively transport Na^+^ or H^+^ ions out of the bacterial cell [45–47]. The *Ca.* Chlorobium antarcticum ATPase subunit c amino acid sequence included the two glutamate residues in both of its C- and N-terminal helices that are diagnostic of Na^+^-binding [45–47], indicating it functions in Na^+^ export. N-ATPase genes have been identified in some Chlorobi, including *Chlorobaculum parvum*, *Chlorobaculum tepidum* (partial locus only), *Pelodictyon luteolum*, and *Prosthecochloris aestuarii* [48, 49].

### *Ca.* Chlorobium antarcticum population variation between AL, EF, and TB

**Fig. 5:**
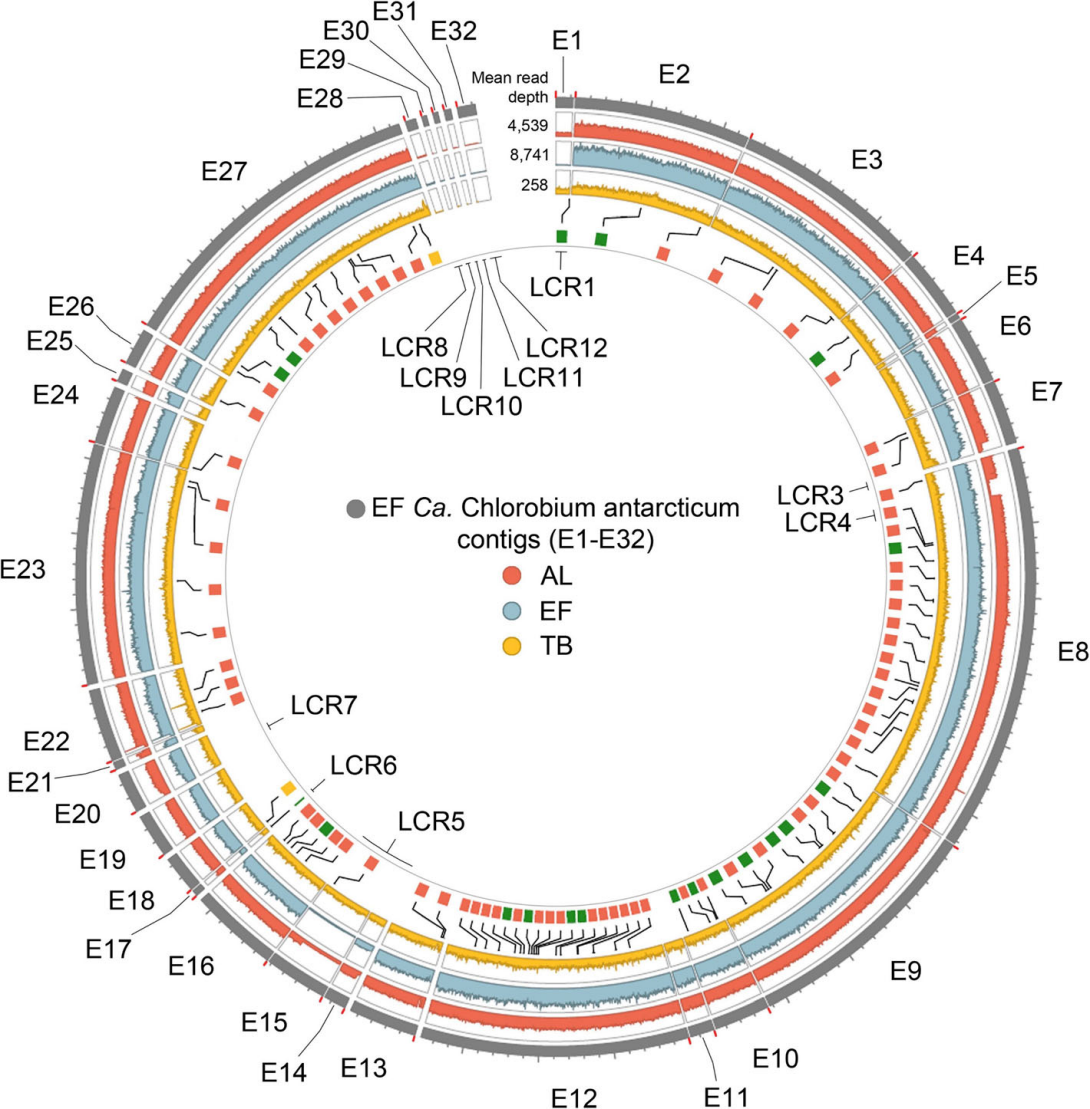
Genomic variation between *Ca*. Chlorobium antarcticum populations from AL, EF, and TB. Circos plot depicts read depth distribution of *Ca*. Chlorobium antarcticum in the oxic-anoxic interface of AL (Dec 2014), EF, and TB. The mean read depth of the EF_ref MAG in each merged metagenome (Additional file 1: Table S14) is shown at the beginning of each annulus (top). Outer to inner annuli and their *y*-axis range: EF_ref MAG contigs E1–E32 (); Dec 2014 (, 0–10,000); EF 45m ( , 0–15,000); TB 11m (, 0–800); EF_ref MAG genes containing SNPs in AL (), TB (), or both (), with connecting lines indicating gene position. The *x*-axis is shown on the outermost annulus: beginning of each contig, red tick; 10-kb length, grey tick; LCRs, labelled 1–12, with LCR1–7 as for AL_ref MAG (Fig. 4). Scaffolds were represented by contigs: E1–7, E8–12, E13, E14–16, E17–18, E19, E20, E21–22, E23–24, E25, E26, E27, E28, E29, E30, E31, and E32 (see MAG contigs ordering in the “Methods” section; Additional file 1: Table S3)

Similar to the analysis of SNPs within the AL population, no fixed SNPs were observed for EF metagenome reads against the EF_ref MAG. However, from 1807 genes in the EF_ref MAG, SNPs were identified in 68 genes only from AL, two only from TB, and 19 genes from both AL and TB (Fig. 5; Additional file 1: Table S7). Most SNPs occurred in genes involved in intracellular functions, with a smaller proportion in cell wall modification, substrate transport, and membrane protein genes. SNPs were present in regions of the EF_ref MAG that had even FR coverage, except for those in a hypothetical gene (contig E1, Additional file 1: Table S7), a precorrin-3B methylase/precorrin isomerase gene (contig E15, Additional file 1: Table S7), and gene for a receptor for the TonB-dependent uptake of iron-containing proteins (contig E17, Additional file 1: Table S7). This indicated that the AL and TB SNPs tended to occur within all *Ca.* Chlorobium antarcticum subpopulations, and were therefore characteristic of each system.

A total of 12 LCRs were identified from FR of AL, EF and TB metagenome reads to the EF_ref MAG (Fig. 5; Additional file 1: Table S4). Notably, five AL LCRs identified against the AL_ref MAG were also LCRs from FR of AL, EF, and TB reads to the EF_ref MAG (Additional file 1: Table S4) indicating that the main (detectable) *Ca.* Chlorobium antarcticum phylotypes existed in all three Vestfold Hills systems. The LCRs encoded cell wall modification, cell defence, transport, DNA repair, protein modification, Na^+^ or H^+^ ion efflux, anaerobic cobalamin biosynthesis, cobinamide salvaging, and cobalt/magnesium chelatase genes, similar to the gene functions of the AL_ref MAG LCRs. LCRs specific to the EF_ref MAG included cell wall modification, general function, and hypothetical genes.

To assess gene order of phylotypes, the contigs of AL, EF, and TB MAGs were aligned to AL_ref MAG (Additional file 3: Dataset S2). Most of the AL_ref MAG contigs that did not align to the contigs of the other MAGs were from AL_ref MAG LCRs, consistent with gene order varying in *Ca.* Chlorobium antarcticum phylotypes.

**Fig. 6:**
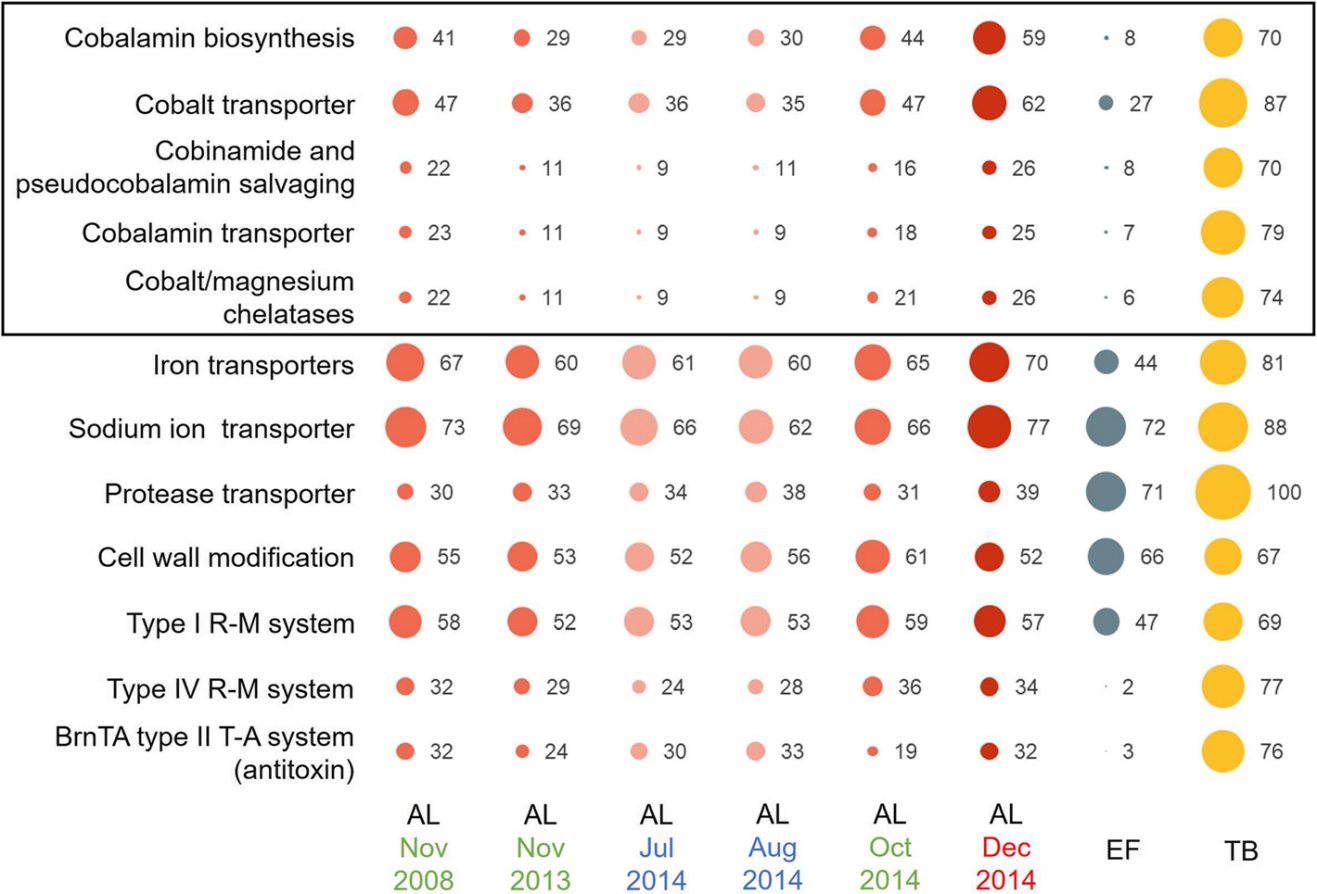
Abundance and function of genes in *Ca.* Chlorobium antarcticum LCRs. The scatter plot shows relative coverages of genes associated with transport, metabolism, cell wall modification, and cell defence that were identified in LCRs of *Ca.* Chlorobium antarcticum from AL (, , ), EF () or TB (). The AL or EF oxic-anoxic interface merged metagenomes and TB oxic-anoxic interface metagenome are listed (*x*-axis). Gene functions associated with *Ca.* Chlorobium antarcticum LCRs are listed (*y*-axis). AL data represent a time-series: summer, red font (); winter, blue font (); spring, green font (). Bubble diameter scales with relative coverage expressed as a percentage (enumerated to the right of each bubble). The percentages indicate the proportion of the *Ca.* Chlorobium antarcticum population that contains the LCR genes, where 100% (e.g., the TB protease transporter genes) indicates all *Ca.* Chlorobium antarcticum MAGs contain the genes. Genes: cobalamin biosynthesis — cobalt-precorrin-5B C(1)-methyltransferase CbiD, cobalt-precorrin-6A reductase CbiJ, cobalt-precorrin-4 C(11)-methyltransferase/cobalt-precorrin-5A hydrolase CbiFG, cobalamin biosynthesis bifunctional protein CbiET, cobalamin biosynthesis protein CbiHC, cobalt-precorrin-2 C(20)-methyltransferase CbiL, sirohydrochlorin cobaltochelatase CbiK, uroporphyrinogen-III C-methyltransferase CysG; cobalt transporter — cobalt/nickel transport system proteins CbiO, CbiQ, CbiN; cobinamide and pseudocobalamin salvaging — adenosylcobinamide amidohydrolase; cobalamin transporter — TonB-dependent receptor protein, cobalamin transporter BtuB, iron/cobalamin transport system ATP-binding protein, cobalamin import system permease protein BtuC, iron/cobalamin transport system substrate-binding protein; cobalt/magnesium chelatases — magnesium chelatase subunits BchH, BchI, BchD, and cobaltochelatase subunit CobN; Iron transporters — TonB-dependent receptor protein, two iron complex transport system substrate-binding proteins, iron complex transport system permease protein, iron complex transport system ATP-binding protein, TonB-dependent haem/haemoglobin receptor family protein; Sodium ion transporter — N-ATPase operon subunits AtpG, AtpA, AtpF, AtpE, AtpB, AtpR, AtpQ, AtpC, and AtpD; Protease transporter — two ATP-binding cassette subfamily C exporters for protease/lipase, protease secretion system membrane fusion protein, protease secretion system outer membrane protein; cell wall modification — phosphatidylinositol alpha-1,6-mannosyltransferase, five glycosyltransferase involved in cell wall biosynthesis, glycosyltransferase family 4 protein, UDP-N-acetyl-d-mannosaminuronic acid dehydrogenase; type I R-M system — type I restriction enzyme subunits R and M; type IV R-M system — type IV restriction enzyme; BrnTA type II T-A system (antitoxin) — BrnA antitoxin. R-M, restriction-modification; T-A, toxin-antitoxin

While the main phylotypes were shared amongst systems, some LCRs (e.g., contigs E29–E32) had very low read depth (≤ 2%) in all three systems (Additional file 1: Table S4) indicating that the genetic capacity represented by these contigs was rare within the overall *Ca.* Chlorobium antarcticum population. The relative coverage of some LCRs also varied considerably between systems indicative of different population structures for these specific genes (Fig. 6; Additional file 1: Table S4). For example, the 11-kb contig E1 represented 3% of the EF *Ca.* Chlorobium antarcticum population but 69% of the TB *Ca.* Chlorobium antarcticum population. Based on relative coverage, phylotypes represented by LCRs contributed more to the TB *Ca.* Chlorobium antarcticum population than to the AL or EF populations (Fig. 6; Additional file 1: Table S4). However, EF_ref MAG SNPs were more prevalent for AL than TB, indicating that SNP-based variation was more similar between EF and TB *Ca.* Chlorobium antarcticum populations than either were to the AL population. The apparent differences in contribution of LCRs and SNPs to the *Ca.* Chlorobium antarcticum population from each system may reflect the cellular mechanisms involved in generating variation (e.g., DNA repair) and/or environmental effects (e.g., selective forces), and determining the causes will require further investigation (also see Additional file 1: Supplementary text).

To determine if phylotypes from AL, EF, or TB existed with greater sequence divergence than the FR matching criteria permitted (≥ 95% identity), G + C content of metagenome contigs was plotted against read depth and the taxonomy of contig clusters assigned (Additional file 1: Fig. S2); this approach was previously used to identify phylotypes of Antarctic haloarchaea with significantly different genomes to known species [38]. The contigs in the main cluster were from *Ca.* Chlorobium antarcticum (Additional file 1: Fig. S2). Aside from a number of contigs from some smaller clusters (see the “Methods” section), none of the OTUs of small clusters represented *Ca.* Chlorobium antarcticum, indicating that phylotypes with more divergence than the cutoffs used for assigning LCRs were not detectable in the metagenome data.

Collectively, the high ANI/AAI between MAGs (see above in “*Chlorobium* species present in EF and TB”), the small extent of variation represented by SNPs and LCRs, and the taxonomic findings of the analysis of GC/read-depth clusters, illustrate that the *Ca.* Chlorobium antarcticum population has remarkably little genomic variation.

### Comparison of *Ca.* Chlorobium antarcticum to Cpv-DSM265 and global representation

**Fig. 7:**
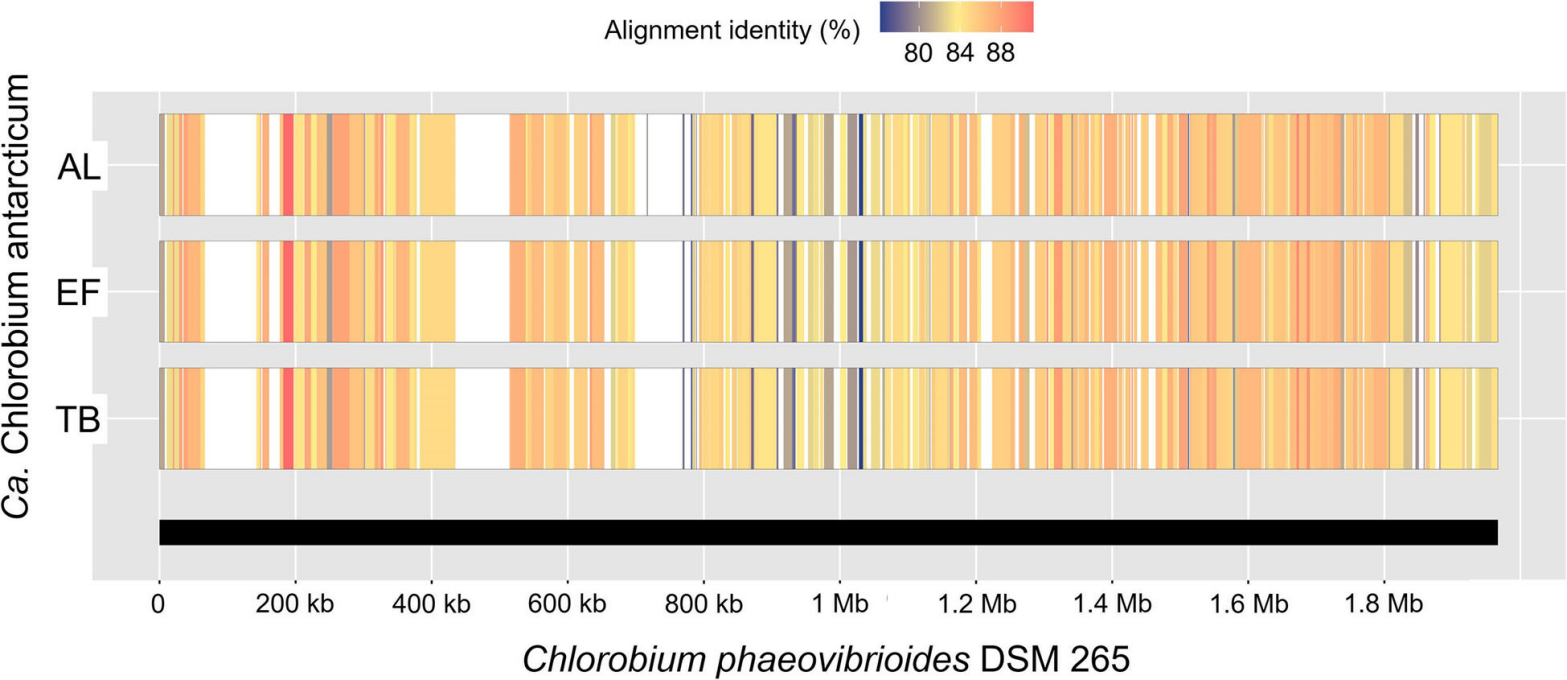
Alignment showing nucleotide identity between *Ca*. Chlorobium antarcticum MAGs and the Cpv-DSM265 genome**.** AL, AL_ref MAG; EF, EF_ref MAG; TB, MAG from 11 m depth. The Cpv-DSM265 genome (thick black line) is the reference, with *x*-axis labels denoting genome base pair positions. The alignment gaps (white regions) denote no match between the reference sequence and MAG contigs (MAG contigs that had no matches to the reference genome are not shown). The gradient bar denotes percentage nucleotide identity from 76% (blue) to 92% (red)

The AL, EF, and TB contigs had overall low nucleotide identity (< 90%) when aligned to the Cpv-DSM265 genome, with many gaps and differences in gene content (Fig. 7). As described previously, *Ca.* Chlorobium antarcticum is green rather than brown in colour (unlike Cpv-DSM265); as well as possessing the biosynthetic pathway for chlorobactene (found in green-coloured GSB), *Ca.* Chlorobium antarcticum lacks the capacities to synthesize bacteriochlorophyll *e* and isorenieratene, both found in Cpv-DSM265 and other brown-coloured GSB [19].

Many of the Cpv-DSM265 genes that caused the alignment gaps were associated with transposases and hypothetical genes (Additional file 3: Dataset S2). However, some were genes involved in thiosulphate oxidation (*sox* gene cluster containing *soxA*, *soxB*, *soxX*, *soxY*, *soxZ*), assimilatory sulphate reduction (*cysC*, *cysD*, *cysN*), and pilus assembly, none of which were present in the *Ca.* Chlorobium antarcticum MAGs. GSB do not tend to have a genomic capacity to perform assimilatory sulphate reduction [50], and it has been speculated that Cpv-DSM265 acquired the *sox* gene cluster on a mobile element from another member of the Chlorobiaceae family that originated in Proteobacteria [51]. *Ca.* Chlorobium antarcticum is therefore predicted to not be able to assimilate sulphate or to oxidise thiosulphate.

A number of *Ca.* Chlorobium antarcticum contigs did not align to the Cpv-DSM265 genome (Additional file 3: Dataset S2). These contigs contained anaerobic cobalamin biosynthesis, cobalt transport, cobalamin transport, cobalt/magnesium chelatase, and N-ATPase genes, all of which were absent from the Cpv-DSM265 genome. While cobalamin transport and magnesium chelatase genes were present in all *Ca.* Chlorobium antarcticum MAGs, all of the contigs that did not align with the Cpv-DSM265 genome represented LCRs of the AL_ref MAG and EF_ref MAG (Additional file 1: Tables S4, S5, and S6). It is therefore possible that Cpv-DSM265 represents a phylotype that lacks these genetic loci, or that the loci represent functions that are of particular importance to the Antarctic *Ca.* Chlorobium antarcticum population (also see below in “Population structure of cobalamin biosynthesis and transport genes”).

The *Ca.* Chlorobium antarcticum MAGs encoded multiple glycosyltransferase genes involved in cell wall biosynthesis that were not identified in the Cpv-DSM265 genome; the glycosyltransferases were represented throughout the *Ca.* Chlorobium antarcticum population, with only a few in LCRs (Additional file 1: Table S4), and are therefore characteristic of this Antarctic species. The glycosyltransferases may fulfil roles in cold adaptation through their function in biosynthesis and modification of cell walls [13, 52]. RNA helicases present in LCRs may also fulfil roles in cold adaptation through a potential functional capacity to unravel RNA secondary structures and influence rates of protein synthesis [53, 54]. The CRISPR-Cas defence systems [55] varied between the two *Chlorobium* species with *Ca.* Chlorobium antarcticum containing subtype I-E and Cpv-DSM265 containing subtype I-C (also see below in “*Ca.* Chlorobium antarcticum-virus interactions”). These genomic differences underscore specific metabolic and defence capabilities of the two *Chlorobium* species.

The global representation of *Ca.* Chlorobium antarcticum was assessed by matching the *Ca.* Chlorobium antarcticum 16S rRNA gene to all 16S rRNA genes from public metagenomes and genomes and the *Ca.* Chlorobium antarcticum FmoA protein sequence to all proteins from genomes (including MAGs and single-cell genomes) in IMG. All metagenome and genome matches were ≤ 99% 16S rRNA gene identity, and with the exception of Cpv-DSM265 (98% identity), all FmoA sequences had < 98% identity (Additional file 4: Dataset S3). The inability to identify *Ca.* Chlorobium antarcticum outside of Antarctica was in marked contrast to its representation in data from the three Vestfold Hills systems.

### Population structure of cobalamin biosynthesis and transport genes

Cobalamin and cobamide analogues are cofactors that function in a variety of metabolic processes, and although most bacteria contain cobamide-dependent enzymes, most are incapable of synthesizing the cofactors and need to source if from the environment [56, 57]. Cobalamin is an organometallic compound containing a central corrin ring with chelated cobalt. The biologically active form of cobalamin, adenosylcobalamin, can be synthesized by an aerobic or anaerobic pathway, with part of the pathway shared by both (Additional file 1: Fig. S3).

**Fig. 8:**
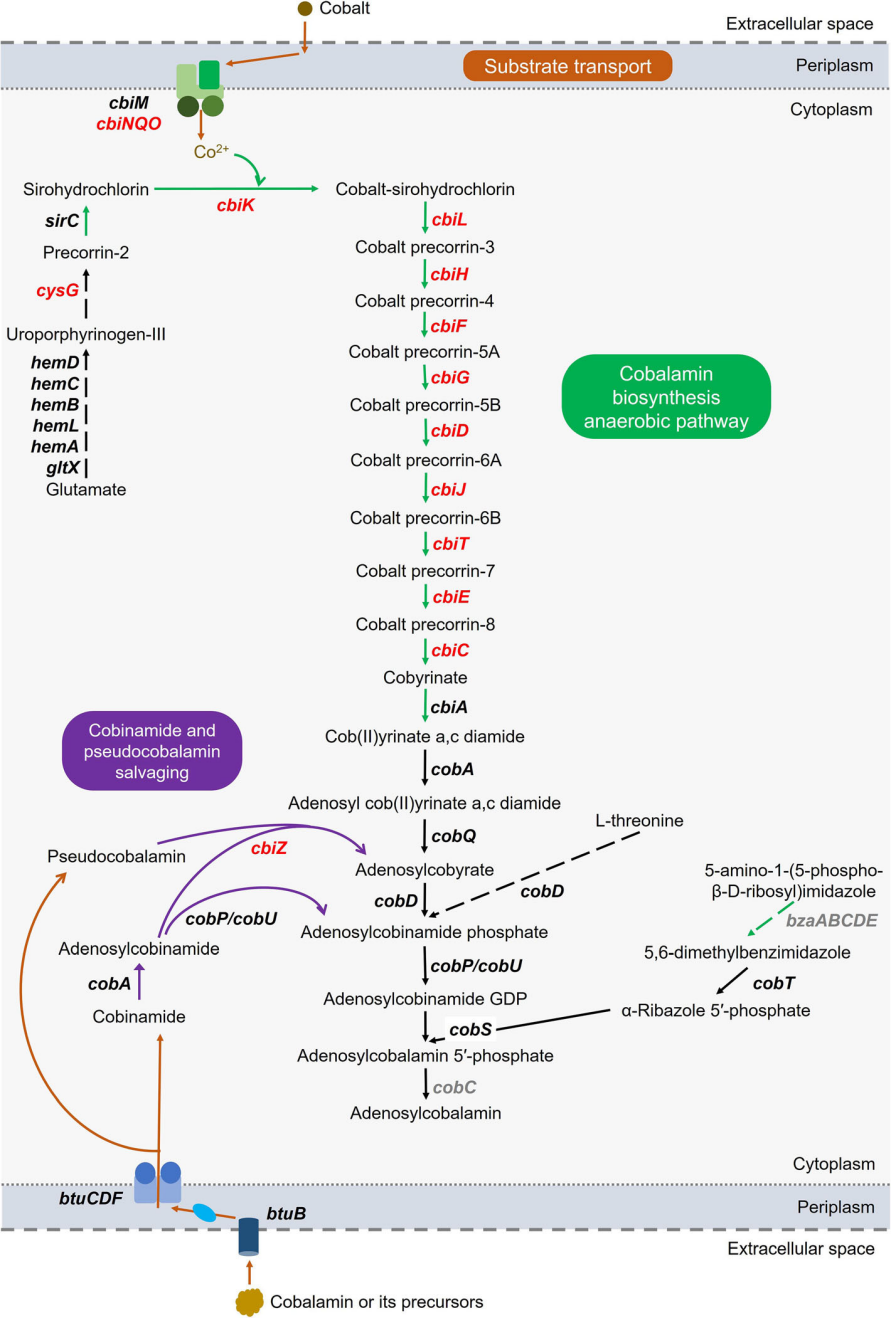
*Ca.* Chlorobium antarcticum cobalamin pathways. Steps common to the anaerobic and aerobic pathways, black connecting arrows; reactions specific to the anaerobic pathway, green arrows; cobinamide and pseudocobalamin salvaging, purple arrows; cobalt and cobalamin precursor transport, orange arrows; multi-step processes, dashed arrows connecting intermediate substrates; genes present in *Ca.* Chlorobium antarcticum, black font; genes not identified in *Ca.* Chlorobium antarcticum, grey font; genes in LCRs, red font. For additional details, see anaerobic and aerobic pathways genes in Additional file 1: Fig. S3; relative coverages for LCRs showing seasonal changes in abundance in Fig. 4, Additional file 1: Tables S4, S5, and S6; predicted bifunctional properties of genes in LCRs associated with anaerobic cobalamin production in Additional file 1: Table S6. Pathway information was derived from BioCyc online service [58, 59] and published data [60–67]. Cobalamin biosynthesis genes and enzymes listed in the order they function in the pathway: *gltX*, glutamyl-tRNA synthetase; *hemA*, glutamyl-tRNA reductase; *hemL*, glutamate-1-semialdehyde 2,1-aminomutase; *hemB*, porphobilinogen synthase; *hemC*, hydroxymethylbilane synthase; *hemD*, uroporphyrinogen-III synthase; *cysG*, uroporphyrin-III C-methyltransferase; *sirC*, precorrin-2 dehydrogenase; *cbiK*, sirohydrochlorin cobaltochelatase; *cbiL*, cobalt-precorrin-2 C20-methyltransferase; *cbiH*, cobalt-precorrin-3 C17-methyltransferase; *cbiF*, cobalt-precorrin-4 C11-methyltransferase; *cbiG*, cobalt-precorrin-5A hydrolase; *cbiD*, cobalt-precorrin-5B C1-methyltransferase; *cbiJ*, cobalt-precorrin-6A reductase; *cbiT*, cobalt-precorrin-6B C15-methyltransferase (decarboxylating); *cbiE*, cobalt-precorrin-7 C5-methyltransferase; *cbiC*, cobalt-precorrin-8 methylmutase; *cbiA*, cobyrinate A,C-diamide synthase; *cobA*, cobyrinate A,C-diamide adenosyltransferase; *cobQ*, adenosylcobyrate synthase; *cobD*, adenosylcobinamide-phosphate synthase; *cobP*/*cobU*, adenosylcobinamide kinase/adenosylcobinamide-phosphate guanylyltransferase; *cobS*, adenosylcobalamin 5′-phosphate synthase; *bzaAB*, 5-hydroxybenzimidazole synthase subunits A and B; *bzaC*, 5-hydroxybenzimidazole O-methyltransferase; *bzaD*, 5-methoxybenzimidazole C-methyltransferase; *bzaE*, anaerobic 5,6-dimethylbenzimidazole synthase; *cobT*, nicotinate-nucleotide dimethylbenzimidazole phosphoribosyltransferase; *cobC*, adenosylcobalamine-5′-phosphate phosphatase. Cobinamide and pseudocobalamin salvaging gene: *cbiZ*, adenosylcobinamide amidohydrolase. Cobalamin transport genes: *btuB*, outer membrane TonB-dependent transporter; *btuC*, ABC transporter permease subunit; *btuD*, ABC-transporter ATP-binding subunit; *btuF*, cobalamin-binding periplasmic protein. Cobalt transport genes: *cbiM*, ECF-transporter cobalt-binding component; *cbiN*, ECF-transporter transmembrane component; *cbiQ* and *cbiO*, ECF-transporter ATP-binding components

All the genes in the anaerobic pathway for cobalamin biosynthesis have been reported for *Chlorobaculum tepidum* [4]. However, a comparative genomics assessment of 11,000 bacterial species did not identify all cobamide biosynthesis genes in the 10 Chlorobi that were examined, including Cpv-DSM265, and categorized them as cobinamide salvagers [57]. We determined that *Ca.* Chlorobium antarcticum encodes the anaerobic pathway, with the genes exclusive to the anaerobic pathway (green-coloured branch between precorrin-2 and cob(II)yrinate a,c-diamide in Fig. 8) located in a LCR. At least 29% of the AL *Ca.* Chlorobium antarcticum population from all time periods, and 8% and 72% of the EF and TB *Ca.* Chlorobium antarcticum populations, respectively, possessed the genes, although coverage was about 2-fold higher in AL in summer compared to winter (Additional file 1: Tables S4 and S6).

The anaerobic synthesis of 5,6-dimethylbenzimidazole (DMB), the lower axial ligand of adenosylcobalamin, involves enzymes from the *bzaABCDE* operon acting on 5-amino-1-(5-phospho-β-D-ribosyl)imidazole as substrate [60]. While the *Ca.* Chlorobium antarcticum MAGs did not possess *bzaABCDE* or *cobC* it did encode the DMB activation and utilization genes (*cobT*, *cobS*). This indicates that similar to some other bacteria [68, 69], *Ca.* Chlorobium antarcticum may have a capacity to remodel exogenous DMB to produce cobalamin. The gene *cobC* can perform the final step in adenosylcobalamin synthesis, but *Ca.* Chlorobium antarcticum MAGs lacked this gene and may instead utilize alternative genes, *cblZ* or *cblXY,* which have been proposed to function in Actinobacteria and some Alphaproteobacteria, respectively [61].

The *Ca.* Chlorobium antarcticum LCRs also contained a colocalized cluster of genes annotated as cobaltochelatase subunit CobN and magnesium chelatase subunits BchH, BchI and BchD (Additional file 1: Table S6). CobN forms a complex with cobaltochelatase subunits CobS and CobT (which were not identified in the MAGs) and catalyses cobalt insertion during aerobic cobalamin biosynthesis [70, 71], and BchH, BchI and BchD can function in magnesium insertion during bacteriochlorophyll biosynthesis [72]. However, sequence similarity exists between cobaltochelatase NST and magnesium chelatase HID [73, 74] and it has been speculated that BchI and BchD may function as CobS and CobT to form a functional cobaltochelatase complex [61]. In *Ca.* Chlorobium antarcticum, these cobalt/magnesium chelatase genes were colocalized with potential cobalamin transport genes (LCR5 in Additional file 1: Table S4; Groups 4 and 5 in Additional file 1: Table S5) and therefore may function in cobalamin biosynthesis. In support of this inference, it was speculated that the colocalization of cobalt/magnesium chelatases beside a TonB-dependent receptor protein for cobalamin in *Chlorobaculum tepidum* may pertain to cobalt being inserted into exogenously acquired cobalamin [4]. Moreover, additional magnesium chelatase genes, including three coding for BchH and one each for BchI and BchD, were present throughout the *Ca.* Chlorobium antarcticum population which likely function in bacteriochlorophyll synthesis rather than cobalamin production. Most GSB contain three homologues of BchH, denoted BchH, BchS, and BchT [75], which have been reported to be active magnesium chelatases that exhibit differences in their enzymatic properties [76].

Cobalamin biosynthesis genes can be colocalized with the cobalt transporter genes *cbiMNQO* [61, 62], and this was the case in *Ca.* Chlorobium antarcticum (LCR5 in Additional file 1: Table S4). Cobalt is relatively concentrated in AL, with ~6 nM at the oxic-anoxic interface which is ~ 300-times the concentration in sea water [30, 43]. The *cbiMNQO* gene cluster was present in a LCR (Group 6 in Additional file 1: Table S5) with the genes present in at least 41% of the *Ca.* Chlorobium antarcticum population from all time periods, although an approximately 1.5-fold higher coverage occurred in summer compared to winter; the minimum abundance (~ 30%) and seasonal change (~ 2-fold higher in summer) are similar to the phylotypes containing the cobalamin biosynthesis genes.

The *Ca.* Chlorobium antarcticum MAGs contained *cobA*, *cobP*/*cobU*, and *cbiZ*, representing all the genes known in bacteria and archaea to be involved in salvaging cobinamide [63–66]. *cbiZ* can also function in salvaging pseudocobalamin, and *cbiZ* was the only gene located in a LCR (Fig. 8; Additional file 1: Table S6). These data indicate that the whole lake population of *Ca.* Chlorobium antarcticum was likely adept at converting cobinamide into intermediates of cobalamin biosynthesis, and a subpopulation (at least 8% from all time periods) had the capacity to also salvage pseudocobalamin. The coverage of *cbiZ* was about 2-fold higher in summer, matching the seasonal abundance pattern of cobalt transporter and cobalamin biosynthesis genes (Additional file 1: Tables S5 and S6).

In *Ca.* Chlorobium antarcticum MAGs, the *cbiZ* and cobalamin transporter genes were colocalized (LCR5 in Additional file 1: Table S4), as is the case in many bacteria, including *Chlorobium* [65]. It has been speculated that *Rhodobacter sphaeroides* may use cobalamin transporters to scavenge pseudocobalamin produced by cyanobacteria and convert it to cobalamin precursors using CbiZ [65, 66, 77–80]. AL supports a high abundance of *Synechococcus* that blooms in summer close to the oxic-anoxic interface [19, 81], indicating that it may be the source of pseudocobalamin that is imported and converted to cobalamin precursors by *cbiZ*.

The uptake of cobalamin itself requires TonB-dependent transport (BtuB) through the outer membrane and ABC transporters (e.g., BtuCDF) or energy-coupling factor (CbrT) through the inner membrane [82–84]. *Ca.* Chlorobium antarcticum contained two putative *btuB* TonB-dependent transporter genes, plus a set of ABC transporter genes (*btuC*, permease; *btuD*, ATP-binding; *btuF*, substrate-binding) throughout the population. Additional putative *btuB* and *btuCDF* genes were also present in LCRs (Groups 3, 4, and 5 in Additional file 1: Table S5) in at least 7% of the *Ca.* Chlorobium antarcticum population across all time periods, although the abundance was 2–3-fold higher in summer compared to winter (Groups 3, 4, and 5 in Additional file 1: Table S5).

The biosynthesis and transport of cobalamin has been shown to be regulated by cobalamin-binding riboswitches that are present in the 5′-untranslated region of genes, including *btuB* (cobalamin transporter), *metE* (5-methyltetrahydropteroyltriglutamate homocysteine methyltransferase), and *nrdD* (ribonucleoside-triphosphate reductase) [85–93]. A total of six cobalamin riboswitch sequences were identified in LCRs of *Ca.* Chlorobium antarcticum, one each upstream of *btuB* and *btuF* (both cobalamin transporters), *metE*, *nrdD*, and at the end of two contigs (Fig. 4b; Additional file 1: Table S6). Three additional cobalamin riboswitch sequences were identified throughout the *Ca.* Chlorobium antarcticum population, one each upstream of two *btuB* genes, and a hypothetical protein-coding gene. In Chlorobi, the genes with cobalamin riboswitch sequences are mainly translationally regulated; regulation has been shown to involve inhibition of translation initiation, where cobalamin (in the form of adenosylcobalamin) binds to the riboswitch RNA sequence of the regulated mRNA, leading to a perturbed mRNA structure that inhibits ribosome binding and subsequent translation [88, 89, 91].

Overall, the phylotype data for cobalamin-related biosynthesis, salvaging, and transport indicate that all of the *Ca.* Chlorobium antarcticum population is capable of importing cobalamin (Additional file 1: Tables S4, S5, and S6), although the proportion of the population with additional cobalamin transport genes varies with the system: EF, 7%; AL, 7% increasing to 25% in summer; TB, 78% (Additional file 1: Tables S4 and S5). Certain phylotypes are also capable of importing and salvaging cobinamide and pseudocobalamin, with this capacity also increasing in summer in AL.

### *Ca.* Chlorobium antarcticum-virus interactions

The subtype I-E CRISPR-Cas system in *Ca.* Chlorobium antarcticum contained the core *cas* genes *casA* (or *cse1*) and *casB* (or *cse2*) with genes arranged *cas3*, *casA*, *casB*, *casE*, *casC*, *casD*, *cas1*, *cas2*, followed by a CRISPR spacer array, indicating the system could be functional. Analysis of NCBI gene annotation data showed CRISPR-Cas systems to be common in GSB, the subtypes to vary, and some species to contain multiple subtypes (Additional file 1: Table S8). No genes associated with BREX (bacteriophage exclusion) or DISARM (defence island system associated with restriction-modification) systems were identified. However, type I R-M (restriction-modification) methyltransferase and endonuclease and two type IV R-M endonuclease genes were identified (Additional file 1: Table S9), with the type I R-M genes present in a LCR (Additional file 1: Tables S4). Additionally, five genes associated with toxin-antitoxin (T-A) systems (*parD*, *parE*, *relF*, *brnA*, *abiEi*) were identified in *Ca.* Chlorobium antarcticum (Additional file 1: Table S9), with *brnA* in a LCR (Additional file 1: Table S4). The most likely system to contribute to the control of viral propagation is the AbiE type IV T-A system, an ABI (abortive infection) system that causes cell dormancy and prevents viral dissemination [94], but it is unclear if this system was functional as the antitoxin gene (*abiEi*) was identified but not the toxin gene (*abiEii*).

Potential *Ca.* Chlorobium antarcticum viruses were identified by aligning the *Ca.* Chlorobium antarcticum CRISPR-Cas spacers to an Antarctic virus catalogue, and a spacer database was used to identify additional potential hosts of the viruses (see the “Methods” section) [19]. A total of 79 CRISPR spacers from EF *Ca.* Chlorobium antarcticum MAGs (Additional file 1: Table S10) mapped to potential viruses. Eight viral contigs had 97% identity to spacer Spc230 (Additional file 1: Table S11). The viral contigs were from AL metagenomes and belonged to viral cluster cl_248, a previously identified potential AL *Chlorobium* virus [19]. No EF *Ca.* Chlorobium antarcticum spacers were mapped to EF viral contigs, which likely reflects the smaller size of the EF metagenome dataset compared to AL which resulted in 6,104 EF viral contigs compared to 30,897 AL viral contigs in the Antarctic virus catalogue.

**Fig. 9:**
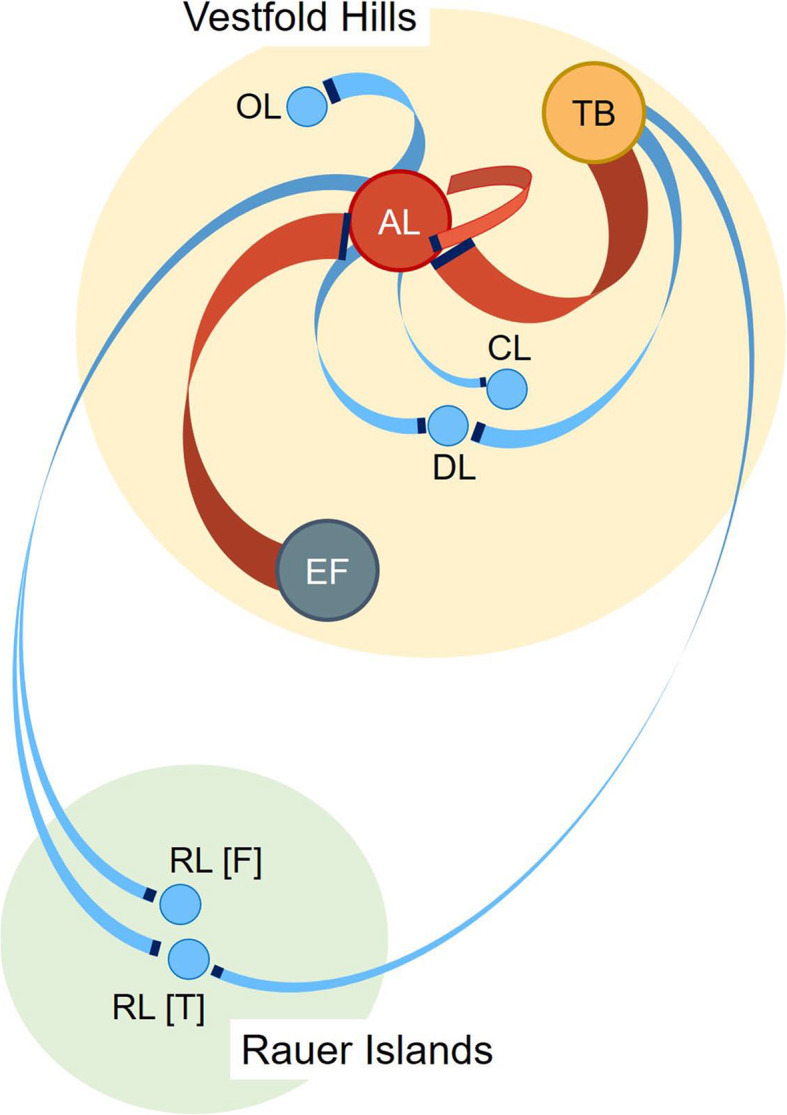
Biogeographic association between viral contigs and *Ca.* Chlorobium antarcticum CRISPR spacers. The schematic depicts the Vestfold Hills and Rauer Islands systems that were the sources of the viral contigs that matched to *Ca.* Chlorobium antarcticum CRISPR spacers (Additional file 1: Table S11). Lines (red or blue) connect an aquatic system where CRISPR-spacers were identified to a system where matching viral contigs were identified. The width of a line (red or blue) approximates the number of spacer-viral contig matches. The dark blue end of a line (red or blue) denotes the system that was the source of the viral contigs, with the other end of the line being the source of the *Ca.* Chlorobium antarcticum CRISPR-spacers. Spacer-viral contig matches within the three systems harbouring *Ca.* Chlorobium antarcticum (AL, EF, and TB; red lines) are distinguished from spacer-viral contig matches between AL, EF, or TB, and the other aquatic systems in the Vestfold Hills and Rauer Islands (blue lines). Sources of *Ca.* Chlorobium antarcticum spacers are denoted by large circles: AL (), EF (), and TB (); other lakes are denoted by small circles (). Sources of viral contigs included: AL, DL (Deep Lake), CL (Club Lake), OL (Organic Lake), RL(F) (Rauer Lakes from Filla Island: RL2, 3, 11), RL(T) (Rauer Lakes from Torckler Island: RL5, 6, 13). The location of the systems relative to each other is shown approximately to scale

As the TB metagenomes were not available when the Antarctic virus catalogue and IMG/VR spacer database were constructed [19], a slightly different approach was used to identify viral contigs matching to spacers in TB *Ca.* Chlorobium antarcticum MAGs (see the “Methods” section). A total of 58 TB *Ca.* Chlorobium antarcticum spacers were aligned against the Antarctic virus catalogue, resulting in nine spacers (Spc236, Spc238, Spc241, Spc243–Spc245, Spc249, Spc251, Spc252; Additional file 1: Table S10) matching to 23 viral contigs with ≥ 97% identity. Eighteen of the viral contigs were from AL metagenomes and belonged to viral cluster cl_1024 (14) and viral singletons sg_10581 (1), sg_14551 (1), sg_14796 (1), and sg_14959 (1); cl_1024 was previously identified as a potential AL *Chlorobium* virus [19]. The remaining five viral contigs were from hypersaline Antarctic systems, Deep Lake and Rauer 13 Lake [41], and belonged to cl_9176 (1), sg_1370 (1), sg_1648 (1), sg_1649 (1), and sg_1677 (1). Similar to EF, no TB *Ca.* Chlorobium antarcticum spacers mapped to the 995 available TB viral contigs, likely reflecting the size of the metagenome dataset. It is noteworthy that the AL *Ca.* Chlorobium antarcticum spacers themselves had ≥ 97% identity matches to viral contigs from AL as well as Deep Lake, Club Lake, Organic Lake, and some Rauer Island lakes (Rauer 2, 3, 5, 6, 11, and 13 lakes) (Fig. 9; Additional file 1: Table S11).

The viral contigs representing potential EF and TB *Ca.* Chlorobium antarcticum viruses were matched (100% identity) to host spacers, identifying potential hosts to be primarily Gammaproteobacteria and Chlorobi (including *Chlorobium* OTUs from the Vestfold Hills), plus Actinobacteria, Bacteroidetes, Firmicutes, Betaproteobacteria, Deltaproteobacteria, and Verrucomicrobia (Additional file 1: Table S12). These host assignments were similar to previous findings for AL *Chlorobium* viruses [19] and point to *Ca.* Chlorobium antarcticum viruses from all three systems belonging to similar viral clusters (e.g., cl_1024 and cl_248). This host analysis indicates that the viruses likely prey on several different bacterial genera as a wide variety of hosts, and may therefore be considered generalist rather than specialist viruses [95–97].

The predicted *Ca.* Chlorobium antarcticum viruses also appeared to be widely distributed with spacer matches to viral contigs from hypersaline systems enriched in haloarchaea (Deep Lake, Club Lake, Rauer 3, 6, and 13 lakes) and diverse bacterial taxa (Organic Lake, Rauer 2, 5, and 11 lakes) (Fig. 9; Additional file 1: Table S11). *Chlorobium* has not been reported in these lake systems, and the microbial communities in Deep Lake [38, 41] and Organic Lake [98, 99] in particular, have been intensively studied. In contrast, the other potential hosts, notably Gammaproteobacteria, are prevalent in Organic Lake [98, 99] and have been identified in some of the other lakes [38, 41], further reinforcing that the potential *Ca.* Chlorobium antarcticum viruses have characteristics of generalist viruses infecting a broad host range [95–97].

## Conclusions

We have shown that a single species of *Chlorobium* was detected in AL, EF, and TB that has distinct genomic traits to its closest relative Cpv-DSM265 (Additional file 1: Table S13) and is not identifiable in available metagenome data from elsewhere in the world. As such, we conclude that *Ca.* Chlorobium antarcticum is to the best of our knowledge, endemic to the stratified lakes and fjords of the Vestfold Hills of East Antarctica.

Variation present as SNPs and LCRs defined population variation of *Ca.* Chlorobium antarcticum, indicating the presence of phylotypes and ecotypes, with the population structure differing marginally amongst the three systems. Limited genomic variation of *Ca.* Chlorobium antarcticum in AL across a 7-year period illustrates that the population is currently stable. Seasonal changes in population structure were inferred to arise as a natural response to sunlight hours and growth of active populations. Population variation contributing to survivability was inferred for genes associated with cold adaptation, metabolism, and viral defence. In particular, cobalamin synthesis and transport stood out as a genomic facet of *Ca.* Chlorobium antarcticum that was subject to seasonal variation in population structure and was likely a trait relevant to effective ecosystem functioning.

Cobalamin deficiency can impair bacteriochlorophyll content and chlorosome formation, with cobalamin supplementation restoring bacteriochlorophyll content [100, 101]. The higher abundance in summer (cf. 2-fold higher than winter) of *Ca.* Chlorobium antarcticum phylotypes that possess a genomic capacity for cobalamin biosynthesis, cobinamide and pseudocobalamin salvaging, cobalt transport, and/or cobalamin transport, fits with the importance of cobalamin for supporting phototrophic processes and may help cells recuperate after a long, dark winter to regain the very high abundance they achieve in summer. Conversely, the involvement of ~ 30 genes and energetic cost associated with cobalamin biosynthesis [56] fits with the ecosystem supporting a reduced capacity in winter when sunlight is limited or absent. While bacteria rely on cobalamin for growth, most bacteria in microbial communities lack the biosynthetic capacity [56, 57]. *Ca.* Chlorobium antarcticum is the most abundant species in AL and is key to ecosystem function, being probably the single most important member of the food web [19]. Its requirement for cobalamin for effective phototrophic growth likely generates positive selection within the *Ca.* Chlorobium antarcticum population for a biosynthetic capacity. As a result of its niche competitiveness, the species generates a very high level of biomass mid-water in the lake (>10^8^ cells ml^−1^) [14]. Therefore, in addition to its role in carbon, nitrogen, hydrogen, and sulphur cycling [13, 14, 19], *Ca.* Chlorobium antarcticum is also likely to be the main provider of exogenous cobalamin to the lake food web; this provision would be facilitated by the seasonal lysis and release of cellular contents of > 99% of the summer population of cells.

Partially based on *Chlorobium*-virus interactions in AL, it was proposed that some Antarctic viruses may persist by achieving less harmful interactions with their hosts than counterparts from warmer environments [19]. However, *Chlorobium*-virus interactions are not well understood because very few GSB viruses have been described [17, 102]. Through this study and a previous study [19], a total of 59 viral contigs and 12 viral clusters or singletons were mapped to *Ca.* Chlorobium antarcticum CRISPR-spacers, resulting in the discovery of 12 potential *Chlorobium* viruses. These viruses are predicted to be generalists. It has been speculated that viruses can evolve into specialist viruses when they are exposed to a homogenous host population (e.g., composed of a single species) that does not change with time, whereas generalist viruses can evolve from viruses exposed to a heterogenous host population (e.g., composed of multiple species) that fluctuates with time [95]. The adaptation of a specialist virus to effective replication in a single host may result in a cost to fitness when replicating in other potential hosts, whereas a generalist virus is not expected to suffer a fitness cost as it is adapted to replicate in different hosts [95]. While *Ca.* Chlorobium antarcticum represents a remarkably dominant species with relatively subtle population variation and may therefore be expected to harbour specialist viruses, its seasonal abundance in AL changes by at least 100-fold [19]. If as proposed, sunlight hours control seasonal abundance of AL *Chlorobium* [19], the marked change in host abundance may select against the establishment of specialist viruses, while still leaving *Ca.* Chlorobium antarcticum as a host for generalist viruses that have a capacity to propagate in other bacterial hosts. In this regard, a reliance on sunlight and seasonal die-off during winter and early spring may significantly benefit the long-term persistence of *Ca.* Chlorobium antarcticum in Antarctic aquatic systems.

The Antarctic continent is geographically isolated and Antarctic environmental conditions distinguish it from most other regions of the globe [27, 103, 104]. The remoteness and environmental conditions create major logistical challenges for performing scientific research, yet without adequate research, policy makers will be compromised when making decisions about Antarctica’s future [104]. Metagenomic approaches have greatly enhanced the understanding of indigenous Antarctic microorganisms [27, 103]. For example, Antarctic soil bacteria were discovered that scavenge and oxidize atmospheric H_2_, which in association with CO and/or CO_2_, enables chemosynthetic growth [105]. In the Vestfold Hills and Rauer Islands, three different genera have been found to dominate the haloarchaea population of hypersaline lakes, making photoheterotrophy the main microbial process occuring in these lakes [38, 40, 41, 106]. The species appear to be endemic to Antarctica, with one member, *Halohasta litchfieldiae* (tADL), constituting ≤ 45% of each lake’s microbial community [38, 41]. Relatively little genomic variation exists within and between the populations from the hypersaline systems, but both environment and distance effects have been inferred to contribute to biogeographical patterning of variation [41]. A major phylotype of *Hht. litchfieldiae* with relatively low ANI (~ 0.8) has also been discovered [38, 40]. Based on our current research, we make the claim that *Ca.* Chlorobium antarcticum represents the Antarctic species with the least amount of known population-level, genomic variation. The capacity to state this is predicated on having a very large *Ca.* Chlorobium antarcticum metagenome dataset (~ 159 Gb) that provided a MAG read depth of up to ~ 11,000. The coherence of the population is particularly striking in view of it being retained across a 7-year time span, across the populations from three distinct water bodies, and throughout the population of a seasonal cycle, during which relative cellular abundance changed > 100-fold. Future efforts need to evaluate how distinct Antarctic species and communities are by canvassing the environmental and biogeographic diversity of Antarctica’s ecosystems and obtaining sufficient metagenomic depth to assemble MAGs and perform population-level studies. Achieving this will help to establish the extent of Antarctic microbial endemism, the uniqueness of contributions that Antarctic microbes make to global biogeochemical cycles, and the risks associated with anthropogenic impact, including climate change, on the Antarctic biome [27, 104, 107].

## Methods

### Sample collection, DNA sequencing, MAG generation, and abundance calculations

The sampling, sequencing, assembly, and annotation of AL metagenomes were described previously [19, 108]. Biomass from EF Basin 2 (Fig. 1) was collected from 5-, 18-, 45-, and 60-m depths by filtration through a 20-μm prefilter onto large (293 mm diameter) format filters (3, 0.8 and 0.1 μm) and DNA extracted as previously described [13, 38, 41]. The sequencing, assembly, and annotation of EF metagenomes were performed by the Joint Genome Institute as previously described [19], generating 12 EF metagenomes (three filter fractions from four depths) (Additional file 1: Table S1). The biomass from TB Basin 1 (Fig. 1) was collected from 5- and 11-m depths by filtration through a 20-μm prefilter into Sterivex cartridges (0.22 μm filter) and the DNA extracted and sequenced as previously described [108] (Additional file 1: Table S1). The QC filtered and error-corrected reads (BFC v181) [109] from the AL, EF, and TB metagenomes were assembled using metaSPAdes [110, 111] and annotated through IMG (Additional file 1: Table S1). The IMG pipeline generated *Ca.* Chlorobium antarcticum MAGs, of which we used 50 AL, seven EF, and two TB MAGs (one MAG per metagenome) that were medium to high quality and > 50% genome completeness; the MAGs with their respective metagenomes are available in IMG (see IMG Bin IDs in Additional file 1: Table S2; Additional file 2: Dataset S1). For MIMAG (minimum information about MAGs) [112] data preparation, MAG quality data and metadata were obtained from IMG, except MAG N50 and L50 contig statistics which were generated using Quast v5.0.2 [113] (Additional file 2: Dataset S1). *Chlorobium* OTU abundances from AL were calculated previously [19]. Contig taxonomy assignments, *Chlorobium* OTU bin refinement, abundance calculations, and alpha diversity (Simpson’s index of diversity) from EF and TB metagenomes were determined as previously described [19].

### *Ca.* Chlorobium antarcticum genomic variation

The metagenome reads from the oxic-anoxic interface of AL, EF, and TB were used for FR analyses of *Ca.* Chlorobium antarcticum (Additional file 1: Table S14). The AL metagenomes used were all Illumina data and represented two sampling periods (2008 and 2013–2014), including different seasons: summer (Dec 2014), winter (Jul and Aug 2014), and spring (Nov 2008, Nov 2013, Oct 2014). AL metagenomes from 2006 were not included due to possible bias caused by differences in dataset size (2006, ≤ 500 million bases; 2008 and 2013/2014, ≥ 3 billion bases) and sequencing technology used (2006, Sanger and 454; 2008 and 2013/2014, Illumina). However, it is noteworthy that *Chlorobium* abundance in AL in 2006 was previously shown to be comparable to 2008 and 2013/2014 [19], so inferences from this study are likely to apply to the 2006 population.

The AL and EF reads from the three filters from a specific time period and depth were pooled and converted to multi-FASTA format using an in-house script, thereby facilitating comparative analyses between AL and EF metagenomes (biomass in the size range 0.1–20 μm) with TB metagenomes (0.22–20-μm biomass size range) (Additional file 1: Table S14). For the analysis of genomic variation within the AL *Ca.* Chlorobium antarcticum population, the MAG from Dec 2014, 19-m depth, 0.1-μm filter was used (AL_ref MAG). For analyses between AL, EF, and TB, the EF *Ca.* Chlorobium antarcticum MAG from 45-m depth, 3-μm filter was used (EF_ref MAG). The two MAGs were selected because they had the highest total base pair count and > 99% genome completeness. To determine the *Ca.* Chlorobium antarcticum MAG contig arrangement that best represents a draft genome, the AL_ref MAG and EF_ref MAG contigs were organised in Mauve v2.4.0 [114] using Cpv-DSM265 as the reference genome with default parameters. Contigs were subsequently manually reordered by comparing nucleotide sequences from AL, EF, and TB using the blastn module of BLAST+ v2.9.0 [115] and considering only ≥ 500-bp alignment length matches of 100% identity. Arising from this, MAG contigs were grouped into scaffolds (Additional file 1: Table S3).

The metagenome reads were aligned to AL_ref MAG or EF_ref MAF using BBMap v38.51 [116] with 95% minimum alignment identity (minid = 0.95), generating SAM files. The BAM and BAI alignment and index files were created from SAM files using Samtools v1.10 [117] and were used for SNP analysis in IGV [118]. Only the SNPs with variant frequency ≥ 0.9 (i.e., at least 90% of the reads aligned at the position containing the SNP) were considered fixed mutations, similar to a previously described method [38]. The total number of aligned reads and the base coverages of AL_ref MAG and EF_ref MAG were calculated using the “flagstat” and “depth” functions of Samtools, respectively. To identify LCRs, the base coverages of AL_ref MAG and EF_ref MAG in metagenomes from AL, EF, and TB were plotted on circos plots using R v4.0.2. The LCRs that spanned multiple adjacent contigs on a scaffold were considered a single LCR (Additional file 1: Table S4); for example, LCR5 spanned contigs A13–A17 from AL_ref MAG and contigs E14–E15 from EF_ref MAG. The IMG auto-annotated genes identified in LCRs were manually annotated by aligning the protein sequences to reference proteins from the UniProtKB/Swiss-Prot database using the ExPASy BLAST+ online service [119], and those with poor alignment or no hits were realigned to reference proteins in the UniProtKB database or RefSeq protein database using the NCBI blastp suite [120].

For comparison of gene order between AL_ref MAG and other AL, EF, and TB high-quality MAGs of ≥ 99% genome completeness, the MAG contigs were aligned using the blastn module of BLAST+ v2.9.0. The alignments were manually parsed to assess the gene order of MAGs compared to that of AL_ref MAG, and MAG contigs that did not align, had lower identity matches (< 80%) or short length matches (< 1 kb) were identified (Additional file 3: Dataset S2).

### GC content vs read depth analysis

Based on an approach previously reported for analysing haloarchaea [38], metagenome contigs of length ≥ 1 kb, and 30–70% GC content, and *Ca.* Chlorobium antarcticum MAG contigs from AL, EF, and TB, were plotted in a GC content-read depth 2D space using Python v3.6.4. The metagenome contig clusters placed close to the *Ca.* Chlorobium antarcticum MAG contig cluster that had a GC content ranging from 35–65% and read depth up to 7500 and length ≥ 10 kb, were selected for taxonomic analysis. The contigs were aligned to the *Ca.* Chlorobium antarcticum MAGs and Cpv-DSM265 genome. The alignment files were manually parsed to identify cluster contigs with low identity and high query alignment fraction (≥ 5 kb), and their taxonomies were determined using the IMG Phylodist file-based contig taxonomies, as described previously [19]. Some small clusters of metagenome contigs were from *Ca*. Chlorobium antarcticum (Additional file 1: Fig. S2c), with 100% identity to *Ca*. Chlorobium antarcticum MAG contigs. These metagenome contigs likely belonged to two incomplete *Ca*. Chlorobium antarcticum MAGs (60% and 66% bin completeness) generated from 0.8–3- and 0.1–0.8-μm filter Nov 2008 spring metagenomes, respectively, from AL oxic-anoxic interface.

### *Ca.* Chlorobium antarcticum phylotype abundance

The *Ca.* Chlorobium antarcticum population containing a “region of interest” (specific LCR, gene, or gene cluster) was determined from the relative coverages of the corresponding region, calculated using the formula:

$$\frac{Mean\kern0.17em read\;{depth}_{\left(\mathit{\operatorname{Re}} gion\right)}}{Mean\kern0.17em read\;{depth}_{(MAG)}}\times 100$$

where *Region* is the region of interest and *MAG* is AL_ref MAG or EF_ref MAG. The numerator indicates the mean read depth of the region of interest in a metagenome and the denominator refers to the mean read depth of the MAG in the metagenome.

The mean read depths were calculated using the formula:

$$\frac{\sum_{\left(\mathit{\operatorname{Re}} gion/ MAG\right)}\mathit{\operatorname{Re}} ad\kern0.5em depth\kern0.5em of\kern0.5em bases}{Total\kern0.17em number\kern0.17em of\;{bases}_{\left(\mathit{\operatorname{Re}} gion/ MAG\right)}}$$

where *Region* is the region of interest and *MAG* is AL_ref MAG or EF_ref MAG. The numerator indicates the sum of the read depths of the bases in a region of interest or MAG, calculated in each metagenome. The denominator indicates the total number of bases in the region of interest or MAG.

The approximate percentage of the *Ca.* Chlorobium antarcticum population containing a region of interest, in a season (summer, winter, spring) or a system (AL, EF, TB) were determined by averaging the percentages calculated in metagenomes from a season or a system, respectively. To assess the significance of the differences in summer and winter coverages of LCR genes of AL_ref MAG, the DESeq2 R package [121] was used with gene read depths from all time periods. The result for summer and winter comparison was generated using the “contrast” option of DESeq2 result function. DESeq2 method uses Wald test to calculate the *P*-value for significance analysis and uses Benjamini-Hochberg adjustment to calculate adjusted *P*-value for assessing significance considering a specific false discovery rate (i.e., the fraction of false positives amongst the significant values). Here, *P*-values < 0.05 were considered significant at the 95% significance level. BH-adjusted *P*-values < 0.05 were regarded as significant, considering a 5% fraction of false positives as acceptable (Additional file 1: Tables S5 and S6).

### Comparative analysis of *Ca.* Chlorobium antarcticum and Cpv-DSM265

A total of 31 AL, five EF and two TB *Ca.* Chlorobium antarcticum MAGs with ≥ 99% genome completeness were aligned to the Cpv-DSM265 genome (RefSeq ID: NC_009337.1) using the blastn module of BLAST+ v2.9.0 and Samtools v1.10, generating SAM, BAM, and BAI files. The alignments were analysed using IGV to assess the types of variations (indels or SNPs) in MAG sequences. The auto-annotated genes on MAG contigs or Cpv-DSM265 genome that showed no alignment were assessed. To identify cobalamin riboswitch sequences in *Ca.* Chlorobium antarcticum, four cobalamin riboswitch genes from the Cpv-DSM265 genome were aligned to AL_ref MAG contigs using the NCBI blastn suite [120]. The *Ca.* Chlorobium antarcticum cobalamin riboswitch sequences were verified, and additional cobalamin riboswitch sequences were identified, using the Rfam database [122, 123] (Additional file 1: Table S6). The overall functional potential of Cpv-DSM265 and *Ca.* Chlorobium antarcticum MAGs from AL (AL_ref MAG), EF (EF_ref MAG), and TB (MAG from TB 11-m depth metagenome) were compared using COG number data generated by IMG. The COG numbers were categorized using COG reference data from IMG (database accessed on 21 December 2020). Genes with COG number assignments belonging to more than one COG category were assigned to multiple categories (Additional file 1: Fig. S4).

### ANI, AAI, and phylogenetic analyses

The pair-wise ANI of *Ca.* Chlorobium antarcticum MAGs, as well as ANI against the Cpv-DSM265 genome were calculated using pyani [124]. The AAI of MAGs was calculated using the AAI-profiler online service [125], which compared the input protein sequences with the proteins of species in the UniProt database [126]. The phylogenetic analysis of *Ca.* Chlorobium antarcticum was performed using the 16S rRNA gene and FmoA protein sequences from AL, EF, and TB MAGs, as well as various members of the Chlorobiaceae family (Additional file 1: Table S15). The 16S rRNA genes were aligned using the ClustalW algorithm and FmoA proteins were aligned using the Neighbour Joining cluster method of the MUSCLE algorithm in MEGA X v10.1.7 [127]. The alignments were used for generating maximum likelihood trees in MEGA using default parameters and 1000 bootstrap values.

The proportion of the *Chlorobium* population that was represented by *Ca.* Chlorobium antarcticum in the AL, EF, and TB oxic-anoxic interface metagenomes was estimated by aligning AL, EF and TB metagenome reads to the *Ca.* Chlorobium antarcticum 16S rRNA gene from EF_ref MAG using BBMap v38.51 and Samtools (see above in “*Ca.* Chlorobium antarcticum genomic variation”). The default minid was used for alignment with BBMap. SNPs with variant frequency ≥ 0.01 (i.e., at least 1% of the reads aligned at the position containing the SNP) were considered during analysis in IGV (Additional file 3: Dataset S2).

Assessment of the endemism of *Ca.* Chlorobium antarcticum to the Vestfold Hills was performed by comparing *Ca.* Chlorobium antarcticum marker (16S rRNA gene and FmoA protein) sequences to available metagenome and genome data in IMG. The *Ca.* Chlorobium antarcticum 16S rRNA gene was aligned to the IMG databases of 16S rRNA genes from public-assembled metagenomes (accessed on 14 Mar 2021) and public isolates (accessed on 30 Mar 2021) using the IMG RNA BLAST (blastn) online service with *e*-value 10^−5^. The *Ca.* Chlorobium antarcticum FmoA protein sequence was aligned to the IMG isolate protein database (including proteins from isolate genomes, MAGs, and single-amplified genomes; accessed on 14 Mar 2021) using the IMG RNA BLAST (blastp) online service with *e*-value 10^−5^.

### *Ca.* Chlorobium antarcticum defence genes and associated viruses

The AL, EF, and TB *Ca.* Chlorobium antarcticum MAG genes were manually parsed to identify those associated with defence, such as R-M, DISARM, BREX, and T-A (specifically ABI mechanism) systems. The putative defence genes were manually annotated (see above in “*Ca.* Chlorobium antarcticum genomic variation”).

The potential viruses associated with EF and TB *Ca.* Chlorobium antarcticum were determined using the CRISPR spacers and repeats in metagenome IMG CRISPR annotation files, as well as the data in an Antarctic virus catalogue and IMG/VR spacer database, as described previously [19]. The Antarctic virus catalogue contained a list of viral contigs identified in a range of Antarctic metagenomes, along with their viral cluster or singleton designations, and the IMG/VR spacer database contained a list of spacer sequences and their matches to host contigs [128]; the construction of these two databases was described previously [19]. The databases did not include TB metagenome data as these metagenomes were not available at the time the databases were created. To identify TB viral contigs, all TB assembled contigs were aligned to the Antarctic virus catalogue using the blastn module of BLAST+ v2.9.0, with *e*-value 10^−3^ and ≥ 97% alignment identity. A total of 995 TB contigs with ≥ 1000-bp alignment length and 100% identity across the whole length of either the query contig or the reference viral contig were considered to be TB viral contigs; this approach to identifying TB viral contigs from matches to the Antarctic virus catalogue is not as rigorous as might be achieved using the virus identification pipeline [129].

The *Ca.* Chlorobium antarcticum CRISPR spacers in EF and TB metagenomes were identified from the *Ca.* Chlorobium antarcticum MAGs and *Chlorobium* OTU refined bins (Additional file 1: Table S10). CRISPR arrays tended to be present at the ends of contigs, possibly indicative of assembly constraints caused by sequence repeats. To potentially capture a greater number of spacers, TB MAGs derived from assembly of non-error corrected reads (IMG Genome IDs: 3300038786, 3300039186) were also analysed. The viral contigs potentially associated with EF and TB *Ca.* Chlorobium antarcticum were determined by aligning the *Chlorobium* spacer sequences to viral contigs in the Antarctic virus catalogue and to TB viral contigs using the ‘megablast’ option of BLAST+ v2.9.0, with *e*-value 10^−3^ and ≥ 97% alignment identity. The data in the Antarctic virus catalogue were used to assign viral cluster or singleton designations to the potential *Ca.* Chlorobium antarcticum viral contigs. This approach to assessing virus-host relationships was described previously [19].

## Supplementary Information

**Additional file 1.** Data on *Ca*. Chlorobium antarcticum population structure, genomic variation, and viral analysis. Supplementary text, figures, and tables.

**Additional file 2.** MIMAG data for *Ca*. Chlorobium antarcticum. Supplementary dataset.

**Additional file 3.** Data from *Ca*. Chlorobium antarcticum 16S rRNA gene variation analysis and comparative analysis of AL, EF and TB MAGs and Cpv-DSM265 genome. Supplementary dataset.

**Additional file 4.** Data for *Ca*. Chlorobium antarcticum marker gene and protein analyses. Supplementary dataset.

## Data Availability

All metagenomes and medium and high-quality MAGs used in this study are available in IMG: see details in Additional file 1: Tables S1 and S2 and Additional file 2: Dataset S1.

## References

[1] Pfennig N, Trüper HG (1971). Higher taxa of the phototrophic bacteria. Int J Syst Bacteriol.

[2] Sakurai H, Ogawa T, Shiga M, Inoue K (2010). Inorganic sulfur oxidizing system in green sulfur bacteria. Photosynth Res.

[3] Tang KH, Blankenship RE (2010). Both forward and reverse TCA cycles operate in green sulfur bacteria. J Biol Chem.

[4] Eisen JA, Nelson KE, Paulsen IT, Heidelberg JF, Wu M, Dodson RJ (2002). The complete genome sequence of *Chlorobium tepidum* TLS, a photosynthetic, anaerobic, green-sulfur bacterium. Proc Natl Acad Sci U S A.

[5] Blankenship RE, Matsuura K, Green BR, Parson WW (2003). Antenna complexes from green photosynthetic bacteria. Light-harvesting antennas in photosynthesis. Advances in Photosynthesis and Respiration.

[6] Chen JH, Wu H, Xu C, Liu XC, Huang Z, Chang S (2020). Architecture of the photosynthetic complex from a green sulfur bacterium. Science..

[7] Herbert RA, Tanner AC (1977). The isolation and some characteristics of photosynthetic bacteria (*Chromatiaceae* and *Chlorobiaceae*) from Antarctic marine sediments. J Appl Microbiol.

[8] Burke CM, Burton HR (1988). Photosynthetic bacteria in meromictic lakes and stratified fjords of the Vestfold Hills, Antarctica. Hydrobiologia..

[9] Burke CM, Burton HR (1988). The ecology of photosynthetic bacteria in Burton Lake, Vestfold Hills, Antarctica. Hydrobiologia..

[10] Van Gemerden H, Mas J, Blankenship RE, Madigan MT, Bauer CE (1995). Ecology of phototrophic sulfur bacteria. Anoxygenic photosynthetic bacteria.

[11] Beatty JT, Overmann J, Lince MT, Manske AK, Lang AS, Blankenship RE (2005). An obligately photosynthetic bacterial anaerobe from a deep-sea hydrothermal vent. Proc Natl Acad Sci U S A.

[12] Roeselers G, Norris TB, Castenholz RW, Rysgaard S, Glud RN, Kühl M (2007). Diversity of phototrophic bacteria in microbial mats from Arctic hot springs (Greenland). Environ Microbiol.

[13] Ng C, DeMaere MZ, Williams TJ, Lauro FM, Raftery M, Gibson JAE (2010). Metaproteogenomic analysis of a dominant green sulfur bacterium from Ace Lake, Antarctica. ISME J.

[14] Lauro FM, DeMaere MZ, Yau S, Brown MV, Ng C, Wilkins D (2011). An integrative study of a meromictic lake ecosystem in Antarctica. ISME J.

[15] Comeau AM, Harding T, Galand PE, Vincent WF, Lovejoy C (2012). Vertical distribution of microbial communities in a perennially stratified Arctic lake with saline, anoxic bottom waters. Sci Rep.

[16] Imhoff JF. Biology of green sulfur bacteria. In: eLS. John Wiley & Sons, Ltd. Chichester. 2014. DOI: 10.1002/9780470015902.a0000458.pub2.

[17] Llorens Marès T, Liu Z, Allen LZ, Rusch DB, Craig MT, Dupont CL (2017). Speciation and ecological success in dimly lit waters: horizontal gene transfer in a green sulfur bacteria bloom unveiled by metagenomic assembly. ISME J.

[18] Grouzdev DS, Lunina ON, Gaisin VA, Krutkina MS, Baslerov RV, Savvichev AS (2019). Genome sequences of green- and brown-colored strains of *Chlorobium phaeovibrioides* with gas vesicles. Microbiol Resour Announc.

[19] Panwar P, Allen MA, Williams TJ, Hancock AM, Brazendale S, Bevington J (2020). Influence of the polar light cycle on seasonal dynamics of an Antarctic lake microbial community. Microbiome..

[20] Caumette P (1984). Distribution and characterization of phototrophic bacteria isolated from the water of Bietri Bay (Ebrie Lagoon, Ivory Coast). Can J Microbiol.

[21] Miracle MR, Vicente E (1985). Phytoplankton and photosynthetic sulphur bacteria production in the meromictic coastal lagoon of Cullera (Valencia, Spain). Verhandlungen des Internationalen Verein Limnologie.

[22] Chapin B, Denoyelles F, Gaham DW, Smith VH (2004). A deep maximum of green sulphur bacteria (‘*Chlorochromatium aggregatum*’) in a strongly stratified reservoir. Freshw Biol.

[23] Coolen MJL, Muyzer G, Schouten S, Volkman JK, Damsté JSS, Neretin L (2006). Sulfur and methane cycling during the Holocene in Ace Lake (Antarctica) revealed by lipid and DNA stratigraphy. Past and present water column anoxia. NATO Science Series: IV: Earth and Environmental Sciences.

[24] Frigaard NU, Bryant DA (2004). Seeing green bacteria in a new light: genomics-enabled studies of the photosynthetic apparatus in green sulfur bacteria and filamentous anoxygenic phototrophic bacteria. Arch Microbiol.

[25] Bryant DA, Liu Z, Li T, Zhao F, Costas AMG, Klatt CG, Burnap RL, Vermaas WFJ (2012). Comparative and functional genomics of anoxygenic green bacteria from the taxa Chlorobi, Chloroflexi, and Acidobacteria. Functional genomics and evolution of photosynthetic systems. Advances in Photosynthesis and Respiration.

[26] Liu Z, Klatt CG, Ludwig M, Rusch DB, Jensen SI, Kühl M (2012). ‘*Candidatus* Thermochlorobacter aerophilum’: an aerobic chlorophotoheterotrophic member of the phylum Chlorobi defined by metagenomics and metatranscriptomics. ISME J.

[27] Cavicchioli R (2015). Microbial ecology of Antarctic aquatic systems. Nat Rev Microbiol.

[28] Burch MD (1988). Annual cycle of phytoplankton in Ace Lake, an ice covered, saline meromictic lake. Hydrobiol..

[29] Gibson JAE (1999). The meromictic lakes and stratified marine basins of the Vestfold Hills, East Antarctica. Antarct Sci.

[30] Rankin LM, Gibson JAE, Franzmann PD, Burton HR (1999). The chemical stratification and microbial communities of Ace Lake: a review of the characteristics of a marine derived meromictic lake. Polarforschung..

[31] Gallagher JB, Burton HR (1988). Seasonal mixing of Ellis Fjord, Vestfold Hills, East Antarctica. Estuar Coast Shelf Sci.

[32] Gallagher JB, Burton HR, Calf GE (1989). Meromixis in an Antarctic fjord: a precursor to meromictic lakes on an isostatically rising coastline. Hydrobiologia..

[33] USGS Antarctic Research Atlas. https://lima.usgs.gov/antarctic_research_atlas/ (2007). .

[34] Laybourn-Parry J, Bell EM (2014). Ace Lake: three decades of research on a meromictic, Antarctic lake. Polar Biol.

[35] Grey J, Laybourn-Parry J, Leakey RJG, McMinn A (1997). Temporal patterns of protozooplankton abundance and their food in Ellis Fjord, Princess Elizabeth Land, Eastern Antarctica. Estuar Coast Shelf Sci.

[36] Beaumont KL. Planktonic interactions and particulate flux in Ellis Fjord, East Antarctica. PhD thesis. Hobart: University of Tasmania; 2003.

[37] McMinn A, Bleakley N, Steinburner K, Roberts D, Trenerry L (2000). Effect of permanent sea ice cover and different nutrient regimes on the phytoplankton succession of fjords of the Vestfold Hills Oasis, Eastern Antarctica. J Plankton Res.

[38] DeMaere MZ, Williams TJ, Allen MA, Brown MV, Gibson JAE, Rich J (2013). High level of intergenera gene exchange shapes the evolution of haloarchaea in an isolated Antarctic lake. Proc Natl Acad Sci U S A.

[39] Tschitschko B, Williams TJ, Allen MA, Páez-Espino D, Kyrpides N, Zhong L (2015). Antarctic archaea–virus interactions: metaproteome-led analysis of invasion, evasion and adaptation. ISME J.

[40] Tschitschko B, Williams TJ, Allen MA, Zhong L, Raftery MJ, Cavicchioli R (2016). Ecophysiological distinctions of Haloarchaea from a hypersaline Antarctic lake as determined by metaproteomics. Appl Environ Microbiol.

[41] Tschitschko B, Erdmann S, DeMaere MZ, Roux S, Panwar P, Allen MA (2018). Genomic variation and biogeography of Antarctic haloarchaea. Microbiome..

[42] Imhoff JF (2003). Phylogenetic taxonomy of the family Chlorobiaceae on the basis of 16S rRNA and fmo (Fenna–Matthews–Olson protein) gene sequences. Int J Syst Evol Microbiol.

[43] Masuda N, Nakaya S, Burton HR, Torii T (1988). Trace element distribution in some saline lakes of the Vestfold Hills, Antarctica. Hydrobiologia.

[44] Hogle SL, Thrash JC, Dupont CL, Barbeaua KA (2016). Trace metal acquisition by marine heterotrophic bacterioplankton with contrasting trophic strategies. Appl Environ Microbiol.

[45] Von Ballmoos C, Cook GM, Dimroth P (2008). Unique rotary ATP synthase and its biological diversity. Annu Rev Biophys.

[46] Dibrova DV, Galperin MY, Mulkidjanian AY (2010). Characterization of the N-ATPase, a distinct, laterally transferred Na^+^-translocating form of the bacterial F-type membrane ATPase. Bioinformatics..

[47] Schulz S, Wilkes M, Mills DJ, Kühlbrandt W, Meier T (2017). Molecular architecture of the N-type ATPase rotor ring from *Burkholderia pseudomallei*. EMBO Rep.

[48] Koumandou VL, Kossida S (2014). Evolution of the F_0_F_1_ ATP synthase complex in light of the patchy distribution of different bioenergetic pathways across prokaryotes. PLoS Comput Biol.

[49] Niu Y, Moghimyfiroozabad S, Safaie S, Yang Y, Jonas EA, Alavian KN (2017). Phylogenetic profiling of mitochondrial proteins and integration analysis of bacterial transcription units suggest evolution of F_1_F_0_ ATP synthase from multiple modules. J Mol Evol.

[50] Frigaard NU, Bryant DA, Hell R, Dahl C, Knaff D, Leustek T (2008). Genomic insights into the sulfur metabolism of phototrophic green sulfur bacteria. Sulfur metabolism in phototrophic organisms. Advances in Photosynthesis and Respiration.

[51] Gregersen LH, Bryant DA, Frigaard NU (2011). Mechanisms and evolution of oxidative sulfur metabolism in green sulfur bacteria. Front Microbiol.

[52] Allen MA, Lauro FM, Williams TJ, Burg D, Siddiqui KS, De Francisci D (2009). The genome sequence of the psychrophilic archaeon, *Methanococcoides burtonii*: the role of genome evolution in cold adaptation. ISME J.

[53] Lim J, Thomas T, Cavicchioli R (2000). Low temperature regulated DEAD-box RNA helicase from the Antarctic archaeon, *Methanococcoides burtonii*. J Mol Biol.

[54] Williams TJ, Lauro FM, Ertan H, Burg DW, Poljak A, Raftery MJ (2011). Defining the response of a microorganism to temperatures that span its complete growth temperature range (-2°C to 28°C) using multiplex quantitative proteomics. Environ Microbiol.

[55] Makarova KS, Wolf YI, Iranzo J, Shmakov JA, Alkhnbashi OS, Brouns SJJ (2020). Evolutionary classification of CRISPR–Cas systems: a burst of class 2 and derived variants. Nat Rev Microbiol.

[56] Romine MF, Rodionov DA, Maezato Y, Anderson LN, Nandhikonda P, Rodionova IA (2017). Elucidation of roles for vitamin B12 in regulation of folate, ubiquinone, and methionine metabolism. Proc Natl Acad Sci U S A.

[57] Shelton AN, Seth EC, Mok KC, Han AW, Jackson SN, Haft DR (2019). Uneven distribution of cobamide biosynthesis and dependence in bacteria predicted by comparative genomics. ISME J.

[58] BioCyc (2011). MetaCyc Pathway: adenosylcobalamin biosynthesis accessed between Dec 2020 and Jan 2021.

[59] Karp PD, Billington R, Caspi R, Fulcher CA, Latendresse M, Kothari A, et al. The BioCyc collection of microbial genomes and metabolic pathways. Brief Bioinform. 2017. 10.1093/bib/bbx085. 10.1093/bib/bbx085 PMC6781571 29447345

[60] Hazra AB, Han AW, Mehta AP, Mok KC, Osadchiy V, Begley TP (2015). Anaerobic biosynthesis of the lower ligand of vitamin B12. Proc Natl Acad Sci U S A.

[61] Rodionov DA, Vitreschak AG, Mironov AA, Gelfand MS (2003). Comparative genomics of the vitamin B12 metabolism and regulation in prokaryotes. J Biol Chem.

[62] Rodionov DA, Hebbeln P, Gelfand MS, Eitinger T (2006). Comparative and functional genomic analysis of prokaryotic nickel and cobalt uptake transporters: evidence for a novel group of ATP-binding cassette transporters. J Bacteriol.

[63] Woodson JD, Zayas CL, Escalante-Semerena JC (2003). A new pathway for salvaging the coenzyme B12 precursor cobinamide in archaea requires cobinamide-phosphate synthase (CbiB) enzyme activity. J Bacteriol.

[64] Woodson JD, Escalante-Semerena JC (2004). CbiZ, an amidohydrolase enzyme required for salvaging the coenzyme B12 precursor cobinamide in archaea. Proc Natl Acad Sci U S A.

[65] Gray MJ, Tavares NK, Escalante-Semerena JC (2008). The genome of *Rhodobacter Sphaeroides* strain 2.4.1 encodes functional cobinamide salvaging systems of archaeal and bacterial origins. Mol Microbiol.

[66] Gray MJ, Escalante-Semerena JC (2009). The cobinamide amidohydrolase (cobyric acid-forming) CbiZ enzyme: a critical activity of the cobamide remodeling system of *Rhodobacter sphaeroides*. Mol Microbiol.

[67] Taga ME, Larsen NA, Howard-Jones AR, Walsh CT, Walker GC (2007). BluB cannibalizes flavin to form the lower ligand of vitamin B12. Nature..

[68] Anderson PJ, Lango J, Carkeet C, Britten A, Kräutler B, Hammock BD (2008). One pathway can incorporate either adenine or dimethylbenzimidazole as an α-axial ligand of B12 cofactors in *Salmonella enterica*. J Bacteriol.

[69] Helliwell KA, Lawrence AD, Holzer A, Kudahl UJ, Sasso S, Kräutler B (2016). Cyanobacteria and eukaryotic algae use different chemical variants of Vitamin B12. Curr Biol.

[70] Crouzet J, Cameron B, Cauchois L, Rigault S, Blanche F, Guilhot C (1991). Genetic and sequence analyses of a *Pseudomonas denitrificans* DNA fragment containing two *cob* genes. J Bacteriol.

[71] Debussche L, Couder M, Thibaut D, Cameron B, Crouzet J, Blanche F (1992). Assay, purification, and characterization of cobaltochelatase, a unique complex enzyme catalyzing cobalt insertion in hydrogenobyrinic acid a,c-diamide during coenzyme B12 biosynthesis in *Pseudomonas denitrificans*. J Bacteriol.

[72] Bollivar DW, Suzuki JY, Beatty JT, Dobrowolski JM, Bauer CE (1994). Directed mutational analysis of bacteriochlorophyll A biosynthesis in *Rhodobacter capsulatus*. J Mol Biol.

[73] Petersen BL, Jensen PE, Gibson LC, Stummann BM, Hunter CN, Henningsen KW (1998). Reconstitution of an active magnesium chelatase enzyme complex from the *bchI*, *-D*, and *-H* gene products of the green sulfur bacterium *Chlorobium vibrioforme* in *Escherichia coli*. J Bacteriol.

[74] Willows RD, Al-Karadaghi S, Hansson M, Fodje MN, Hansson A, Olsen JG (2001). Interplay between an AAA module and an integrin I domain may regulate the function of magnesium chelatase. J Mol Biol.

[75] Chew AGM, Frigaard NU, Bryant DA (2009). Mutational analysis of three *bchH* paralogs in (bacterio-)chlorophyll biosynthesis in *Chlorobaculum tepidum*. Photosynth Res.

[76] Johnson ET, Dannert CS (2008). Characterization of three homologs of the large subunit of the magnesium chelatase from *Chlorobaculum tepidum* and interaction with the magnesium protoporphyrin IX methyltransferase. J Biol Chem.

[77] Watanabe F, Katsura H, Takenaka S, Fujita T, Abe K, Tamura Y (1999). Pseudovitamin b12 is the predominant cobamide of an algal health food, spirulina tablets. J Agric Food Chem.

[78] Miyamoto E, Tanioka Y, Nakao T, Barla F, Inui H, Fujita T (2006). Purification and characterization of a corrinoid-compound in an edible cyanobacterium *Aphanizomenon flos-aquae* as a nutritional supplementary food. J Agric Food Chem.

[79] Watanabe F, Miyamoto E, Fujita T, Tanioka Y, Nakano Y (2006). Characterization of a corrinoid compound in the edible (blue-green) alga, Suizenji-nori. Biosci Biotechnol Biochem.

[80] Watanabe F, Tanioka Y, Miyamoto E, Fujita T, Takenaka H, Nakano Y (2007). Purification and characterization of corrinoid-compounds from the dried powder of an edible cyanobacterium, *Nostoc commune* (Ishikurage). J Nutr Sci Vitaminol (Tokyo).

[81] Powell LM, Bowman JP, Skerratt JH, Franzamnn PD, Burton HR (2005). Ecology of a novel *Synechococcus* clade occurring in dense populations in saline Antarctic lakes. Mar Ecol Prog Ser.

[82] Cadieux N, Bradbeer C, Reeger-Schneider E, Köster W, Mohanty AK, Wiener MC (2002). Identification of the periplasmic cobalamin-binding protein BtuF of *Escherichia coli*. J Bacteriol.

[83] Santos JA, Rempel S, Mous STM, Pereira CT, Ter Beek J, De Gier JW (2018). Functional and structural characterization of an ECF-type ABC transporter for vitamin B12. eLife..

[84] Pieńko T, Trylska J (2020). Extracellular loops of BtuB facilitate transport of vitamin B12 through the outer membrane of *E. coli*. PLoS Comput Biol.

[85] Urbanowski ML, Stauffer LT, Plamann LS, Stauffer GV (1987). A new methionine locus, *metR*, that encodes a trans-Acting protein required for activation of *metE* and *metH* in *Escherichia coli* and *Salmonella typhimurium*. J Bacteriol.

[86] Franklund CV, Kadner RJ (1997). Multiple transcribed elements control expression of the *Escherichia coli btuB* gene. J Bacteriol.

[87] Nou X, Kadner RJ (2000). Adenosylcobalamin inhibits ribosome binding to *btuB* RNA. Proc Natl Acad Sci U S A.

[88] Nahvi A, Sudarsan N, Ebert MS, Zou X, Brown KL, Breaker RR (2002). Genetic control by a metabolite binding mRNA. Chem Biol.

[89] Vitreschak AG, Rodionov DA, Mironov AA, Gelfand MS (2003). Regulation of the vitamin B12 metabolism and transport in bacteria by a conserved RNA structural element. RNA..

[90] Borovok I, Gorovitz B, Schreiber R, Aharonowitz Y, Cohen G (2006). Coenzyme B12 controls transcription of the *Streptomyces* Class Ia ribonucleotide reductase *nrdABS* operon *via* a riboswitch mechanism. J Bacteriol.

[91] Barrick JE, Breaker RR (2007). The distributions, mechanisms, and structures of metabolite-binding riboswitches. Genome Biol.

[92] Warner DF, Savvi S, Mizrahi V, Dawes SS (2007). A riboswitch regulates expression of the coenzyme B12-independent methionine synthase in *Mycobacterium tuberculosis*: Implications for differential methionine synthase function in strains H37Rv and CDC1551. J Bacteriol.

[93] Li J, Ge Y, Zadeh M, Curtiss R, Mohamadzadeh M (2020). Regulating vitamin B12 biosynthesis *via* the *cbiMCbl* riboswitch in *Propionibacterium* strain UF1. Proc Natl Acad Sci U S A.

[94] Dy RL, Przybilski R, Semeijn K, Salmond GPC, Fineran PC (2014). A widespread bacteriophage abortive infection system functions through a Type IV toxin–antitoxin mechanism. Nucleic Acids Res.

[95] Elena SF, Agudelo-Romero P, Lalić J (2009). The evolution of viruses in multi-host fitness landscapes. Open Virol J.

[96] Zborowskya S, Lindell D (2019). Resistance in marine cyanobacteria differs against specialist and generalist cyanophages. Proc Natl Acad Sci U S A.

[97] Zhao L, Duffy S (2019). Gauging genetic diversity of generalists: a test of genetic and ecological generalism with RNA virus experimental evolution. Virus Evol.

[98] Bowman JP, McCammon SA, Rea SM, McMeekin TA (2000). The microbial composition of three limnologically disparate hypersaline Antarctic lakes. FEMS Microbiol Lett.

[99] Yau S, Lauro FM, Williams TJ, DeMaere MZ, Brown MV, Rich J (2013). Metagenomic insights into strategies of carbon conservation and unusual sulfur biogeochemistry in a hypersaline Antarctic lake. ISME J.

[100] Sato K, Ishida K, Kuno T, Mizuno A, Shimizu S (1981). Regulation of vitamin B12 and bacteriochlorophyll biosynthesis in a facultative methylotroph, *Protaminobacter ruber*. J Nutr Sci Vitaminol (Tokyo).

[101] Fuhrmann S, Overmann J, Pfennig N, Fischer U (1993). Influence of vitamin B12 and light on the formation of chlorosomes in green- and brown-colored *Chlorobium* species. Arch Microbiol.

[102] Berg M, Goudeau D, Olmsted C, McMahon KD, Thweatt J, Bryant D, et al. Host population diversity as a driver of viral infection cycle in wild populations of green sulfur bacteria with long standing virus-host interactions. ISME J. 2021. 10.1038/s41396-020-00870-1. 10.1038/s41396-020-00870-1 PMC8163819 33452481

[103] Cary SC, McDonald IR, Barrett JE, Cowan DA (2010). On the rocks: the microbiology of Antarctic Dry Valley soils. Nat Rev Microbiol.

[104] Rintoul SR, Chown SL, DeConto RM, England MH, Fricker HA, Masson-Delmotte V (2018). Choosing the future of Antarctica. Nature..

[105] Ji M, Greening C, Vanwonterghem I, Carere CR, Bay SK, Steen JA (2017). Atmospheric trace gases support primary production in Antarctic desert surface soil. Nature..

[106] Williams TJ, Allen MA, DeMaere MZ, Kyrpides NC, Tringe SG, Woyke T (2014). Microbial ecology of an Antarctic hypersaline lake: genomic assessment of ecophysiology among dominant haloarchaea. ISME J.

[107] Cavicchioli R, Ripple WJ, Timmis KN, Azam F, Bakken LR, Baylis M (2019). Scientists’ warning to humanity: microorganisms and climate change. Nat Rev Microbiol.

[108] Williams TJ, Allen MA, Ivanova N, Huntemann M, Haque S, Hancock AM (2021). Genome analysis of a verrucomicrobial endosymbiont with a tiny genome discovered in an Antarctic lake. Front Microbiol.

[109] Li H (2015). BFC: correcting Illumina sequencing errors. Bioinformatics..

[110] Nurk S, Bankevich A, Antipov D, Gurevich AA, Korobeynikov A, Lapidus A (2013). Assembling single-cell genomes and mini-metagenomes from chimeric MDA products. J Comput Biol.

[111] Nurk S, Meleshko D, Korobeynikov A, Pevzner PA (2017). MetaSPAdes: a new versatile metagenomic assembler. Genome Res.

[112] Bowers RM, Kyrpides NC, Stepanauskas R, Smith MH, Doud D, Reddy TBK (2017). Minimum information about a single amplified genome (MISAG) and a metagenome-assembled genome (MIMAG) of bacteria and archaea. Nat Biotechnol.

[113] Gurevich A, Saveliev V, Vyahhi N, Tesler G (2013). QUAST: quality assessment tool for genome assemblies. Bioinformatics..

[114] Darling ACE, Mau B, Blattner FR, Perna NT (2004). Mauve: multiple alignment of conserved genomic sequence with rearrangements. Genome Res.

[115] Camacho C, Coulouris G, Avagyan V, Ma N, Papadopoulos J, Bealer K (2009). BLAST+: architecture and applications. BMC Bioinformatics.

[116] JGI BBMap Guide. https://jgi.doe.gov/data-and-tools/bbtools/bb-tools-user-guide/bbmap-guide/ (2014). Accessed in Oct 2020.

[117] Li H, Handsaker B, Wysoker A, Fennell T, Ruan J, Homer N (2009). The sequence alignment/map (SAM) format and SAMtools. Bioinformatics..

[118] Robinson JT, Thorvaldsdóttir H, Winckler W, Guttman M, Lander ES, Getz G (2011). Integrative Genomics Viewer. Nat Biotechnol.

[119] ExPASy BLAST (1993). UniProtKB/Swiss-Prot database accessed between Oct 2020 and May 2021.

[120] NCBI BLAST (1994). UniProtKB and RefSeq databases accessed between Oct 2020 and May 2021.

[121] Love MI, Huber W, Anders S (2014). Moderated estimation of fold change and dispersion for RNA-seq data with DESeq2. Genome Biol.

[122] Kalvari I, Nawrocki EP, Argasinska J, Olvera NQ, Finn RD, Bateman A (2018). Non-coding RNA analysis using the Rfam database. Curr Protoc Bioinformatics.

[123] Kalvari I, Nawrocki EP, Palacios NQ, Argasinska J, Lamkiewicz K, Marz M (2021). Rfam 14: expanded coverage of metagenomic, viral and microRNA families. Nucleic Acids Res.

[124] Pritchard L, Glover RH, Humphris S, Elphinstone JG, Toth IK (2016). Genomics and taxonomy in diagnostics for food security: soft-rotting enterobacterial plant pathogens. Anal Methods.

[125] AAI-profiler: fast proteome-wide search reveals taxonomic outliers. http://ekhidna2.biocenter.helsinki.fi/AAI/ (2018). Accessed between September and November 2020.

[126] Medlar AJ, Toronen P, Holm L (2018). AAI-profiler: fast proteome-wide exploratory analysis reveals taxonomic identity, misclassification and contamination. Nucleic Acids Res.

[127] Kumar S, Stecher G, Li M, Knyaz C, Tamura K (2018). MEGA X: molecular evolutionary genetics analysis across computing platforms. Mol Biol Evol.

[128] Paez-Espino D, Roux S, Chen IA, Palaniappan K, Ratner A, Chu K (2019). IMG/VR v.2.0: an integrated data management and analysis system for cultivated and environmental viral genomes. Nucleic Acids Res.

[129] Paez-Espino D, Eloe-Fadrosh EA, Pavlopoulos GA, Thomas AD, Huntemann M, Mikhailova N (2016). Uncovering Earth’s virome. Nature..

