# Supplementary material for: Remarkably coherent population structure for a dominant Antarctic Chlorobium species
Source: Microbiome. 2021 Nov 26;9:231. doi: 10.1186/s40168-021-01173-z (PMC8620254; doi:10.1186/s40168-021-01173-z)
Supplement: Supplementary file 2 — Additional file 1. Data on Ca. Chlorobium antarcticum population structure, genomic variation, and viral analysis. Supplementary text, figures, and tables. [file 40168_2021_1173_MOESM2_ESM.pdf]

Remarkably coherent population structure for a dominant Antarctic *Chlorobium* species

Pratibha Panwar, Michelle A. Allen, Timothy J. Williams, Sabrina Haque, Sarah Brazendale, Alyce M. Hancock, David Paez-Espino and Ricardo Cavicchioli

Additional file 1: Supplemental data and findings about *Ca.* Chlorobium antarcticum

Supplementary text
***Ca.* Chlorobium antarcticum population structure in AL, EF and TB**

Supplementary figures
Fig. S1 Comparison of marker genes from *Ca.* Chlorobium antarcticum and Cpv-DSM265.
Fig. S2 GC content and read depth plot.
Fig. S3 Schematic of cobalamin biosynthesis and cobinamide salvaging pathways.
Fig. S4 Comparison of the functional potential of *Ca.* Chlorobium antarcticum and Cpv-DSM265.

Supplementary tables
Table S1 Antarctic metagenomes analysed.
Table S2 *Ca.* Chlorobium antarcticum MAGs from AL, EF and TB.
Table S3 *Ca.* Chlorobium antarcticum AL_ref MAG and EF_ref MAG contigs.
Table S4 *Ca.* Chlorobium antarcticum LCR gene autoannotations.
Table S5 Grouping of AL *Ca*. Chlorobium antarcticum LCR genes associated with transport.
Table S6 AL *Ca*. Chlorobium antarcticum LCR genes associated with cobalamin biosynthesis and cobinamide and pseudocobalamin salvaging.
Table S7 EF_ref MAG genes with SNPs in AL and TB metagenomes.
Table S8 CRISPR-Cas defence system genes in some members of Chlorobiaceae family.
Table S9 *Ca*. Chlorobium antarcticum defence genes.
Table S10 Spacer and repeat sequences in AL, EF and TB *Ca*. Chlorobium antarcticum MAGs.
Table S11 Viral contigs with matches to *Ca*. Chlorobium antarcticum spacers.
Table S12 Host analysis of viral clusters and singletons with matches to TB and EF *Ca*. Chlorobium antarcticum spacers.
Table S13 Description of *Ca.* Chlorobium antarcticum metabolic capacity and metadata.
Table S14 AL, EF and TB metagenomes used for FR analyses of *Ca*. Chlorobium antarcticum MAGs.
Table S15 Marker genes of Chlorobiaceae family members used for the phylogenetic analysis of *Ca*. Chlorobium antarcticum.

References

***Ca.* Chlorobium antarcticum population structure in AL, EF and TB**

The GSB community in the three systems (AL, EF and TB) was very similar to each other with *Ca.* Chlorobium antarcticum essentially the only species, exhibiting relatively subtle differences in genomic variation and population structure within AL and between the three systems. Differences were observed with regards to the contribution of LCRs (most contribution to TB) and SNPs (most similar between EF and TB). We considered what could cause differences in population structure for LCRs or SNPs, and what could cause differences between the LCR and SNP population structures.

(i) *Biomass collection and data processing*. Biomass for AL and EF was collected on large format, sequential, size-fractionated filters, whereas TB biomass was collected in Sterivex cartridges (see the "Methods" section); while the quantity of biomass collected was higher on the large format filters, the captured size range was similar: 0.1–20 µm and 0.22–20 µm, respectively. DNA was extracted from the two types of filters using similar methods. All metagenome data contained paired-end reads sequenced using Illumina technology which produced ≥13 million reads (containing ≥3 billion bases) per metagenome. The calculated relative abundances represent normalized data, so size of datasets cannot affect abundance calculations. We conclude that the sampling, and generation and treatment of data cannot account for LCR or SNP differences.

(ii) *Environment.* AL is a limnological system that was isolated from the ocean ~5,000 year ago, whereas EF and TB are fjords with marine basins that are still connected to the Southern Ocean (albeit across shallow sills). Intuitively, one may expect the two fjords to harbour more similar population structures, but this is only apparent for SNPs. The depths of the oxic-anoxic interfaces (TB, ~11 m; AL, ~14 m; EF, ~45 m) and expected attenuation of PAR with depth, also offers no obvious explanations for the abundance trends of SNPs and LCRs between the systems. Future efforts to obtain physicochemical data for EF and TB will be required in order to perform analyses to assign specific factors as explanatory variables of trends in population variation data (LCRs and SNPs).

(iii) *Biological processes.* Among the SNPs identified were non-synonymous mutations in DNA/RNA modification genes: DNA-directed RNA polymerase subunit beta' (*rpoB*) [F→S], recombination protein (*recA*) [V→A], excinuclease ABC subunit A (*uvrA*) [D→N], excinuclease ABC subunit B (*uvrB*) [M→V; V→I], ribonuclease Y [C→R; R→C; A→T], and RNA helicase *deaD* [S→L] (Additional file 1: Table S7). Among the LCRs, two genes have potential functions in DNA repair: ATP-dependent helicase/nuclease (*addA*) and DNA repair protein (*radC*) (Additional file 1: Table S4). It is possible that genetic variation in some of these genes contributes to the differences in the extent and type of SNP and/or LCR population variation that was observed between the populations of *Ca.* Chlorobium antarcticum in AL, EF and TB.

(a)

```
Cpv-DSM265                      -TACAACGGA… GAAGCAGCTT CGGCTGTGAG… AGAAATCGAG GACAATACCA…
AL Ca. Chlorobium antarcticum   CTACAACGGA… AAAGCAGCTT CGGTTGTGAG… AGAAATCGGG GATAATACCA…
EF Ca. Chlorobium antarcticum   CTACAACGGA… AAAGCAGCTT CGGTTGTGAG… AGAAATCGGG GATAATACCA…
TB Ca. Chlorobium antarcticum   CTACAACGGA… AAAGCAGCTT CGGTTGTGAG… AGAAATCGGG GATAATACCA…
                                1           71            84                  159    163

CGGGACCGCA TGGTCATGTT GTTAAA-GAT… AGGATGAACC… CCGGCACTGA… TTGCGACTAA ACCTCTTGAA…
CGAAACCGCA TGGTTTTGCT GTTAAATGAT… AGGATGAGCC… CCGACACTGA… TGGCGACTAA TCCTCCTGAA…
CGAAACCGCA TGGTTTTGCT GTTAAATGAT… AGGATGAGCC… CCGACACTGA… TGGCGACTAA TCCTCCTGAA…
CGAAACCGCA TGGTTTTGCT GTTAAATGAT… AGGATGAGCC… CCGACACTGA… TGGCGACTAA TCCTCCTGAA…
  184,         195, 199      207          228        464         982       991    996
  185          196

TCGTAACAGG… TAACAGGTCA… GTGAATGTGT… CTTTTAAT
TCGTCACAGG… TAACAGGTTA… GTGAATATGT… CTTTT---
TCGTCACAGG… TAACAGGTTA… GTGAATATGT… CTTTT---
TCGTCACAGG… TAACAGGTTA… GTGAATATGT… CTTTT---
   1025             1119        1357         1505
```

(b)

```
Cpv-DSM265                      MALFGTKDAT TAHSDYEIVL EGGASSWGKV KARAKVNVPP ALPLLPADCN
AL Ca. Chlorobium antarcticum   MALFGTKDAT TAHSDYEIVL EGGASSWGKV KARAKVNVPP ALPLLPADCN
EF Ca. Chlorobium antarcticum   MALFGTKDAT TAHSDYEIVL EGGASSWGKV KARAKVNVPP ALPLLPADCN
TB Ca. Chlorobium antarcticum   MALFGTKDAT TAHSDYEIVL EGGASSWGKV KARAKVNVPP ALPLLPADCN

51  VKINVKPLDP AKGFVRFSAV IESIVDSTKN KLVVEADIAN ETSERRICVG EGSVTVGDFS HSFSFEGSVV
    VKINVKPLDP AKGFVRFSAV IESIVDSTKN KLVVEADIAN ETNERRICVG EGSVTVGDFS HSFSFEGSVV
    VKINVKPLDP AKGFVRFSAV IESIVDSTKN KLVVEADIAN ETNERRICVG EGSVTVGDFS HSFSFEGSVV
    VKINVKPLDP AKGFVRFSAV IESIVDSTKN KLVVEADIAN ETNERRICVG EGSVTVGDFS HSFSFEGSVV

121 NLFYYRSDAV KRNVPNPIYM QGRQFHDIIM KVPLDNPDVI DTWEGTMRSM QTTGAFNDWI REFWFIGPAF
    NLFYYRSDAV KRNVPNPIYM QGRQFHDIIM KVPLDNPDVI DTWEGTMRSL QTTGAFNDWI REFWFIGPAF
    NLFYYRSDAV KRNVPNPIYM QGRQFHDIIM KVPLDNPDVI DTWEGTMRSL QTTGAFNDWI REFWFIGPAF
    NLFYYRSDAV KRNVPNPIYM QGRQFHDIIM KVPLDNPDVI DTWEGTMRSL QTTGAFNDWI REFWFIGPAF

191 TALNEGGQRI SKVEVNSIGT QSGEKGPVGV TRWRFSHGGS GIVDSIARWA ELFPADKLNR PASVEAAFRS
    TALNEGGQRI SKIEVNSIGT QSGEKGPEGV TRWRFSHGGS GIVDSIARWA ELFPADKLNR PASVEAAFRS
    TALNEGGQRI SKIEVNSIGT QSGEKGPEGV TRWRFSHGGS GIVDSIARWA ELFPADKLNR PASVEAAFRS
    TALNEGGQRI SKIEVNSIGT QSGEKGPEGV TRWRFSHGGS GIVDSIARWA ELFPADKLNR PASVEAAFRS

261 DSQGIEVKVD GDFPGVSVDA GGGLRRILNH PLIPLVHHGM VGKFNDFTVD TQLKVVLPKG YKIRYAAPQF
    DSQGIEVKVD GDFPGVSVDA GGGLRRILNH PLIPLVHHGM VGKFNDFTVD TQLKIVLPKG YKVRYAAPQF
    DSQGIEVKVD GDFPGVSVDA GGGLRRILNH PLIPLVHHGM VGKFNDFTVD TQLKIVLPKG YKVRYAAPQF
    DSQGIEVKVD GDFPGVSVDA GGGLRRILNH PLIPLVHHGM VGKFNDFTVD TQLKIVLPKG YKVRYAAPQF

331 RSQNLEEYRW SGGAYARWVE HVCKGGTGQF EVLYAQ
    RSQNLEEYRW SGGAYARWVE HVCKGGTGQF EVLYAQ
    RSQNLEEYRW SGGAYARWVE HVCKGGTGQF EVLYAQ
    RSQNLEEYRW SGGAYARWVE HVCKGGTGQF EVLYAQ
```

Fig. S1 Comparison of marker genes from *Ca.* Chlorobium antarcticum and Cpv-DSM265. 16S rRNA gene sequences **(a)** and FmoA protein sequences **(b)** from AL, EF and TB *Ca.* Chlorobium antarcticum, and Cpv-DSM265. Locations of mismatch between Cpv-DSM265 (magenta) and conservation between *Ca.* Chlorobium antarcticum sequences (green). **(a)** The complete 16S rRNA gene sequences were 1,505–1,506 bp long and only the regions with sequence variation are shown. Regions of discontinuous sequence (dotted regions within the nucleotide sequence), sequence position (numbers below sequences), and gaps at the ends of sequences and position 207 (dashes replacing nucleotides) are shown. **(b)** The complete FmoA protein sequences (366 amino acid residues long) and position within the sequence (numbers to the left of the sequences) are shown.

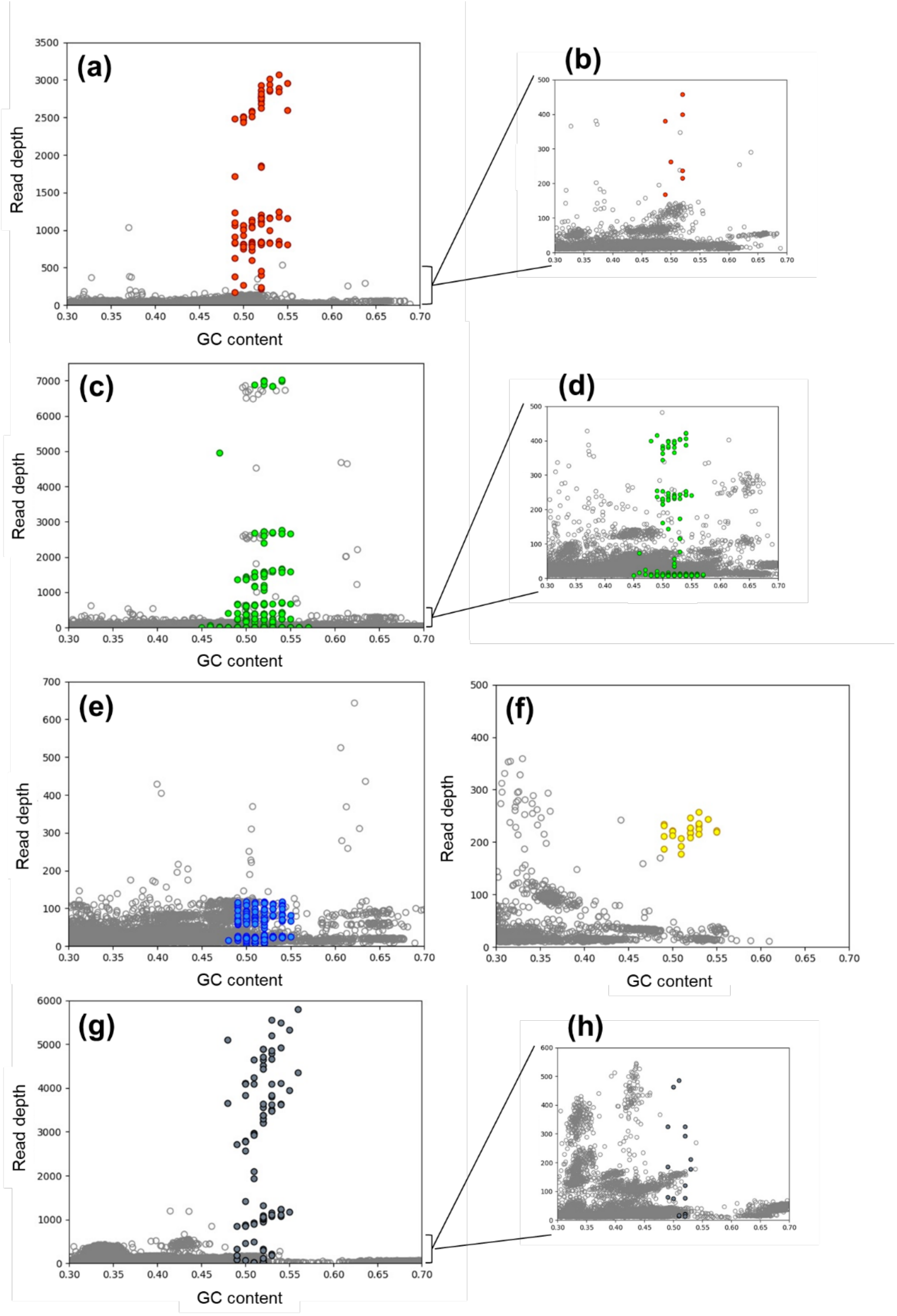

Fig. S2 GC content and read depth plot. *Ca.* Chlorobium antarcticum MAG contigs from AL summer (**a** and **b**, ; Dec 2014), AL spring (**c** and **d**, ; Nov 2008 and 2013), AL winter (**e**, ; Jul and Aug 2014), TB (**f**,) and EF (**g** and **h**,) were plotted in a GC-read depth 2D space, along with the oxic-anoxic interface metagenome contigs ≥10 kb length () from the respective seasons and systems. Panels **b**, **d** and **h** show enhanced-view sections of plots to highlight specific metagenome contig clusters. The Oct 2014 metagenome data were not incorporated because the abundance of *Ca.* Chlorobium antarcticum was low (<1%; mean read depth, 26).

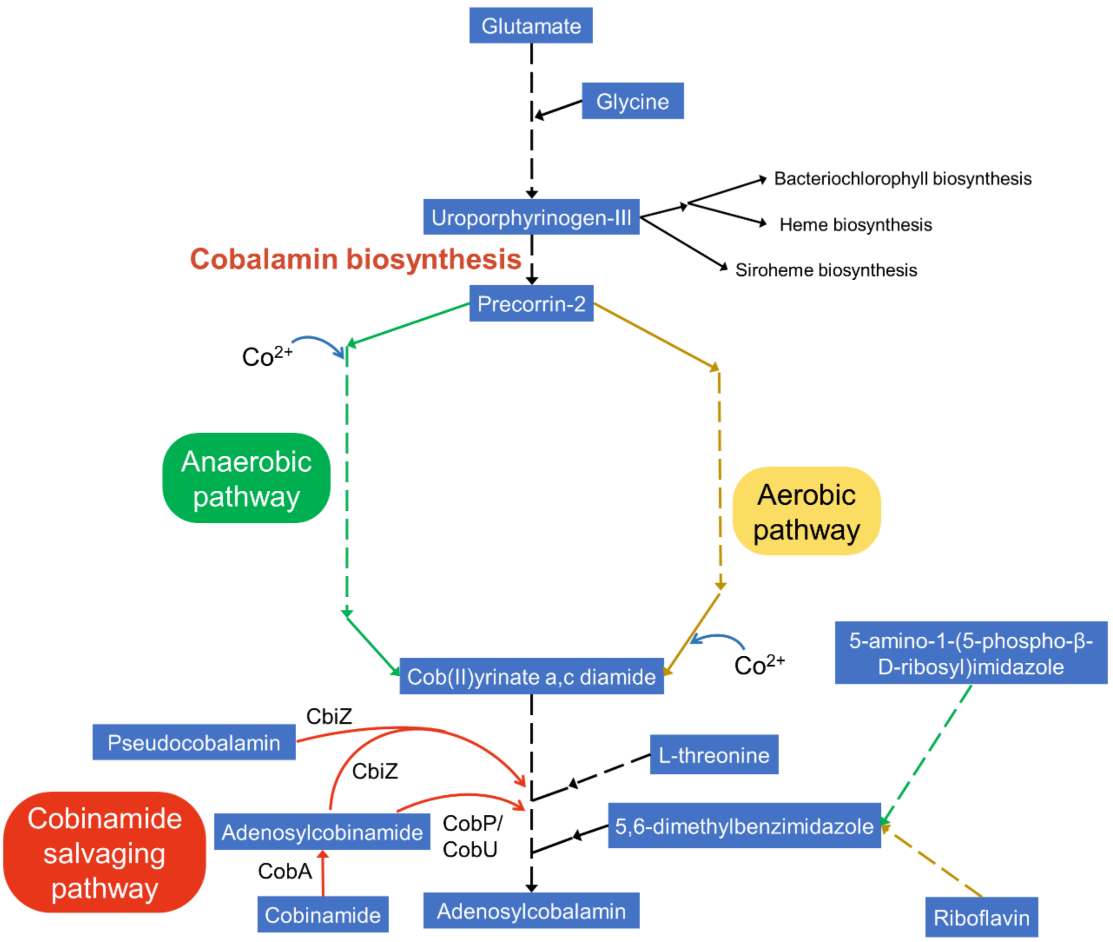

Fig. S3 Schematic of cobalamin biosynthesis and cobinamide salvaging pathways. The starting substrate for cobalamin biosynthesis is glutamate, whereas it is glycine for bacteriochlorophyll and heme biosynthesis. The three biosynthesis pathways separate after formation of uroporphyrinogen-III. The aerobic (yellow branch) and anaerobic (green branch) pathways of cobalamin biosynthesis share common reactions before the formation of precorrin-2, and after the formation of cob(II)yrinate a,c-diamide. The two pathways differ in the timing of cobalt insertion into the corrin ring and the source of 5,6-dimethylbenzimidazole, the lower axial ligand of adenosylcobalamin. The cobinamide salvaging pathway (red branch) involves the conversion of cobinamide to intermediates of the cobalamin biosynthesis pathway using CobP/CobU and/or CbiZ. Of these two enzymes, CbiZ is capable of remodelling pseudocobalamin into intermediates of the cobalamin biosynthesis pathway [1]. The dashed arrows connecting the intermediate substrates indicate multi-step processes. Pathway information was derived from BioCyc online service [2, 3] and published data [1, 4–10]. CobA, corrinoid adenosyltransferase; CobP/CobU, adenosylcobinamide kinase/adenosylcobinamide-phosphate guanylyltransferase; CbiZ, adenosylcobinamide amidohydrolase.

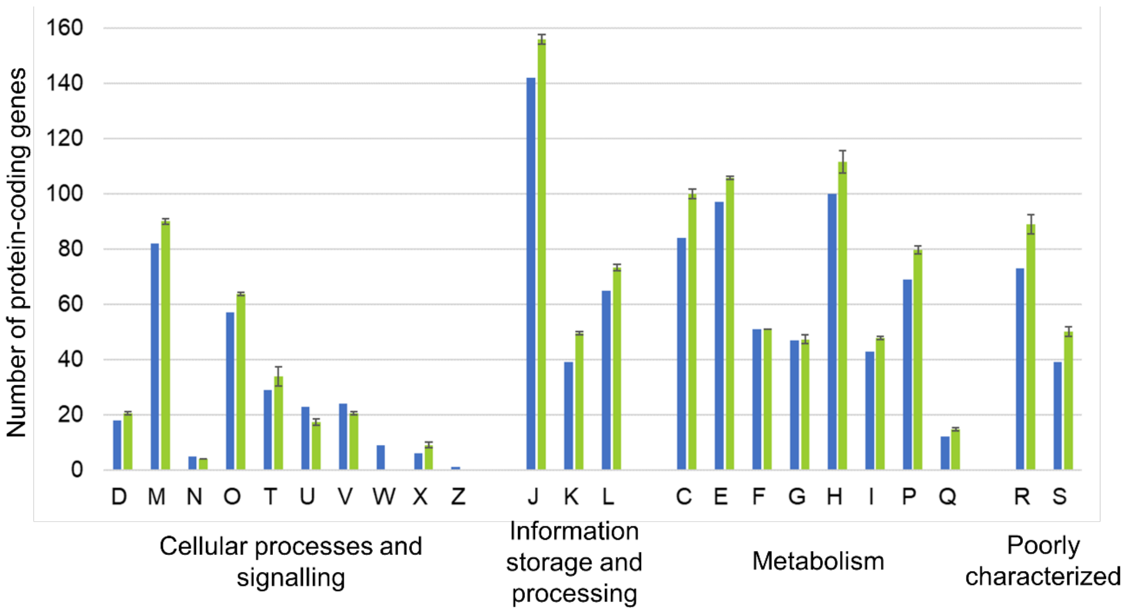

Fig. S4 Comparison of the functional potential of *Ca.* Chlorobium antarcticum and Cpv-DSM265. The bar chart shows the number of protein-coding genes (y-axis) from *Ca.* Chlorobium antarcticum (■) and Cpv-DSM265 (■) that were classified into COG categories (x-axis). The data for *Ca.* Chlorobium antarcticum was calculated from the COG categorisation of protein-coding genes in AL (AL_ref MAG), EF (EF_ref MAG) and the TB MAG from the 11 m depth metagenome (Additional file 1: Table S2). The green bars represent the average number of *Ca.* Chlorobium antarcticum genes from the three Vestfold Hills systems that were classified to each COG category, and the error bars indicate the standard deviation. The COG categories do not include categories 'Y', 'A', and 'B', as none of the protein-coding genes from *Ca.* Chlorobium antarcticum or Cpv-DSM265 were classified in these categories. COG categories: A, RNA processing and modification; B, Chromatin structure and dynamics; C, Energy production and conversion; D, Cell cycle control, cell division, chromosome partitioning; E, Amino acid transport and metabolism; F, Nucleotide transport and metabolism; G, Carbohydrate transport and metabolism; H, Coenzyme transport and metabolism; I, Lipid transport and metabolism; J, Translation, ribosomal structure and biogenesis; K, Transcription; L, Replication, recombination and repair; M, Cell wall/membrane/envelope biogenesis; N, Cell motility; O, Posttranslational modification, protein turnover, chaperones; P, Inorganic ion transport and metabolism; Q, Secondary metabolites biosynthesis, transport and catabolism; R, General function prediction only; S, Function unknown; T, Signal transduction mechanisms; U, Intracellular trafficking, secretion, and vesicular transport; V, Defence mechanisms; W, Extracellular structures; X, Mobilome: prophages, transposons; Y, Nuclear structure; Z, Cytoskeleton.

Table S1 Antarctic metagenomes analysed.

Sample collection date (DDMMYYYY); Depth (m); Filter fraction (µm)	IMG Genome IDs	Metagenome filtered reads (bp)	Assembled metagenome size (bp)[A]	Total protein-coding genes
**Ace Lake**				
20/12/2006; 12.7 m; 3 µm	3300028203	83,214,739	10,703,483	20,757
20/12/2006; 12.7 m; 0.8 µm	3300028201	208,538,507	11,925,309	23,740
20/12/2006; 12.7 m; 0.1 µm	3300028204	240,290,391	6,971,450	13,087
20/12/2006; 14 m; 0.8 µm	3300028302	165,208,287	27,504,336	56,468
20/12/2006; 18 m; 0.8 µm	3300028227	214,177,665	34,270,862	71,009
21/11/2008; 12.8 m; 3 µm	3300025433	7,377,945,147	191,332,554	330,516
21/11/2008; 12.8 m; 0.8 µm	3300025380	7,969,400,898	118,925,863	224,047
21/11/2008; 12.8 m; 0.1 µm	3300025362	15,030,492,867	90,472,821	190,960
21/11/2008; 18 m; 0.8 µm	3300025586	10,550,636,481	338,938,472	589,716
23/11/2008; 23 m; 3 µm	3300025698	8,926,498,848	428,043,704	894,948
23/11/2008; 23 m; 0.8 µm	3300025661	8,835,913,368	414,688,901	822,281
26/11/2013; 13.5 m; 3 µm	3300022882	4,632,992,773	197,528,912	370,963
26/11/2013; 13.5 m; 0.8 µm	3300023244	4,017,414,066	152,968,368	281,280
26/11/2013; 13.5 m; 0.1 µm	3300022871	4,289,343,500	153,918,125	304,781
26/11/2013; 15 m; 0.1 µm	3300023435	3,982,384,098	204,889,614	458,784
26/11/2013; 19 m; 0.8 µm	3300023262	5,356,530,473	256,708,329	493,455
27/11/2013; 24 m; 0.8 µm	3300022887	4,489,480,975	197,136,157	423,504
3/07/2014; 13.5 m; 3 µm	3300022834	3,025,335,676	150,334,734	279,053
3/07/2014; 13.5 m; 0.8 µm	3300023241	3,917,460,255	176,108,874	316,827
3/07/2014; 13.5 m; 0.1 µm	3300023257	4,754,144,028	246,566,898	516,984
21/08/2014; 14.5 m; 3 µm	3300022864	4,208,293,249	203,541,480	379,585
21/08/2014; 14.5 m; 0.8 µm	3300024048	4,438,778,032	185,710,747	327,952
21/08/2014; 14.5 m; 0.1 µm	3300022890	3,761,803,592	196,439,047	427,804
21/10/2014; 13 m; 3 µm	3300022856	3,793,702,914	185,885,369	366,842
21/10/2014; 13 m; 0.8 µm	3300022859	3,615,901,126	148,572,713	281,988
21/10/2014; 13 m; 0.1 µm	3300022821	3,169,765,298	119,795,036	247,086
21/10/2014; 16 m; 0.8 µm	3300023249	3,472,734,434	161,447,324	294,441
4/12/2014; 12 m; 0.1 µm	3300022851	3,582,064,538	119,299,278	248,470
4/12/2014; 13.4 m; 3 µm	3300031697	14,149,086,706	400,324,806	718,959
4/12/2014; 13.4 m; 0.8 µm	3300022826	2,989,229,242	78,299,135	145,800
4/12/2014; 13.4 m; 0.1 µm	3300023292	3,878,932,484	85,111,111	181,733
4/12/2014; 14 m; 3 µm	3300023253	3,420,681,173	167,955,693	307,470
4/12/2014; 14 m; 0.8 µm	3300023233	3,250,064,514	144,877,168	252,928
3/12/2014; 19 m; 3 µm	3300022860	4,079,964,767	181,977,179	369,802
3/12/2014; 19 m; 0.8 µm	3300022846	3,983,828,178	165,102,958	309,999
3/12/2014; 19 m; 0.1 µm	3300023061	3,209,269,596	152,256,002	384,107
3/12/2014; 24 m; 0.8 µm	3300023299	5,006,350,890	217,304,898	440,798
**Ellis Fjord**				
9/10/2014; 5 m; 3 µm	3300031658	22,583,676,006	512,812,684	1,103,847
9/10/2014; 5 m; 0.8 µm	3300031629	17,208,422,590	806,100,132	1,620,667
9/10/2014; 5 m; 0.1 µm	3300031659	22,404,979,271	880,035,495	1,841,661
9/10/2014; 45 m; 3 µm	3300031631	16,642,938,015	430,745,788	939,013
9/10/2014; 45 m; 0.8 µm	3300031741	18,009,360,230	575,028,230	1,095,421
9/10/2014; 45 m; 0.1 µm	3300031603	13,346,656,379	637,704,446	1,320,380
8/10/2014; 60 m; 3 µm	3300031645	19,804,223,504	908,736,180	1,987,014
8/10/2014; 60 m; 0.8 µm	3300031657	15,894,964,153	433,198,916	809,885
8/10/2014; 60 m; 0.1 µm	3300031601	17,233,115,763	295,045,072	509,553

2/11/2014; 18 m; 3 µm	3300031602	18,753,292,708	211,651,588	361,601
2/11/2014; 18 m; 0.8 µm	3300031660	15,932,943,833	734,660,849	1,350,693
2/11/2014; 18 m; 0.1 µm	3300031696	16,951,600,293	508,273,244	909,436
**Taynaya Bay**				
28/11/2014; 5 m; 0.22 µm	3300038912	11,415,818,068	256,201,821	474,896
28/11/2014; 11 m; 0.22 µm	3300039187	12,286,944,772	439,736,431	928,219

[A] Assembled metagenome size in the table refers to the total length of all contigs assembled from a metagenome. Metagenomes used for analysing *Ca.* Chlorobium antarcticum genomic variation (orange font), and calculating *Chlorobium* OTU abundances, 16S rRNA SNP analysis and/or viral analyses (all metagenomes). Filter fractions: 3, 3–20 µm; 0.8, 0.8–3 µm; 0.1, 0.1–0.8 µm; 0.22, 0.22–20 µm.

Table S2 *Ca.* Chlorobium antarcticum MAGs from AL, EF and TB.

Metagenome [sample collection date (DDMMYYYY); depth (m); filter fraction (µm)]	IMG Bin IDs[A]	Bin completeness (%)[B]	Total base pair count (bp)	Gene count	Scaffold count
**Ace Lake**					
20/12/2006; 12.7 m; 3 µm*	3300028203_1	98	1,799,622	1968	32
20/12/2006; 12.7 m; 0.8 µm*	3300028201_1	98	1,846,253	1956	17
20/12/2006; 14 m; 0.8 µm	3300028302_2	95	1,719,822	2066	58
20/12/2006; 18 m; 0.8 µm	3300028227_2	68	1,219,845	1799	195
21/11/2008; 12.8 m; 3 µm*	3300025433_15	87	1,561,142	1554	27
21/11/2008; 12.8 m; 0.8 µm*	3300025380_8	60	915,115	905	13
21/11/2008; 12.8 m; 0.1 µm*	3300025362_8	66	1,027,280	1021	10
21/11/2008; 14.1 m; 3 µm	3300025649_20	99	1,717,607	1718	37
21/11/2008; 14.1 m; 0.8 µm	3300025628_24	72	1,339,744	1315	21
21/11/2008; 14.1 m; 0.1 µm	3300025697_16	54	946,925	1090	184
21/11/2008; 18 m; 3 µm	3300025642_35	64	1,147,570	1132	20
21/11/2008; 18 m; 0.8 µm	3300025586_24	99	1,760,585	1740	23
21/11/2008; 18 m; 0.1 µm	3300025669_14	99	1,772,585	1750	20
23/11/2008; 23 m; 3 µm	3300025698_17	99	1,750,727	1739	22
23/11/2008; 23 m; 0.8 µm	3300025661_20	72	1,347,288	1345	27
26/11/2013; 13.5 m; 3 µm*	3300022882_7	99	1,780,829	1765	28
26/11/2013; 13.5 m; 0.8 µm*	3300023244_8	99	1,784,037	1767	28
26/11/2013; 13.5 m; 0.1 µm*	3300022871_5	99	1,792,085	1781	27
26/11/2013; 15 m; 3 µm	3300023234_7	97	1,681,376	1733	109
26/11/2013; 15 m; 0.8 µm	3300022854_6	99	1,793,372	1777	23
26/11/2013; 15 m; 0.1 µm	3300023435_5	99	1,746,873	1742	27
26/11/2013; 19 m; 0.8 µm	3300023262_7	99	1,741,261	1742	34
27/11/2013; 24 m; 0.8 µm	3300022887_7	97	1,660,438	1732	141
27/11/2013; 24 m; 0.1 µm	3300031227_17	95	1,650,130	1765	134
3/07/2014; 13.5 m; 3 µm*	3300022834_6	99	1,745,898	1735	26
3/07/2014; 13.5 m; 0.8 µm*	3300023241_6	99	1,784,741	1776	30
3/07/2014; 13.5 m; 0.1 µm*	3300023257_7	99	1,789,942	1779	23
21/08/2014; 14.5 m; 3 µm*	3300022864_8	99	1,748,321	1738	23
21/08/2014; 14.5 m; 0.8 µm*	3300024048_8	99	1,784,669	1772	24
21/08/2014; 14.5 m; 0.1 µm*	3300022890_5	99	1,753,999	1747	19
21/10/2014; 13 m; 0.8 µm*	3300022859_8	92	1,620,807	1696	120
21/10/2014; 13 m; 0.1 µm*	3300022821_10	67	1,219,235	1436	207
21/10/2014; 16 m; 0.8 µm	3300023249_8	99	1,737,575	1754	52
21/10/2014; 19 m; 0.8 µm	3300022838_8	89	1,431,222	1555	180
21/10/2014; 24 m; 0.8 µm	3300023295_7	61	1,100,512	1254	200
4/12/2014; 12 m; 3 µm	3300023231_5	99	1,783,647	1765	23
4/12/2014; 12 m; 0.8 µm	3300023227_6	99	1,783,085	1763	26
4/12/2014; 12 m; 0.1 µm	3300022851_4	99	1,796,868	1770	19
4/12/2014; 13.4 m; 3 µm*	3300031697_14	99	1,807,042	1791	33
4/12/2014; 13.4 m; 0.8 µm*	3300022826_4	99	1,801,610	1778	31
4/12/2014; 13.4 m; 0.1 µm*	3300023292_2	99	1,785,555	1760	28
4/12/2014; 14 m; 3 µm	3300023253_8	99	1,797,888	1783	22
4/12/2014; 14 m; 0.8 µm	3300023233_7	99	1,811,803	1791	24
4/12/2014; 14 m; 0.1 µm	3300022868_7	99	1,777,496	1761	26
3/12/2014; 19 m; 3 µm	3300022860_8	98	1,773,797	1750	22
3/12/2014; 19 m; 0.8 µm	3300022846_6	99	1,797,570	1772	29
**3/12/2014; 19 m; 0.1 µm**	**3300023061_2**	**99**	**1,812,610**	**1797**	**27**

3/12/2014; 24 m; 3 µm	3300022884_9	99	1,735,816	1755	69
3/12/2014; 24 m; 0.8 µm	3300023299_6	99	1,795,237	1771	22
3/12/2014; 24 m; 0.1 µm	3300023256_3	99	1,797,328	1785	22
**Ellis Fjord**					
9/10/2014; 5 m; 0.1 µm	3300031659_20	63	890,084	1006	170
**9/10/2014; 45 m; 3 µm***	**3300031631_9**	**99**	**1,836,564**	**1807**	**32**
9/10/2014; 45 m; 0.8 µm*	3300031741_10	99	1,820,609	1801	31
9/10/2014; 45 m; 0.1 µm*	3300031603_6	99	1,820,941	1799	33
8/10/2014; 60 m; 3 µm	3300031645_24	89	1,450,081	1532	187
8/10/2014; 60 m; 0.8 µm	3300031657_13	99	1,753,701	1756	34
8/10/2014; 60 m; 0.1 µm	3300031601_7	99	1,770,724	1775	46
**Taynaya Bay**					
28/11/2014; 5 m; 0.22 µm	3300038912_10	99	1,808,383	1834	57
28/11/2014; 11 m; 0.22 µm*	3300039187_7	99	1,822,415	1829	24

[A] The numbers prior to the underscore symbol in the IMG Bin ID denotes the IMG Genome ID of the metagenome from which the MAG bin was generated; high-quality MAGs (green background). [B] Bin contamination <1% (black font); bin contamination 1–3% (red font). * Metagenomes from the oxic-anoxic interface. MAGs from AL (AL_ref MAG) and EF (EF_ref MAG) used for FR analyses are highlighted (bold font). For MIMAG (minimum information about MAG) data [11] of the MAGs see Additional file 2: Dataset S1. Filter fractions: 3, 3–20 µm; 0.8, 0.8–3 µm; 0.1, 0.1–0.8 µm; 0.22, 0.22–20 µm.

Table S3 *Ca.* Chlorobium antarcticum AL_ref MAG and EF_ref MAG contigs.

Contig number[A]	Contig ID[B]	Length (bp)	GC content	Read depth[C]
**AL_ref MAG**				
A1	Ga0222700_1000010	109,790	0.54	45
A2	Ga0222700_1000007	177,475	0.54	48
A3	Ga0222700_1000009	111,159	0.53	46
A4	Ga0222700_1002289	3,767	0.49	10
A5	Ga0222700_1000205	12,282	0.52	48
A6	Ga0222700_1001909	4,138	0.49	45
A7	Ga0222700_1000764	6,459	0.47	11
A8	Ga0222700_1000005	182,208	0.51	47
A9	Ga0222700_1000002	276,836	0.53	48
A10	Ga0222700_1000006	177,725	0.53	49
A11	Ga0222700_1000023	41,078	0.5	47
A12	Ga0222700_1000399	8,847	0.5	44
A13	Ga0222700_1000158	13,766	0.52	33
A14	Ga0222700_1000107	16,990	0.52	13
A15	Ga0222700_1000260	10,955	0.52	13
A16	Ga0222700_1003121	3,206	0.51	12
A17	Ga0222700_1000237	11,418	0.52	23
A18	Ga0222700_1000014	58,907	0.51	47
A19	Ga0222700_1000041	29,424	0.51	42
A20	Ga0222700_1000040	29,669	0.5	62
A21	Ga0222700_1000052	26,363	0.5	45
A22	Ga0222700_1000552	7,608	0.49	49
A23	Ga0222700_1000017	44,350	0.5	47
A24	Ga0222700_1000004	197,466	0.52	47
A25	Ga0222700_1000327	9,737	0.52	46
A26	Ga0222700_1000059	24,299	0.52	46
A27	Ga0222700_1000003	216,688	0.54	46
**EF_ref MAG**				
E1	Ga0307987_1001829	11,473	0.52	20
E2	Ga0307987_1000015	116,997	0.54	1229
E3	Ga0307987_1000010	131,454	0.54	1213
E4	Ga0307987_1000128	45,939	0.55	1176
E5	Ga0307987_1015042	3,102	0.52	1055
E6	Ga0307987_1000137	44,410	0.54	1110
E7	Ga0307987_1000149	42,560	0.52	1030
E8	Ga0307987_1000001	271,782	0.52	984
E9	Ga0307987_1000006	162,002	0.53	1071
E10	Ga0307987_1000209	36,702	0.52	1086
E11	Ga0307987_1001038	15,792	0.53	1101
E12	Ga0307987_1000004	178,460	0.53	1150
E13	Ga0307987_1000132	45,348	0.5	871
E14	Ga0307987_1001178	14,683	0.5	463
E15	Ga0307987_1000158	41,780	0.52	76
E16	Ga0307987_1000070	58,906	0.51	922
E17	Ga0307987_1012082	3,606	0.51	485
E18	Ga0307987_1000445	24,876	0.51	889
E19	Ga0307987_1000306	29,343	0.5	883
E20	Ga0307987_1000397	26,253	0.5	844

E21	Ga0307987_1008200	4,637	0.49	850
E22	Ga0307987_1000116	48,411	0.5	860
E23	Ga0307987_1000007	159,945	0.52	977
E24	Ga0307987_1000188	38,654	0.52	941
E25	Ga0307987_1002717	9,042	0.52	1029
E26	Ga0307987_1000481	24,045	0.52	1009
E27	Ga0307987_1000002	216,430	0.54	1073
E28	Ga0307987_1004356	6,757	0.49	80
E29	Ga0307987_1012998	3,422	0.51	16
E30	Ga0307987_1013512	3,330	0.52	14
E31	Ga0307987_1008940	4,380	0.51	13
E32	Ga0307987_1001683	12,043	0.52	22

For details of AL_ref MAG and EF_ref MAG see Additional file 1: Table S2 and Additional file 2: Dataset S1. [A] The contig numbers A1–A27 and E1–E32 correspond to contigs in Figs. 4 and 5, respectively. The scaffold arrangement of MAG contigs is shown using a common background colour, and scaffold sequences that match between AL_ref MAG and EF_ref MAG are shown using the same background colour. EF_ref MAG contigs E28–E32 were not identified in *Ca.* Chlorobium antarcticum MAGs from AL and TB, their coverages were very low in all three systems, and the contigs could not be arranged into scaffolds. [B] The contig IDs are IMG scaffold IDs. *Ca.* Chlorobium antarcticum contigs containing LCRs are shown in red font. Note that a contig containing a LCR may still have "high" read depth where the LCR does not cover the entire contig. [C] Read depth of the AL_ref MAG and EF_ref MAG contigs are from the metagenomes in IMG that the MAGs were generated from.

Table S4 *Ca.* Chlorobium antarcticum LCR gene autoannotations.

LCR number	AL_ref MAG contig, starting position on contig, region length[A]	Seasonal relative coverage of LCRs[B]	AL_ref MAG genes[C]	EF_ref MAG contig, starting position on contig, region length[A]	Relative coverage of LCRs in AL, EF and TB[B]	EF_ref MAG genes[C]
**LCR1**	A1<br>1 bp<br>11 kb length	S: 32%<br>W: 26–28%<br>Sp: 27–31%	Hypothetical protein; [DEAD/DEAH box helicase family protein]<br>Restriction system protein<br>PH (Pleckstrin Homology) domain-containing protein<br>PD-(D/E)XK nuclease superfamily protein<br>ATP-dependent exoDNAse (exonuclease V) beta subunit/superfamily I DNA/RNA helicase<br>Hypothetical protein<br>Hypothetical protein<br>Hypothetical protein<br>Hypothetical protein<br>Uncharacterized protein (DUF4415 family); [BrnA antitoxin family protein]	E1<br>Whole contig<br>11 kb length	AL: 25–32%<br>EF: 3%<br>TB: 69%	Hypothetical protein<br>Uncharacterized protein (DUF4415 family); [BrnA antitoxin family protein]<br>Hypothetical protein<br>Hypothetical protein<br>Hypothetical protein<br>ATP-dependent exoDNAse (exonuclease V) beta subunit/superfamily I DNA/RNA helicase<br>PD-(D/E)XK nuclease superfamily protein<br>Uncharacterized membrane protein YdbT with pleckstrin-like domain<br>Restriction system protein<br>Hypothetical protein; [DEAD/DEAH box helicase family protein]
**LCR2**	A3<br>~79.5 kb<br>~9 kb length	S: 65%<br>W: 65–70%<br>Sp: 67–77%	Phosphatidylinositol alpha-1,6-mannosyltransferase<br>Glycosyltransferase involved in cell wall biosynthesis<br>Glycosyltransferase involved in cell wall biosynthesis<br>Glycosyltransferase involved in cell wall biosynthesis<br>Glycosyltransferase involved in cell wall biosynthesis		AL: 52–61%<br>EF: 66%<br>TB: 67%	Not all genes identified in EF_ref MAG*

			Hypothetical protein<br>Hypothetical protein<br>Hypothetical protein<br>Ubiquinone/menaquinone biosynthesis C-methylase UbiE			
**LCR3**	A4<br>Whole contig<br>4 kb length	S: 37%<br>W: 34–37%<br>Sp: 28–32%	Protease secretion system outer membrane protein<br>Protease secretion system membrane fusion protein<br>ATP-binding cassette subfamily C exporter for protease/lipase	E7<br>~36 kb<br>6 kb length	AL: 25–34%<br>EF: 66%<br>TB: >100%	ATP-binding cassette subfamily C exporter for protease/lipase<br>ATP-binding cassette subfamily C exporter for protease/lipase<br>Protease secretion system membrane fusion protein<br>Protease secretion system outer membrane protein<br>Transposase InsO family protein
**LCR4**	A7<br>Whole contig<br>6 kb length	S: 14%<br>W: 16%<br>Sp: 15–20%	Superfamily I DNA and/or RNA helicase<br>IS5 family transposase<br>Hypothetical protein<br>Acyl-ACP thioesterase<br>DDE family transposase<br>Nitrite reductase/ring-hydroxylating Ferredoxin subunit<br>Hypothetical protein<br>Hypothetical protein<br>Hypothetical protein	E8<br>17 kb<br>11 kb length	AL: 15–20%<br>EF: 94%<br>TB: >100%	IS5 family transposase<br>Hypothetical protein<br>Acyl-ACP thioesterase<br>DDE family transposase<br>Nitrite reductase/ring-hydroxylating Ferredoxin subunit<br>Hypothetical protein<br>Uncharacterized protein YPO0396<br>Uncharacterized protein DUF4194<br>Uncharacterized protein DUF3375
**LCR5**	A13<br>Whole contig<br>14 kb length	S: 70%<br>W: 61–62%<br>Sp: 60–68%	Iron complex outermembrane receptor protein/hemoglobin/transferrin/lactoferrin receptor protein/vitamin B12 transporter<br>Iron complex transport system substrate-binding protein<br>5-Methyltetrahydropteroyltriglutamate--homocysteine methyltransferase<br>Ribonucleoside-triphosphate reductase<br>Pyruvate formate lyase activating enzyme	E14<br>Whole contig<br>15 kb length	AL: 60–70%<br>EF: 44%<br>TB: 79%	Iron complex outermembrane receptor protein/hemoglobin/transferrin/lactoferrin receptor protein/vitamin B12 transporter<br>iron complex transport system substrate-binding protein<br>5-Methyltetrahydropteroyltriglutamate--homocysteine methyltransferase<br>Ribonucleoside-triphosphate reductase<br>Pyruvate formate lyase activating enzyme

			Iron complex transport system permease protein<br>Iron complex transport system ATP-binding protein<br>Iron complex transport system substrate-binding protein<br>Type I restriction enzyme R subunit			Iron complex transport system permease protein<br>Iron complex transport system ATP-binding protein<br>Iron complex transport system substrate-binding protein<br>Type I restriction enzyme R subunit
	A14<br>Whole contig<br>17 kb length	S: 24%<br>W: 9%<br>Sp: 10–21%	Hypothetical protein<br>Predicted amidohydrolase<br>Sugar phosphate isomerase/epimerase<br>Uncharacterized protein<br>Cobaltochelatase CobN<br>Iron complex outermembrane receptor protein<br>Magnesium chelatase subunit D<br>Magnesium chelatase subunit I<br>Cobaltochelatase CobN<br>Iron complex outermembrane receptor protein/hemoglobin/transferrin/lactoferrin receptor protein/vitamin B12 transporter	E15<br>Whole contig<br>1 bp<br>31 kb length	AL: 9–26%<br>EF: 7%<br>TB: 78%	Iron complex outermembrane receptor protein/hemoglobin/transferrin/lactoferrin receptor protein/vitamin B12 transporter<br>Cobaltochelatase CobN<br>Magnesium chelatase subunit I<br>Magnesium chelatase subunit D<br>Iron complex outermembrane receptor protein<br>Cobaltochelatase CobN<br>Uncharacterized protein<br>Sugar phosphate isomerase/epimerase<br>Predicted amidohydrolase<br>Adenosylcobinamide amidohydrolase<br>Iron complex transport system ATP-binding protein<br>Iron complex transport system permease protein<br>Iron complex transport system substrate-binding protein<br>Hypothetical protein<br>Formylglycine-generating enzyme required for sulfatase activity<br>SpoVK/Ycf46/Vps4 family AAA+-type ATPase<br>Molecular chaperone DnaK (HSP70)<br>Molecular chaperone GrpE<br>Hypothetical protein<br>Hypothetical protein
	A15<br>Whole contig<br>11 kb length	S: 26%<br>W: 9–10%<br>Sp: 11–23%	Hypothetical protein<br>Molecular chaperone GrpE<br>Molecular chaperone DnaK (HSP70)<br>SpoVK/Ycf46/Vps4 family AAA+-type ATPase<br>Formylglycine-generating enzyme required for sulfatase activity<br>Hypothetical protein<br>Iron complex transport system substrate-binding protein<br>Iron complex transport system permease protein<br>Iron complex transport system ATP-binding protein<br>Adenosylcobinamide amidohydrolase			

	A16<br>Whole contig<br>3 kb length	S: 25%<br>W: 10%<br>Sp: 11–22%	Threonine dehydrogenase-like Zn-dependent dehydrogenase<br>Anthranilate phosphoribosyltransferase<br>Hypothetical protein			Anthranilate phosphoribosyltransferase<br>Threonine dehydrogenase-like Zn-dependent dehydrogenase
	A17<br>Whole contig<br>11 kb length	S: 59%<br>W: 33–34%<br>Sp: 33–44%	Cobalt-precorrin-5B (C1)-methyltransferase<br>Cobalt-precorrin-5B (C1)-methyltransferase<br>Precorrin-4 methylase/cobalamin biosynthesis protein CbiG<br>Precorrin-6Y C5,15-methyltransferase (decarboxylating)<br>Precorrin-3B methylase/precorrin isomerase<br>Precorrin-2/cobalt-factor-2 C20-methyltransferase<br>Sirohydrochlorin cobaltochelatase<br>Uroporphyrin-III C-methyltransferase<br>Cobalt/nickel transport system ATP-binding protein<br>Cobalt/nickel transport system permease protein<br>Cobalt/nickel transport protein<br>Cobalt/nickel transport system permease protein	E15<br>31 kb<br>11 kb length	AL: 29–59%<br>EF: 8%<br>TB: 72%	Hypothetical protein<br>Cobalt-precorrin-5B (C1)-methyltransferase<br>Cobalt-precorrin-5B (C1)-methyltransferase<br>Precorrin-4 methylase/cobalamin biosynthesis protein CbiG<br>Precorrin-6Y C5,15-methyltransferase (decarboxylating)<br>Precorrin-3B methylase/precorrin isomerase<br>Precorrin-2/cobalt-factor-2 C20-methyltransferase<br>Sirohydrochlorin cobaltochelatase<br>Uroporphyrin-III C-methyltransferase<br>Cobalt/nickel transport system ATP-binding protein<br>Cobalt/nickel transport system permease protein
**LCR6**	A19<br>1 bp<br>2 kb length	S: 27%<br>W: 31–36%<br>Sp: 28–34%	Hypothetical protein<br>Type I restriction enzyme M protein		AL: 42–51%<br>EF: 52%<br>TB: 66%<br>(data for Type I R-M gene only)	Not identified in EF_ref MAG*****
				E17	AL: 56–64%<br>EF: 37%	Hypothetical protein

				Whole contig<br>4 kb length	TB: 64%	Hemoglobin/transferrin/lactoferrin receptor protein<br>Hypothetical protein
**LCR7**	A21<br>1 bp<br>7 kb length	S: 70%<br>W: 58–63%<br>Sp: 62–68%	DNA repair protein RadC<br>F-type H+-transporting ATPase subunit gamma<br>F-type H+-transporting ATPase subunit alpha<br>F-type H+-transporting ATPase subunit b<br>F-type H+-transporting ATPase subunit c<br>F-type H+-transporting ATPase subunit a<br>F1-F0 ATPase (N-ATPase) AtpR subunit<br>ATP synthase protein I<br>F-type H+-transporting ATPase subunit epsilon<br>F-type H+-transporting ATPase subunit beta	E20<br>1 bp<br>7 kb length	AL: 59–71%<br>EF: 69%<br>TB: 91%	DNA repair protein RadC<br>F-type H+-transporting ATPase subunit gamma<br>F-type H+-transporting ATPase subunit alpha<br>F-type H+-transporting ATPase subunit b<br>F-type H+-transporting ATPase subunit c<br>F-type H+-transporting ATPase subunit a<br>F1-F0 ATPase (N-ATPase) AtpR subunit<br>ATP synthase protein I<br>F-type H+-transporting ATPase subunit epsilon<br>F-type H+-transporting ATPase subunit beta
**LCR8**				E28<br>Whole contig<br>7 kb length	AL: 8–12%<br>EF: 8%<br>TB: 1%	Hypothetical protein<br>Hypothetical protein<br>Hypothetical protein<br>Predicted dehydrogenase/threonine dehydrogenase-like Zn-dependent dehydrogenase<br>Uncharacterized heparinase superfamily protein<br>Hypothetical protein<br>UDP-N-acetyl-D-mannosaminuronic acid dehydrogenase
**LCR9**				E29<br>Whole contig<br>3 kb length	AL: <1%<br>EF: <1%<br>TB: 0%	Hypothetical protein<br>FAD/FMN-containing dehydrogenase/Fe-S oxidoreductase<br>Dihydroxy-acid dehydratase

**LCR10**		E30<br>Whole<br>contig<br>3 kb length	AL: ≤2%<br>EF: <1%<br>TB: <1%	Sialate O-acetylesterase<br>Polygalacturonase
**LCR11**		E31<br>Whole<br>contig<br>4 kb length	AL: <1%<br>EF: <1%<br>TB: <1%	Hypothetical protein<br>Hypothetical protein<br>Hypothetical protein
**LCR12**		E32<br>Whole<br>contig<br>12 kb length	AL: ≤1%<br>EF: <1%<br>TB: <1%	Alpha-L-fucosidase<br>Hypothetical protein<br>Hypothetical protein<br>Hypothetical protein<br>Hypothetical protein<br>Heparinase II/III-like protein<br>Tol biopolymer transport system component/Tol biopolymer transport system component

For details of AL_ref MAG and EF_ref MAG see Additional file 1: Tables S2 and S3 and Additional file 2: Dataset S1. *Ca*. Chlorobium antarcticum LCRs within AL (green column heading) and between AL, EF and TB (blue column heading). [A] The approximate starting positions and lengths of the LCRs are shown in Figs. 4 and 5. [B] The percentages are averages of relative coverages of LCRs in merged AL metagenomes from different seasons (summer (S), Dec 2014; winter (W), Jul 2014 and Aug 2014; spring (Sp), Nov 2008, Nov 2013 and Oct 2014) or from different systems (AL, six merged metagenomes; EF, one merged metagenome; TB, one metagenome) as described in Additional file 1: Table S14. Percentages indicate the approximate proportion of *Ca*. Chlorobium antarcticum phylotypes containing the LCRs within the overall *Ca*. Chlorobium antarcticum population. [C] The genes represent IMG autoannotations. The LCRs are arranged from top to bottom in the order of their occurrence along the lengths of AL_ref MAG and EF_ref MAG contigs. * Only some of the low coverage genes on AL_ref MAG contig A3 were identified in EF_ref MAG and they were distributed across the ends of two adjacently placed EF_ref MAG contigs (E5 and E6). The genes on AL_ref MAG contig A19 were not identified in EF_ref MAG. However, FR to a TB_11 m depth MAG containing the genes on contigs A3 and A19 identified them in EF and TB *Ca*. Chlorobium antarcticum populations.

Table S5 Grouping of AL *Ca*. Chlorobium antarcticum LCR genes associated with transport.

Group number (AL_ref MAG contig number)[A]	Seasons and % *Ca*. Chlorobium antarcticum population in which observed[B]	Gene autoannotation[C]	Gene manual annotation and protein sequence identity[D]	Difference between Summer/Winter read depths of genes[E]		
				Wald test statistic	*P*-value	BH-adjusted *P*-value
Group 1 (A13)	**S:** 61%<br>**W:** 57–58%<br>**Sp:** 56–59%; 76% in Oct 2014	Iron complex outer membrane receptor protein/hemoglobin/transferrin/lactoferrin receptor protein/vitamin B12 transporter	86% TonB-dependent receptor *Chlorobium limicola* (RefSeq)	-1.1	0.3	0.4
		Iron complex transport system substrate-binding protein	68% ABC transporter substrate-binding protein (metal-binding TroA-like domain) *Chlorobium limicola* (RefSeq)	-1.2	0.2	0.4
Group 2 (A13)	**S:** 76%<br>**W:** 62–63%<br>**Sp:** 60–70%	Iron complex transport system permease protein	65% Iron ABC transporter permease *Prosthecochloris aestuarii* (RefSeq)	-0.1	0.9	0.9
		Iron complex transport system ATP-binding protein	42% Uncharacterized ABC transporter ATP-binding protein HI_1272 *Haemophilus influenzae* Rd KW20	-0.3	0.8	0.8
		Iron complex transport system substrate-binding protein	23% Fe(3+)-citrate-binding protein YfmC *Bacillus subtilis* subsp. *subtilis* str. 168	-0.4	0.7	0.8
Group 3 (A15)	**S:** 25%<br>**W:** 9%<br>**Sp:** 11–23%	Iron complex transport system substrate-binding protein	25% Uncharacterized lipoprotein MJ0878 (containing Fe/B12 periplasmic-binding domain) *Methanocaldococcus jannaschii*	2	0.05	0.1
		Iron complex transport system permease protein	35% Vitamin B12 import system permease protein BtuC *Klebsiella pneumoniae*	2.3	0.02	0.1
		Iron complex transport system ATP-binding protein	37% Uncharacterized ABC transporter ATP-binding protein MJ0873 (ABC-type cobalamin/Fe-siderophore transporter) *Methanocaldococcus jannaschii*	1.8	0.07	0.2
Group 4 (A14)	**S:** 25%<br>**W:** 9–10%<br>**Sp:** 11–29%	Iron complex outermembrane receptor protein	24% Vitamin B12 transporter BtuB *Salmonella typhimurium*	1.7	0.08	0.2

Group 5 (A14)	**S:** 21%<br>**W:** 7–9%<br>**Sp:** 8–19%	Iron complex outermembrane receptor protein/hemoglobin/transferrin/lactoferrin receptor protein/vitamin B12 transporter	52% TonB-dependent receptor *Prosthecochloris* sp. GSB1 (RefSeq)	1.8	0.07	0.2
Group 6 (A17)	**S:** 61%<br>**W:** 42%<br>**Sp:** 41–50%	Cobalt/nickel transport system ATP-binding protein	45% Cobalt import ATP-binding protein CbiO *Rhodobacter capsulatus*	2.1	0.03	0.1
		Cobalt/nickel transport system permease protein	31% Cobalt transport protein CbiQ *Rhodobacter capsulatus*	2	0.05	0.1
		Cobalt/nickel transport protein	52% Cobalt transport protein CbiN *Nostoc* sp.	1.3	0.2	0.3
		Cobalt/nickel transport system permease protein	Small sequence matches to cobalt transporter CbiM	-1.3	0.2	0.3
Group 7 (A4)	**S:** 37%<br>**W:** 34–37%<br>**Sp:** 28–32%	Protease secretion system outer membrane protein	28% Outer membrane protein TolC *Vibrio cholerae*	-0.6	0.6	0.7
		Protease secretion system membrane fusion protein	32% Alkaline protease secretion protein AprE *Pseudomonas aeruginosa* PAO1	-0.96	0.3	0.5
		ATP-binding cassette subfamily C exporter for protease/lipase	46% Alkaline protease secretion ATP-binding protein AprD *Pseudomonas aeruginosa* PAO1	-1.5	0.1	0.3
Group 8 (A21)	**S:** 76%<br>**W:** 61–66%<br>**Sp:** 66–72%	F-type H+-transporting ATPase subunit gamma	24% ATP synthase gamma chain (AtpG) *Natranaerobius thermophilus*	-0.5	0.6	0.8
		F-type H+-transporting ATPase subunit alpha	78% ATP synthase subunit alpha 1 (AtpA) *Pelodictyon luteolum* DSM 273	-0.4	0.7	0.8
		F-type H+-transporting ATPase subunit b	50% ATP synthase subunit b 1 (AtpF) *Pelodictyon luteolum* DSM 273	-0.2	0.9	0.9
		F-type H+-transporting ATPase subunit c	52% ATP synthase subunit c 1 (AtpE) *Pelobacter carbinolicus* DSM 2380	-0.5	0.6	0.8
		F-type H+-transporting ATPase subunit a	88% ATP synthase subunit a 1 (AtpB) *Pelodictyon luteolum* DSM 273	0.6	0.6	0.7
		F1-F0 ATPase (N-ATPase) AtpR subunit	49% ATP synthase subunit I (AtpR) *Polaribacter* sp. IC073 (RefSeq)	-1.1	0.3	0.4
		ATP synthase protein I	55% AtpZ/AtpI family protein (AtpQ) *Chlorobaculum parvum* (RefSeq)	-0.4	0.7	0.8
		F-type H+-transporting ATPase subunit epsilon	84% F0F1 ATP synthase subunit epsilon (AtpC) *Chlorobium* sp. N1 (UniProtKB)	-0.2	0.9	0.9

	F-type H+-transporting ATPase subunit beta	81% ATP synthase subunit beta 2 (AtpD) *Pelodictyon luteolum* DSM 273	-0.5	0.6	0.8

[A] Genes from LCRs were placed into functional groups. These groups include genes potentially involved in iron transport (Groups 1 and 2), cobalamin transport (Groups 3, 4 and 5), cobalt transport (Group 6), protease export (Group 7) and Na^+ export (Group 8). The AL_ref MAG contig number is specified in parentheses (A1–A27; Additional file 1: Table S3). [B] summer (S), Dec 2014; winter (W), Jul 2014 and Aug 2014; spring (Sp), Nov 2008, Nov 2013 and Oct 2014. The percentages are averages of relative coverages of genes included in each group in merged metagenomes from each season (Additional file 1: Table S14). The values represent the abundance contributions of *Ca*. Chlorobium antarcticum phylotypes containing a functional group of genes. [C] IMG autoannotations of AL_ref MAG genes. [D] Manual annotation was performed by aligning the protein sequences to reference proteins from the UniProtKB/Swiss-Prot database using the ExPASy BLAST+ online service [12], and those with poor alignment or no hits were realigned to reference proteins in the UniProtKB database or RefSeq protein database using the NCBI blastp suite [13] (see the “Methods” section). [E] Statistical analysis was performed using the DESeq2 R package (see the “Methods” section). *P*-values significant at 95% significance level (i.e., *P*-values <0.05) are highlighted with green background. Considering a 5% fraction of false positives to be acceptable, none of the BH-adjusted *P*-values were significant (i.e., no adjusted *P*-values <0.05).

Table S6 AL *Ca*. Chlorobium antarcticum LCR genes associated with cobalamin biosynthesis and cobinamide and pseudocobalamin salvaging.

Function; Season: % *Ca*. Chlorobium antarcticum population in which observed (AL_ref MAG contig number)[A]	Gene	Gene annotation[B]	Protein sequence identity and gene function[C]	Difference between Summer/Winter read depths of genes[D]		
				Wald test statistic	*P*-value	BH-adjusted *P*-value
Cobalamin biosynthesis (anaerobic pathway); **S:** 58% **W:** 28–29% **Sp:** 29–44% (A17)	*cbiD*	Cobalt-precorrin-5B (C1)-methyltransferase	51% Cobalt-precorrin-5B C(1)-methyltransferase *Prosthecochloris aestuarii*	2.2	0.03	0.1
	cbiJ	Cobalt-precorrin-5B (C1)-methyltransferase	30% Cobalt-precorrin-6A reductase *Methanothermobacter thermautotrophicus*	2.2	0.03	0.1
	cbiFG	Precorrin-4 methylase/cobalamin biosynthesis protein CbiG	49% Cobalt-precorrin-4 C(11)-methyltransferase CbiF *Methanocaldococcus jannaschii*<br>31% Cobalt-precorrin-5A hydrolase CbiG *Salmonella typhimurium*	2.5	0.01	0.1
	cbiET	Precorrin-6Y C5,15-methyltransferase (decarboxylating)	32% Cobalamin biosynthesis bifunctional protein CbiET *Bacillus megaterium*	2.8	0.01	0.09
	cbiHC	Precorrin-3B methylase/precorrin isomerase	49% Cobalt-factor III methyltransferase CbiH *Bacillus megaterium*<br>36% Cobalt-precorrin-8 methylmutase CbiC *Leptospira interrogans* serovar Lai str. 56601	3.1	0.002	0.08
	cbiL	Precorrin-2/cobalt-factor-2 C20-methyltransferase	28% Precorrin-2 C(20)-methyltransferase *Pseudomonas aeruginosa*	2.4	0.02	0.1
	cbiK	Sirohydrochlorin cobaltochelatase	26% Sirohydrochlorin cobaltochelatase CbiKP *Desulfovibrio vulgaris*	2.7	0.01	0.09
	cysG	Uroporphyrin-III C-methyltransferase	44% Uroporphyrinogen-III C-methyltransferase *Bacillus megaterium*	2.6	0.01	0.1
Cobinamide and pseudocobalamin salvage; **S:** 25% **W:** 8–10% **Sp:** 10–20%	*cbiZ*	Adenosylcobinamide amidohydrolase	33% Uncharacterized protein MJ1613 (containing CbiZ domain) *Methanocaldococcus jannaschii* DSM 2661	2.1	0.03	0.1

(A15)						
Cobalt chelatase;<br>**S:** 25%<br>**W:** 9%<br>**Sp:** 11–22%<br>(A14)	*cobN*	Cobaltochelatase CobN	34% Aerobic cobaltochelatase subunit CobN *Sinorhizobium* sp.	2.1	0.04	0.1
Magnesium chelatase;<br>**S:** 26%<br>**W:** 9%<br>**Sp:** 10–22%<br>(A14)	*bchD*	Magnesium chelatase subunit D	28% Magnesium-chelatase subunit D *Rhodobacter capsulatus* SB 1003	2	0.05	0.1
	bchI	Magnesium chelatase subunit I	55% Magnesium-chelatase subunit I homolog *Synechocystis* sp. PCC 6803	2.2	0.03	0.1
	bchH	Cobaltochelatase CobN	28% Magnesium-chelatase subunit H *Rhodobacter capsulatus*	2.3	0.02	0.1

Cobalamin riboswitch sequences[E]

AL_ref MAG contig number: region on contig (downstream gene)	Season: % *Ca*. Chlorobium antarcticum population in which observed	Nucleotide sequence identity and potential function (region on Cpv-DSM genome)	Difference between Summer/Winter read depths of sequences[D]		
			Wald test statistic	*P*-value	BH-adjusted *P*-value
**A13:** 1462–1652 (*metE*)	**S:** 74%<br>**W:** 58–65%<br>**Sp:** 65–70%	Identified through Rfam database	-0.6	0.6	0.6
**A13:** 4165–4396 (*nrdD*)	**S:** 75%<br>**W:** 56–62%<br>**Sp:** 64–68%	70% Cobalamin riboswitch (2) *Chlorobium phaeovibrioides* DSM265	-0.1	0.9	0.9
**A14:** 16199–163334 (*btuB*)	**S:** 28%<br>**W:** 9–12%<br>**Sp:** 10–22%	75% Cobalamin riboswitch (1) *Chlorobium phaeovibrioides* DSM265	2	0.04	0.1
**A15:** 6088–6324 (*btuF*)	**S:** 27%<br>**W:** 9–13%<br>**Sp:** 10–24%	74% Cobalamin riboswitch (4) *Chlorobium phaeovibrioides* DSM265<br>72% Cobalamin riboswitch (2) *Chlorobium phaeovibrioides* DSM265	1.5	0.1	0.3
**A16:** 2343–2582	**S:** 27%	76% Cobalamin riboswitch (4)	2	0.03	0.1

(NA; sequence located near one end of contig)	**W:** 9%<br>**Sp:** 12–24%	*Chlorobium phaeovibrioides* DSM265<br>76% Cobalamin riboswitch (2)<br>*Chlorobium phaeovibrioides* DSM265			
**A17:** 432–231<br>(NA; sequence located near one end of contig)	**S:** 68%<br>**W:** 29%<br>**Sp:** 31–43%	85% Cobalamin riboswitch (3)<br>*Chlorobium phaeovibrioides* DSM265	3.5	0.0005	0.02

[A] The AL_ref MAG contig number is specified in parentheses (A1–A27; Additional file 1: Table S3). summer (S), Dec 2014; winter (W), Jul 2014 and Aug 2014; spring (Sp), Nov 2008, Nov 2013 and Oct 2014. The percentages are averages of relative coverages of genes in merged metagenomes from each season (Additional file 1: Table S14). These percentages indicate the approximate contributions of *Ca*. Chlorobium antarcticum phylotypes containing these genes or gene clusters to the overall *Ca*. Chlorobium antarcticum population in AL from each season. [B] IMG autoannotations of AL_ref MAG genes. [C] Manual annotation was performed by aligning the protein sequences to reference proteins from the UniProtKB/Swiss-Prot database using the ExPASy BLAST+ online service [12], and those with poor alignment or no hits were realigned to reference proteins in the UniProtKB database or RefSeq protein database using the NCBI blastp suite [13]. Some genes (*cbiFG*, *cbiET* and *cbiHC*) coded for bifunctional proteins (see the “Methods” section). [D] Statistical analysis was performed using the DESeq2 R package (see the “Methods” section). *P*-values significant at 95% significance level (i.e., *P*-values <0.05) are highlighted with green background. Considering a 5% fraction of false positives to be acceptable, only one of the BH-adjusted *P*-values was significant (i.e., adjusted *P*-value <0.05). [E] The potential cobalamin riboswitch sequences in *Ca*. Chlorobium antarcticum were identified by aligning AL_ref MAG contigs to four cobalamin riboswitch sequences from the Cpv-DSM265 genome using the NCBI blastn suite [13]. The four Cpv-DSM265 cobalamin riboswitches are: (1) region 905898–906121; (2) region 906493–906733; (3) region 911107–911313; and (4) region 913712–913952. The *Ca*. Chlorobium antarcticum cobalamin riboswitch sequences were verified, and additional sequences were identified, using the Rfam database [14, 15]. *btuB*, vitamin B12 transporter BtuB; *btuF*, vitamin B12-binding protein BtuF; *nrdD*, ribonucleoside-triphosphate reductase; *metE*, 5-methyltetrahydropteroyltriglutamate--homocysteine methyltransferase; *nrdD*, ribonucleoside-triphosphate reductase; NA, not applicable.

Table S7 EF_ref MAG genes with SNPs in AL and TB metagenomes.

Contig number[A]	Gene locus ID[B]	Gene annotation[C]	All Ace Lake[D]	Dec 2014 Ace Lake[D]	Taynaya Bay[D]
**E1**	Ga0307987_100182910	Hypothetical protein	+	+	+
**E2**	Ga0307987_100001560	Cytochrome c553	+	+	+
	Ga0307987_1000015111	Carboxylesterase	+	+	-
**E3**	Ga0307987_100001056	Regulator of protease activity HflC (stomatin/prohibitin superfamily)	+	+	-
	Ga0307987_100001060	KUP system potassium uptake protein	+	+	-
	Ga0307987_1000010121	DNA-directed RNA polymerase subunit beta'	+	+	-
**E4**	Ga0307987_100012814	Peptidylprolyl isomerase/peptidyl-prolyl cis-trans isomerase D	+	+	+
	Ga0307987_100012827	Ribosome-associated toxin RatA of RatAB toxin-antitoxin module	+	-	-
**E7**	Ga0307987_10001498	Uncharacterized protein DUF4405	+	+	-
	Ga0307987_100014913	Lipid-A-disaccharide synthase	+	+	-
**E8**	Ga0307987_10000019	D-alanyl-D-alanine carboxypeptidase (penicillin-binding protein 5/6)	+	+	-
	Ga0307987_100000156	Hypothetical protein	+	+	-
	Ga0307987_100000159	GTP cyclohydrolase I	+	+	-
	Ga0307987_100000173	Ferrochelatase	+	+	+
	Ga0307987_100000192	Ribonuclease Y	+	+	-
	Ga0307987_1000001113	Exonuclease SbcC	+	+	-
	Ga0307987_1000001124	Hypothetical protein	+	+	-
	Ga0307987_1000001141	Molecular chaperone DnaK	+	+	-
	Ga0307987_1000001165	Uncharacterized membrane protein/dolichol kinase	+	+	-
	Ga0307987_1000001182	O-acetylhomoserine (thiol)-lyase	+	+	-
	Ga0307987_1000001184	Integrase/recombinase XerD	+	+	-
	Ga0307987_1000001187	ATP-dependent RNA helicase DeaD	+	+	-
	Ga0307987_1000001198	Ribonucleoside-diphosphate reductase alpha chain	+	+	-
	Ga0307987_1000001208	L-aspartate oxidase	+	+	-
	Ga0307987_1000001226	Rod shape-determining protein MreB	+	+	-
	Ga0307987_1000001266	Hypothetical protein	+	+	-

**E9**	Ga0307987_100000611	Magnesium transporter	+	+	-
	Ga0307987_100000622	2-Succinyl-5-enolpyruvyl-6-hydroxy-3-cyclohexene-1-carboxylate synthase	+	+	+
	Ga0307987_100000647	NTE family protein	+	+	-
	Ga0307987_100000656	Excinuclease ABC subunit B	+	+	-
	Ga0307987_100000657	GTP pyrophosphokinase	+	+	+
	Ga0307987_100000675	NAD(P)-dependent dehydrogenase (short-subunit alcohol dehydrogenase family)	+	+	+
	Ga0307987_1000006109	1-Deoxy-D-xylulose-5-phosphate synthase	+	+	-
	Ga0307987_1000006111	Glycosidase	+	+	+
	Ga0307987_1000006112	DNA helicase-2/ATP-dependent DNA helicase PcrA	+	+	-
	Ga0307987_1000006130	ATP-binding cassette subfamily C protein CydC	+	+	+
**E10**	Ga0307987_10002095	Dihydroflavonol-4-reductase	+	+	-
	Ga0307987_10002096	Recombination protein RecA	+	+	+
	Ga0307987_100020912	Cystathionine beta-synthase/cysteine synthase A	+	+	-
	Ga0307987_100020936	3-Vinyl bacteriochlorophyllide hydratase	+	+	+
**E12**	Ga0307987_100000430	RNA recognition motif-containing protein	+	+	-
	Ga0307987_100000443	CTP synthase	+	-	-
	Ga0307987_100000466	Threonyl-tRNA synthetase	+	+	-
	Ga0307987_100000478	Uncharacterized protein	+	+	-
	Ga0307987_100000480	Hypothetical protein	+	+	-
	Ga0307987_100000498	Aspartate aminotransferase-like enzyme	+	+	-
	Ga0307987_1000004103	Hypothetical protein	+	+	+
	Ga0307987_1000004118	Small subunit ribosomal protein S12	+	+	+
	Ga0307987_1000004120	Elongation factor G	+	+	-
	Ga0307987_1000004121	Elongation factor Tu	+	+	-
	Ga0307987_1000004124	Large subunit ribosomal protein L4	+	+	-
	Ga0307987_1000004128	Large subunit ribosomal protein L22	+	+	+
	Ga0307987_1000004139	Large subunit ribosomal protein L6	+	+	-
	Ga0307987_1000004144	Preprotein translocase subunit SecY	+	+	+
	Ga0307987_1000004151	Large subunit ribosomal protein L17	+	+	-
	Ga0307987_1000004163	Starch synthase	+	+	-

	Ga0307987_1000004174	Putative hemolysin	+	+	-
	Ga0307987_1000004181	Bacteriochlorophyll c synthase	+	+	-
**E13**	Ga0307987_10001321	Uncharacterized protein involved in exopolysaccharide biosynthesis/Mrp family chromosome partitioning ATPase	+	+	-
	Ga0307987_10001322	Protein involved in polysaccharide export with SLBB domain	+	+	-
**E15**	Ga0307987_100015828	Precorrin-3B methylase/precorrin isomerase	+	+	-
**E16**	Ga0307987_100007025	Arsenite-transporting ATPase	+	+	-
	Ga0307987_100007033	Putative salt-induced outer membrane protein YdiY	+	+	-
	Ga0307987_100007039	Cell fate regulator YaaT (PSP1 superfamily)	+	+	+
	Ga0307987_100007045	Membrane protein DedA with SNARE-associated domain	+	+	-
	Ga0307987_100007049	Cytochrome c/cytochrome c554/c'-like protein	+	+	-
**E17**	Ga0307987_10120822	Hemoglobin/transferrin/lactoferrin receptor protein	+	+	+
**E18**	Ga0307987_10004454	Dicarboxylate transport/dicarboxylate transport/dicarboxylate transport	-	-	+
**E22**	Ga0307987_100011610	Long-chain acyl-CoA synthetase	+	+	-
	Ga0307987_100011619	Polyketide cyclase/dehydrase/lipid transport protein	+	+	-
	Ga0307987_100011630	Hypothetical protein	+	+	-
**E23**	Ga0307987_100000728	Acetyl-CoA carboxylase carboxyltransferase component	+	+	-
	Ga0307987_100000773	Peptide/nickel transport system permease protein	+	+	-
	Ga0307987_1000007147	UDP-2,3-diacylglucosamine pyrophosphatase LpxH	+	+	-
	Ga0307987_1000007153	Putative porin/putative porin	+	+	-
**E24**	Ga0307987_10001884	Glycosyltransferase involved in cell wall biosynthesis	+	-	-
**E26**	Ga0307987_10004818	Phosphomethylpyrimidine synthase	+	-	-
**E27**	Ga0307987_10000026	Molybdate transport system ATP-binding protein	+	+	-
	Ga0307987_100000214	Lipoprotein-releasing system ATP-binding protein	+	+	+

	Ga0307987_100000249	Excinuclease ABC subunit A	+	+	+
	Ga0307987_100000261	Outer membrane protein assembly factor BamA/outer membrane protein assembly factor BamA/outer membrane translocation and assembly module TamA	+	+	-
	Ga0307987_100000285	Integrase/recombinase XerC	+	+	-
	Ga0307987_100000292	Pyruvate-ferredoxin/flavodoxin oxidoreductase	+	+	-
	Ga0307987_1000002109	6-Phosphofructokinase 1	+	+	-
	Ga0307987_1000002125	Acetylornithine aminotransferase	+	+	-
	Ga0307987_1000002127	NADH dehydrogenase	+	+	-
	Ga0307987_1000002134	SSS family solute:Na+ symporter	+	+	-
	Ga0307987_1000002185	AGZA family xanthine/uracil permease-like MFS transporter	+	+	-
	Ga0307987_1000002190	Heat-inducible transcriptional repressor	-	-	+

Total EF_ref MAG genes containing SNPs	89
MAG genes with SNPs only in AL metagenomes	68 (64 of these also in Dec 2014 Ace Lake metagenome)
MAG genes with SNPs only in TB metagenome	2
MAG genes with SNPs in both AL and TB metagenomes	19

[A] For details of EF_ref MAG see Additional file 1: Tables S2 and S3 and Additional file 2: Dataset S1. [B] IMG gene locus IDs. [C] IMG autoannotations of EF_ref MAG genes. The genes are arranged from top to bottom in the order of occurrence along the lengths of EF_ref MAG contigs. [D] The AL, EF and TB oxic-anoxic interface metagenomes used for FR and SNP analyses are listed in Additional file 1: Table S14. All Ace Lake, SNP data from all AL oxic-anoxic interface merged metagenomes; Dec 2014 Ace Lake, SNP data from only Dec 2014 AL merged metagenome; Taynaya Bay, SNP data from only TB 11 m depth metagenome. The Dec 2014 AL SNP data are shown separately to enable comparison with TB SNP data (sampled in Nov 2014), in order to allow a comparison that avoided possible bias in the number of SNPs observed due to more metagenomes being available from AL than TB; this comparison showed 64 AL SNPs compared to 2 TB SNPs. Only the EF_ref MAG contigs and genes with SNPs in at least one metagenome from AL, EF or TB are shown (also see Fig. 5). No SNPs in EF_ref MAG genes were identified in EF metagenomes. Genes in EF_ref MAG LCRs are in red font; +, SNPs identified; -, SNPs not identified.

Table S8 CRISPR-Cas defence system genes in some members of Chlorobiaceae family.

Species name	CRISPR-Cas system subtype
Chlorobium phaeovibrioides DSM 265	I-C
C. phaeovibrioides GrTcv12[A]	I-F
Chlorobium chlorochromatii CaD3	I-C
Chlorobium luteolum DSM 273	I-C
Chlorobium luteolum CIII[B]	I-C
Chlorobaculum tepidum TLS[C]	I-C and I-E
Chlorobium phaeobacteroides DSM 266	I-C and III-A
C. phaeobacteroides BS1	III-A and I-E
Chlorobaculum parvum NCIB 8327	III-A
Chlorobium limicola DSM 245[D]	I-B and III-B
Chlorobium limicola strain Frasassi[D]	III-A
Ca. Chlorobium antarcticum	I-E

CRISPR-Cas system subtype determined from CRISPR-Cas genes annotated in NCBI Chlorobiaceae genomes relative to *Ca*. Chlorobium antarcticum (orange font). CRISPR-Cas systems have previously been reported for some of these *Chlorobium*: [A] [16], [B] [17], [C] [18], [D] [19].

Table S9 *Ca*. Chlorobium antarcticum defence genes.

Defence system	Subsystem type	Gene autoannotation[A]	Protein sequence identity (%) and gene function[B]
R-M system	**Type IV** restriction endonuclease	Restriction system protein	39%; Mrr restriction system protein *Escherichia coli*
		Restriction system protein	38% Mrr restriction system protein *Escherichia coli*
	**Type I** R-M system	Type I restriction enzyme M protein	38% Putative type I restriction enzyme MpnORFDP M protein *Mycoplasma pneumoniae*
		Type I restriction enzyme R subunit	34% Type-1 restriction enzyme R protein *Staphylococcus saprophyticus* subsp. *Saprophyticus*
CRISPR-Cas system	**Subtype I-E** CRISPR-Cas system	CRISPR-associated protein Cas2	28% CRISPR-associated endoribonuclease Cas2 *Escherichia coli*
		CRISPR-associated protein Cas1	79% CRISPR-associated endonuclease Cas1 *Chlorobaculum tepidum*
		CRISPR system Cascade subunit CasD	29% CRISPR system Cascade subunit CasD *Escherichia coli*
		CRISPR system Cascade subunit CasC	34% CRISPR system Cascade subunit CasC *Escherichia coli*
		CRISPR system Cascade subunit CasE	24% CRISPR system Cascade subunit CasE *Escherichia coli*
		CRISPR system Cascade subunit CasB	31% CRISPR-associated protein Cse2 *Thermus thermophilus*
		CRISPR system Cascade subunit CasA	61% CRISPR-associated protein, Cse1 family *Prosthecochloris aestuarii* (UniProtKB)
		CRISPR-associated endonuclease/helicase Cas3	31% CRISPR-associated nuclease/helicase Cas3 *Streptococcus thermophilus*
BREX system	Not found	-	-
DISARM system	Not found	-	-
T-A system	**ParDE type II** T-A system	Antitoxin ParD1/3/4	35% Antitoxin ParD *Mycobacterium bovis*
		Toxin ParE1/3/4	28% Toxin ParE3 *Caulobacter vibrioides*
	Antitoxin module of a **RelFG type II** T-A system	PHD/YefM family antitoxin component YafN of YafNO toxin-antitoxin module	30% Antitoxin RelF *Mycobacterium tuberculosis*
	Antitoxin module of a **BrnTA type II** T-A system	Uncharacterized protein (DUF4415 family)	94% BrnA antitoxin family protein *Chlorobium limicola* (RefSeq)

	Antitoxin module of an **AbiE type IV** T-A system	Transcriptional regulator with AbiEi antitoxin domain of type IV toxin-antitoxin system	53% Type IV toxin-antitoxin system AbiEi family antitoxin domain-containing protein *Chlorobium phaeobacteroides* (RefSeq)

[A] IMG autoannotations of AL_ref MAG genes. [B] Manual annotation was performed by aligning the protein sequences to reference proteins from the UniProtKB/Swiss-Prot database using the ExPASy BLAST+ online service [12], and those with poor alignment or no hits were realigned to reference proteins in the UniProtKB database or RefSeq protein database using the NCBI blastp suite [13].

Table S10 Spacer and repeat sequences in AL, EF and TB *Ca*. Chlorobium antarcticum MAGs.

Sequence name[A]	Sequence	Sequence length (bp)	System
CRISPR spacers			
Spc1	TTGCTTCTATCATGATTTGATTCCTCCTATAAG	33	AL
Spc2	CAGGAAAGATGCGTATGCGTGGCGGAAAGGCT	32	AL
Spc3	TCAGTGCTGGGGTAAAGGCGACGACGGCCGGATA	34	AL
Spc4	TTCTATTAGATCAACTGGAAATGGAGCAGGGTG	33	AL
Spc5	CAATGAATTTCACCAACTCAAATCTGGCATTAA	33	AL
Spc6	TCATGCGCCGCCTGCTCCGCGAGCTGGCAACCA	33	AL
Spc7	GCGATAAAGACCGCGTAGCACAGGAAACTGAGG	33	AL
Spc8	TACAACCTCATAGCTTTTGTAGATTTCTTGCAA	33	AL
Spc9	ACCGCCCCCCGCCCGCATAAGGTCATCAGCCTG	33	AL
Spc10	TGACACAGGGGTTTTGATCGACAAAGTTGTGTG	33	AL
Spc11	TTCTGAGAAGTACTGGATCAGGGTTGACTCTTG	33	AL
Spc12	GGCTAGCCTTAGTGGCCACAAAGACTGGAACCA	33	AL
Spc13	TCACAGTTGACGATCCCTGGTCTGATGCTATGA	33	AL
Spc14	CTTGGGGTGTATCAGGCGTCAGGGTTGACAGATG	34	AL
Spc15	TAGCTTTGCTGTAATATGGTCACCTTATCATCTA	34	AL
Spc16	GGCAGGGATAACAGAGCTGCGCAGTCAAGTAAAA	34	AL
Spc17	CCCAACGCTAACGCTAGTTGATTAGCGTCAGGGA	34	AL
Spc18	ACGCAGTTGAGTATCAAGAAATTACATCCGCGA	33	AL
Spc19	CCCCGCTGGAAGTATCGATTAATGGGAAGCTTG	33	AL
Spc20	CATATCAGCAACAATGGATTGCACCTGTCACTG	33	AL
Spc21	GCGGCGCTGGTTGCCATTGAAAAGGTATCAGCAG	34	AL
Spc22	ACACAATAAAACCGTGGAGGATTTATGCCGTCG	33	AL
Spc23	CTCTGGATGACGGTCAACCCAGCTGCCGGAAGAA	34	AL
Spc24	TAGACTGGGCGGAGTTTGACAAGCTATGCGGGA	33	AL
Spc25	TCACGTTATTTGGCATAAGCCTGGCGGCGGTAT	33	AL
Spc26	GCTGCCGTGCCGTCTGTTCCGCCAACGGCAAAT	33	AL
Spc27	CCACACCAGCTCAGATAGAAGCATGACACCCAA	33	AL
Spc28	TATATGCAGGCTGAGAAAGCGCGGGCGGGTCTA	33	AL
Spc29	CAAGGTCATGGCGGATTCGCTGGCAACTCAGAGC	34	AL
Spc30	CGCTTATAAATTAGAAACGATGCAGTGGGTCAA	33	AL
Spc31	CGAACCAGCCAAACGCCGGTAGCTTTCTGTTCC	33	AL
Spc32	TCGTCTTGCCGCGCCAAGGGAATCAACGCCTAT	33	AL
Spc33	TCCGCATCCCACAAGATCGGTGAGAACCTCGTCG	34	AL
Spc34	TCCATTCGAATGTCCTGCGCCACATGCCTGCCT	33	AL
Spc35	CACCGTCGTCGCCATAGCTTTTAGCTCTGTGAG	33	AL
Spc36	CACCGGCAAAGTCATAGCTTTTAGCTCCGTGAG	33	AL
Spc37	CAAAAGCCGCGTCGAAAGGCACATATACTTCCG	33	AL
Spc38	CGGAAGTATATGTGCCTTTCGACGCGGCTTTTG	33	AL
Spc39	CTCACGGAGCTAAAAGCTATGACTTTGCCGGTG	33	AL
Spc40	CTCACAGAGCTAAAAGCTATGGCGACGACGGTG	33	AL

Spc41	AGGCAGGCATGTGGCGCAGGACATTCGAATGGA	33	AL
Spc42	CGACGCGGCTGGTTTTGGCCTTTCTACGGTCAA	33	AL
Spc43	AATCGCGAGCGAGCGGGCTGGCTCTGGCTGCT	32	AL
Spc44	CCTCTGGCTGATCATCCCAAATGTTCGGAAGC	32	AL
Spc45	CCGGCAAAGTCATAGCTTTTAGCTCCGTGA	30	AL
Spc46	AAAGCCGCGTCGAAAGGCACATATACTTCC	30	AL
Spc47	CTGTATGCCCGGGACACTCGGAGACCTCGGTC	32	AL
Spc48	TACGGGCCCAGAGTCAGGCCGATGTGGAGGGT	32	AL, EF
Spc49	TACCAATCTCCAAGGAACGACCGAAGCCGTG	31	AL
Spc50	GATGCACGCGACATTCCGCGCGCTGGCGAGA	31	AL
Spc51	CGTCATCGCACCACCAGCCAATCCGGTATAA	31	AL
Spc52	CGACGAGGTTCTCACCGATCTTGTGGGATGCGGA	34	AL
Spc53	GTTGCTGACCGCATTTTTACAAAGCTTGACAC	32	AL
Spc54	GCTGATTCACTGGCAACTCAGAGCACTGACAA	32	AL
Spc55	TCTACTTTCGTCTGCGTTGGTATCAGCTCCCA	32	AL
Spc56	TCGCCTGAGTTAAAAGCAAGGCCGTATAAAGT	32	AL
Spc57	TATCAGGTTTCATTTTTTTTCTCTCTCCCTTGA	33	AL
Spc58	TGTCATTTCATGTGTCATTTTTGTATCCTCTTG	33	AL
Spc59	TATTTCTTCGGTGGCATATCAGAATTTGAGCTTA	34	AL
Spc60	TCGCGTCCTCCTCATCCACTATCCCCGCACCGTA	34	AL
Spc61	GGCAGGCTAACGATATGCAATCAGATAGTTGG	32	AL
Spc62	TAAGGCCGTCAGTCTGAGAGATTCGTTCATGTGA	34	AL
Spc63	TAGACTGGGGTGAGTTCGACAAGCTATGTATGA	33	AL
Spc64	TAGATGGCTGGGGTGTCATGCTGCCATCCTCGC	33	AL
Spc65	TGGATACAATGGACGATGGACCGCTGGAAAGGT	33	AL
Spc66	TAGAGAGAGGAACGATCTCCTCGACCTATCCCG	33	AL
Spc67	CAGCAGCAGCGTAGAAAAGCAGCTGCGCATTTTC	34	AL
Spc68	CTGTGCCCGGGGCTTTTTCCGGGGGTGGGCTTA	34	AL
Spc69	CATGCATCAAGACGTTATCACATCGCTATTTAG	33	AL
Spc70	TGTAGAGAGAGGTCTTCCTGCACTTATTCCGTTTA	35	AL
Spc71	CCACACGAGCAACGATGGCAGCATGACACCCAA	33	AL
Spc72	TGCGGTACGACGCGGATGGCCTAGGGGCCGGGG	33	AL
Spc73	CAAGAGGAGTACGAGATCAAGGCTGAGGAGGAGG	34	AL
Spc74	CTAAGTCTACATCCTCCGCGTCATCAAACAGGG	33	AL
Spc75	TATCTGTCATATCGTCGCACAACAATATAGGCA	33	AL
Spc76	TCAGACTTGTATGTGCTCCCAGCAGGAATAATA	33	AL
Spc77	CGAAAGGGCCTTGAACGGGCATACTGGGGTAGC	33	AL
Spc78	GGCAATTAGGTTTTTAAGTCCGCTCATTGCAG	32	AL
Spc79	TGGAACAAGCACAGAGGGAGCGATAATGGCCGCA	34	AL
Spc80	TAAGCCCCCCGGAAATAAGCCCCCGGACCTCTCG	34	AL
Spc81	CTAAGATATAGCTTGCAGGTTAATTATATTTTG	33	AL
Spc82	CCAACAACAACCATGAGACACTACTACGCTTTA	33	AL
Spc83	TTGCACATGTTTTTATATCAGGCTTCTTCGGGTG	34	AL, TB
Spc84	CTTCAGGGGAGCATGGAACTCGCTTTCCGGGGC	33	AL

Spc85	CAAAAGCCAGATCATCCTCACCCGACACCATAC	33	AL, TB
Spc86	TGTATACGCGTCAATTTTTACAATGACACTCC	33	AL
Spc87	CTGCTACGGAGGCGTTGCGGAGCAGGCGCAAAGC	34	AL
Spc88	TCTCCGACTCTGTCAAAGAAAGCCTTGCCGGAG	33	AL
Spc89	CCCAAGGTGGAACCCTGTCGA	21	AL, TB
Spc90	CACCACGTCGCTCTCGTCGACGCTTAAAGCCAT	33	AL, TB
Spc91	CCAGGCGTGGATATGGCCGGCGATAGCCTTCCG	33	AL
Spc92	TTATCGGACTCGCGACGGAGCTGCTCAGTCTCG	33	AL, TB
Spc93	TGGGATCAATGTCGTCGTCGGCAAGACCGATGC	33	AL
Spc94	TGGAGATCAAGCTACTGAGCCTCCATCTCAAAA	33	AL, TB
Spc95	CGGGAGATTTATGGGACAAGAAATGACGGCAGG	33	AL, TB
Spc96	CAGCCGTGCGCTCGGTATCGGAGTATGTTGCAA	33	AL
Spc97	TCAGAAAGAACGCAAGCACTGGCGATGCTGAAG	33	AL
Spc98	CGTTCTGGCGGCCGTCTTCGACTTCGCCACTGGGA	35	AL, TB
Spc99	TAGAGAGCTCAGGGCGGAGTGGGCCATCGTCAA	33	AL, EF, TB
Spc100	CATCGGCATATTTGACGCTATCAACCTCGTCGT	33	AL, EF, TB
Spc101	TACGGAGCCCGACACCTCCGCGCTTGAAGCCGA	33	AL, EF, TB
Spc102	CGGCAAGACGACGATGGACGAGCTTGGGTCCAA	33	AL, EF, TB
Spc103	CTCCACATCGCTCTCGTCGACGCTTAAAGCCAT	33	AL, EF, TB
Spc104	CGACAAAGCGCTATCAGTGTGCCACCCGAACGA	33	AL, EF
Spc105	CTCGACGGAGTTGATGAAGTCGGACACGACCGA	33	AL, EF
Spc106	TAGGCCTCGTACACGATGGTGTGCCCGCCACGG	33	AL
Spc107	CGACAGCCCTCATTTTCTCGGCACTTGTCGAAT	33	AL
Spc108	TACTTTCCGTATCGATGTGGGGGGTGATTCCGA	33	AL, TB
Spc109	CAAGTCCCTCCTCTTCCATATACTTGAACTTCT	33	AL, TB
Spc110	TTATCTTGCCTGTCTCGGCCTGCTCTTTTGCA	32	AL, TB
Spc111	TGCTACCGGGCCGGAATCGACAGAAAAGGCATG	33	AL, TB
Spc112	CATGAGCTCGTCCCGATGCAAAAGCCTCTCCTG	33	AL, TB
Spc113	TCTCCATCAGCCACCCTACCCGAATCGCCGCACG	34	AL, TB
Spc114	CAAAAAGCCGGAAAATCGGGATCAAATTTCTCA	33	AL, TB
Spc115	CGAGACGGGCGGGGGGCTGACTGGCGCGCTGGA	33	AL, TB
Spc116	TAGTAAAGACTCGCCCGATCCATGCTGCGTCAGG	34	AL, EF, TB
Spc117	CACCCGAAGAAGCCTGATATAAAAACATGTGCAA	34	AL
Spc118	TAAAGCGTAGTAGTGTCTCATGGTTGTTGTTGG	33	AL
Spc119	CAAAATATAATTAACCTGCAAGCTATATCTTAG	33	AL
Spc120	CGAGAGGTCCGGGGGCTTATTTCCGGGGGGCTTA	34	AL
Spc121	TGCGGCCATTATCGCTCCCTCTGTGCTTGTTCCA	34	AL
Spc122	CTGCAATGAGCGGACTTAAAAACCTAATTGCC	32	AL
Spc123	GCTACCCCAGTATGCCCGTTCAAGGCCCTTTCG	33	AL
Spc124	TATTATTCCTGCTGGGAGCACATACAAGTCTGA	33	AL
Spc125	TGCCTATATTGTTGTGCGACGATATGACAGATA	33	AL

Spc126	CCCTGTTTGATGACGCGGAGGATGTAGACTTAG	33	AL
Spc127	CCTCCTCCTCAGCCTTGATCTCGTACTCCTCTTG	34	AL
Spc128	CCCCGGCCCCTAGGCCATCCGCGTCGTACCGCA	33	AL
Spc129	TTGGGTGTCATGCTGCCATCGTTGCTCGTGTGG	33	AL
Spc130	TAAACGGAATAAGTGCAGGAAGACCTCTCTCTACA	35	AL
Spc131	CTAAATAGCGATGTGATAACGTCTTGATGCATG	33	AL
Spc132	TAAGCCCACCCCCGGAAAAAAGCCCCGGGCACAG	34	AL
Spc133	GAAAATGCGCAGCTGCTTTTCTACGCTGCTGCTG	34	AL
Spc134	CGGGATAGGTCGAGGAGATCGTTCCTCTCTCTA	33	AL
Spc135	ACCTTTCCAGCGGTCCATCGTCCATTGTATCCA	33	AL
Spc136	GCGAGGATGGCAGCATGACACCCCAGCCATCTA	33	AL
Spc137	TCATACATAGCTTGTCGAACTCACCCCAGTCTA	33	AL
Spc138	TCACATGAACGAATCTCTCAGACTGACGGCCTTA	34	AL
Spc139	CCAACTATCTGATTGCATATCGTTAGCCTGCC	32	AL
Spc140	TACGGTGCGGGGATAGTGGATGAGGAGGACGCGA	34	AL
Spc141	TAAGCTCAAATTCTGATATGCCACCGAAGAAATA	34	AL
Spc142	CAAGAGGATACAAAAATGACACATGAAATGACA	33	AL
Spc143	TCAAGGGAGAGAGAAAAAAAATGAAACCTGATA	33	AL
Spc144	ATAGGCGTTGATTCCCTTGGCGCGGCAAGACGA	33	AL
Spc145	GGAACAGAAAGCTACCGGCGTTTGGCTGGTTCG	33	AL
Spc146	TTGACCCACTGCATCGTTTCTAATTTATAAGCG	33	AL
Spc147	GCTCTGAGTTGCCAGCGAATCCGCCATGACCTTG	34	AL
Spc148	TAGACCCGCCCGCGCTTTCTCAGCCTGCATATA	33	AL
Spc149	TTGGGTGTCATGCTTCTATCTGAGCTGGTGTGG	33	AL
Spc150	ATCAGGTTTCATTTTTTTTCTCTCTCCCTTGA	32	AL
Spc151	GTCATTTCATGTGTCATTTTTGTATCCTCTTG	32	AL
Spc152	ATTTCTTCGGTGGCATATCAGAATTTGAGCTTA	33	AL
Spc153	CGCGTCCTCCTCATCCACTATCCCCGCACCGTA	33	AL
Spc154	GCAGGCTAACGATATGCAATCAGATAGTTGG	31	AL
Spc155	AAGGCCGTCAGTCTGAGAGATTCGTTCATGTGA	33	AL
Spc156	AGACTGGGGTGAGTTCGACAAGCTATGTATGA	32	AL
Spc157	AGATGGCTGGGGTGTCATGCTGCCATCCTCGC	32	AL
Spc158	GGATACAATGGACGATGGACCGCTGGAAAGGT	32	AL
Spc159	AGAGAGAGGAACGATCTCCTCGACCTATCCCG	32	AL
Spc160	AGCAGCAGCGTAGAAAAGCAGCTGCGCATTTTC	33	AL
Spc161	TGTGCCCGGGGCTTTTTTCCGGGGGTGGGCTTA	33	AL
Spc162	ATGCATCAAGACGTTATCACATCGCTATTTAG	32	AL
Spc163	GTAGAGAGAG	10	AL
Spc164	TTCAAGCGCAGCGCATCCGTCCCGGTCACATAT	33	AL
Spc165	GGAACGGCAGCGGTCAATATCGTTAAGGGAGCA	33	AL
Spc166	ACGTGCTATACTGCGCCTGGGCCTGCTG	28	AL
Spc167	TCCGCGAAGAGGTGGCCAAAGACTACCT	28	AL
Spc168	ACCAGCTTCCCTTTAATCGATACATCCAGAGGCACTAGTT TTTGGCTA	48	AL

Spc169	GACAGCTTCCCATCAATCGATACTTCCAGTGGGGAAAAT TTTGGTTA	47	AL
Spc170	TGCTACCGGGCCGGAATCGACAGAAAAGGCAT	32	EF
Spc171	CATGAGCTCGTCCCGATGCAAAAGCCTCTCCT	32	EF
Spc172	TCTCCATCAGCCACCCTACCCGAATCGCCGCAC	33	EF
Spc173	TGCTGCGTGGCAGGAGTATAGCCGCGGGGTAAG	33	EF
Spc174	CTACGGGCCCAGAGTCAGGCCGATGTGGAGGGT	33	EF, TB
Spc175	TTGCTGGAAGATAAACCAGAGATAGCCGGTCA	32	TB
Spc176	GTCAGCGCGGGTCCGTCGTAAACTGATGAAGC	32	TB
Spc177	GGAATGGGGCTGTGGCAAGCTATGGTACTCT	31	TB
Spc178	ATAACTGCACATCACTAACCAGCTTCCCCTT	31	TB
Spc179	ACCCTCCACATCGGCCTGACTCTGGGCCCGTAG	33	EF
Spc180	CCTGACGCAGCATGGATCGGGCGAGTCTTTACTA	34	EF
Spc181	TCCAGCGCGCCAGTCAGCCCCCCGCCCGTCTCG	33	EF
Spc182	TGAGAAATTTGATCCCGATTTTCCGGCTTTTTG	33	EF
Spc183	CGTGCGGCGATTCGGGTAGGGTGGCTGATGGAGA	34	EF
Spc184	CAGGAGAGGCTTTTGCATCGGGACGAGCTCATG	33	EF
Spc185	CATGCCTTTTCTGTCGATTCCGGCCCGGTAGCA	33	EF
Spc186	TGCAAAAGAGCAGGCCGAGACAGGCAAGATAA	32	EF
Spc187	AGAAGTTCAAGTATATGGAAGAGGAGGGACTTG	33	EF
Spc188	TCGGAATCACCCCCCACATCGATACGGAAAGTA	33	EF
Spc189	ATTCGACAAGTGCCGAGAAAATGAGGGCTGTCG	33	EF
Spc190	CCGTGGCGGGCACACCATCGTGTACGAGGCCTA	33	EF
Spc191	TCGGTCGTGTCCGACTTCATCAACTCCGTCGAG	33	EF
Spc192	CTTACCCCGCGGCTATACTCCTGCCACGCAGCA	33	EF
Spc193	TCGTTCGGGTGGCACACTGATAGCGCTTTGTCG	33	EF
Spc194	ATGGCTTTAAGCGTCGACGAGAGCGATGTGGAG	33	EF
Spc195	TTGGACCCAAGCTCGTCCATCGTCGTCTTGCCG	33	EF
Spc196	TCGGCTTCAAGCGCGGAGGTGTCGGGCTCCGTA	33	EF
Spc197	ACGACGAGGTTGATAGCGTCAAATATGCCGATG	33	EF
Spc198	TTGACGATGGCCCACTCCGCCCTGAGCTCTCTA	33	EF
Spc199	CATAGTGCGTCCTGATCATCGCTCCGAAGCTGT	33	EF
Spc200	TCCCAGTGGCGAAGTCGAAGACGGCCGCCAGAACG	35	EF
Spc201	CCTGCCGTCATTTCTTGTCCCATAAATCTCCCG	33	EF
Spc202	TTTTGAGATGGAGGCTCAGTAGCTTGATCTCCA	33	EF
Spc203	GCATCGGTCTTGCCGACGACGACATTGATCCCA	33	EF
Spc204	CGAGACTGAGCAGCTCCGTCGCGAGTCCGATAA	33	EF
Spc205	CGGAAGGCTATCGCCGGCCATATCCACGCCTGG	33	EF
Spc206	ATGGCTTTAAGCGTCGACGAGAGCGACGTGGTG	33	EF
Spc207	CCACTTCCGAATGGCCCTGATAATCTTCTTATTG	34	EF
Spc208	CGACACCAACGGGCAGGGTGCCCTACAGTCAGG	33	EF
Spc209	CTAAATCGGCAAGATTGCTCGTTCTCCGTGCCA	33	EF
Spc210	TCGACAGGGTTCCACCTTGGG	21	EF
Spc211	CTCCGGCAAGGCTTTCTTTGACAGAGTCGGAGA	33	EF
Spc212	ATCTTCTCGTCAAGCCGGTTGATCGCTGTCACA	33	EF

Spc213	ACGACGGTGCGCAGCACCGAGATTTGCTGCCGG	33	EF
Spc214	GTACTGCTTGCAAAGCGGCGTCCTGACCTTTGA	33	EF
Spc215	AGTCAGGCCGATGTGGAGGGTTATGAGCAGCA	32	EF
Spc216	CGGGACCAGAAACGTACTTGACGACCACGCCTTA	34	EF
Spc217	CCCTGAAAACTCCCTACCGTCGCACCGAAATCG	33	EF
Spc218	CCCCATGGCTCACCCCGATCTTCAACGCCGCCG	33	EF
Spc219	ATCACAAACCTTGTCGAAAAGCCCCGTGAATGG	33	EF
Spc220	CAGGAAGTTTATCGTCGTTCGTCACGAAGCCAG	33	EF
Spc221	GGATAGCTGCGCTACTTCTTGTCGCCCTCACGA	33	EF
Spc222	CTCCGCCGCAACGAGGCAGCAATCGCGGCAGTG	33	EF
Spc223	CGCCTGGTGTATGTGCCGCTGACGCAGGGGCAG	33	EF
Spc224	GCTTTGCGCCTGCTCCGCAACGCCTCCGTAGCAG	34	EF
Spc225	GGAGTGTCATTGTAAAAAATTGACGCGTATACA	33	EF
Spc226	GTATGGTGTCGGGTGAGGATGATCTGGCTTTTG	33	EF
Spc227	GCCCCGGAAAGCGAGTTCCATGCTCCCCTGAAG	33	EF
Spc228	GGTTGGCCAACTCTCGCCGGTCGGACGCGTTCGG	34	EF
Spc229	TGACGCGTCGGCGGGGTCGCTATGTCGCCCGTGG	34	EF
Spc230	TCTGCACGGTATACAATCCCCCGCCCCCGGTCA	33	EF
Spc231	ACGAGTTTCGGGCCATTTAGGGCGGGGGTGAG	32	EF
Spc232	CCCAGCGGGTGGTTGTCTTGGCGGCGTGCAGC	32	EF
Spc233	CTGGCGGATCTCAGAGCGTGGCGGCTCGGGTGC	33	TB
Spc234	CGCTGTGTCGATAACGACCGCAATCTCATCTAG	33	TB
Spc235	TGCCTGTTGAATAATCGTAAACGCGTTAAATGA	33	TB
Spc236	TTCGATACCGCGATTGTTTTGAGTGGTGTTCAG	33	TB
Spc237	TAAGCCCTCGCTTAGTAGGTATTCTTTCCCGTCA	34	TB
Spc238	TGTAGTTACCATTAGCCTGTCTATTTTTACATA	33	TB
Spc239	CTATTTTCTATCTTTTTTCTTCGTGCCCCAGCC	33	TB
Spc240	CTTATCTTCTGGAAAAGAAAAAGATGTCGTAGA	33	TB
Spc241	CAAAGCCGAGTTCTACCGCCGCGCCGAGGAGAAG	34	TB
Spc242	TGGTATGGAGAATTGACATCCTTTGATCCAAAA	33	TB
Spc243	TTGATATTGAGAAGTTGAAAGGGGAGGTTGATC	33	TB
Spc244	CATACGACGCGCACAATACATTTTAGCGACGAG	33	TB
Spc245	CAATATCTTGGTCAAAGGGACCAGCAAGATCTCA	34	TB
Spc246	TGGGAAGCTGGTTAGTGATGAGCAGCCACGCCA	33	TB
Spc247	CAAGAGCCCGACGACCGTTTTTGCTCTTATTGT	33	TB
Spc248	TGAAGATAGTGAGGTACGCCAGTAGCACGGTTG	33	TB
Spc249	TTCTAGGTTGATACGATGGCAGAGAAGATCCACC	34	TB
Spc250	TTACGGCTTCACAAGTAACGCATCTGTCACCACA	34	TB
Spc251	CTACGACATTGACGATTTCACCATTGGCTATAT	33	TB
Spc252	CAAGCTTCCCATTAATCGATACTTCCAGCGGGA	33	TB
Spc253	CTAAACGAGGAGCACAGCATGAATCATGAACAG	33	TB
Spc254	TGGCACGGAGAACGAGCAATCTTGCCGATTTAG	33	TB
Spc255	CCTGACTGTAGGGCACCCTGCCCGTTGGTGTCG	33	TB
Spc256	CAATAAGAAGATTATCAGGGCCATTCGGAAGTGG	34	TB

Spc257	ACAGCTTCGGAGCGATGATCAGGACGCACTATG	33	TB
Spc258	CTACGGGCCCAGAGTCAGGCCGATGTGGAGGGT	33	TB
CRISPR repeats			
Rpt1	GAAACACCCCCACGAGCGTGGGGAAGAC	28	AL, EF
Rpt2	AAAACACCCCCACGAGCGTGGGGAAGAC	28	AL
Rpt3	GTCTTCCCCACGCTCGTGGGGGTGTTTC	28	AL, EF, TB
Rpt4	GTCTTCCCCACGCTCGTGGGGGTGTTCA	28	AL
Rpt5	GTCTTCCCCACGCTCGTGGGGGTGTTTCT	29	AL
Rpt6	GGTCTTCCCCACGCTCGTGGGGGTGTTTCCA	31	AL
Rpt7	GAGCGTGGGGAAGACGA	17	AL
Rpt8	GAAACACCCCCACGAGCGTGGGGAAGACGC	30	AL
Rpt9	GAAACACCCCCACGAGCGTGGGGAAGACAC	30	AL
Rpt10	GCGTGGGGAAGAC	13	AL
Rpt11	AGAAACACCCCCACGAGCGTGGGGAAGAC	29	AL
Rpt12	GGAAACACCCCCACGAGCGTGGGGAAGAC	29	AL
Rpt13	GTCTTCCCCACGCTCGTGGGGGTGTTTCG	29	AL
Rpt14	GTCTTCCCCACGCTCGTGGGGGTGTTTCC	29	AL, EF
Rpt15	GTCTTCCTGCACTTAT	16	AL
Rpt16	CTCGTGGGGGTGTTTCCCT	19	AL
Rpt17	CCGGTCTTCCCCACGCTCGTGGGGGTGTTTCCCT	34	AL
Rpt18	CCGGTCTTCCCCACGCTCGTGGGGGTGTTTCCAT	34	AL
Rpt19	CGCGTGGTATGGCTGCTCATCACTA	25	AL
Rpt20	CGCGTGGTATGGCTGCTCATCGCTA	25	AL
Rpt21	CGCGTGGTTTAGCTGCTCATCGCTA	25	AL
Rpt22	AGTCTTCCCCACGCTCGTGGGGGTGTTTC	29	EF
Rpt23	GGTCTTCCCCACGCTCGTGGGGGTGTTTC	29	EF
Rpt24	AGGAAACACCCCCACGAGCGTGGGGAAGAC	30	TB
Rpt25	GGGAAACACCCCCACGAGCGTGGGGAAGAC	30	TB
Rpt26	AAGAAACACCCCCACGAGCGTGGGGAAGAC	30	TB
Rpt27	GAAACACCCCCACGAGCGTGGGGAA	25	EF
Rpt28	GTCTTCCCCACGCTCGAGGGGGTGTTTCC	29	EF

[A] spacer (Spc); repeat (Rpt).

Table S11 Viral contigs with matches to *Ca*. Chlorobium antarcticum spacers.

Viral contig ID[A]	IMG Genome ID[B]	Viral contig origin[C]	Viral cluster or singleton[D]	Spacer number[E]	Identity (%)[F]
Matches to AL *Ca.* Chlorobium antarcticum spacers					
Ga0302067_10021	3300028204	AL	cl_248	Spc61	100
Ga0302067_10021	3300028204	AL	cl_248	Spc139	100
Ga0302067_10021	3300028204	AL	cl_248	Spc154	100
Ga0302067_10021	3300028204	AL	cl_248	Spc167	100
Ga0307251_100475	3300028442	DL	cl_268	Spc76	100
Ga0306891_100555	3300028406	DL	cl_268	Spc76	100
Ga0307251_100475	3300028442	DL	cl_268	Spc124	100
Ga0306891_100555	3300028406	DL	cl_268	Spc124	100
Ga0307944_1003105	3300031396	OL	cl_694	Spc18	100
Ga0307932_1004301	3300031339	OL	cl_694	Spc18	100
Ga0307983_1002568	3300031269	OL	cl_694	Spc18	100
Ga0307958_1003547	3300031214	OL	cl_694	Spc18	100
Ga0302078_100030	3300028218	OL	cl_694	Spc18	100
Ga0302084_100296	3300028217	OL	cl_694	Spc18	100
Ga0302075_100225	3300028206	OL	cl_694	Spc18	100
Ga0307946_1005066	3300031684	OL	cl_694	Spc18	100
Ga0307978_1009036	3300031613	OL	cl_694	Spc18	100
Ga0307966_1015689	3300031607	OL	cl_694	Spc18	100
Ga0307945_1012266	3300031404	OL	cl_694	Spc18	100
Ga0307933_1005271	3300031403	OL	cl_694	Spc18	100
Ga0307963_1014151	3300031394	OL	cl_694	Spc18	100
Ga0307934_1000391	3300031329	OL	cl_694	Spc18	100
Ga0307942_1013718	3300031225	OL	cl_694	Spc18	100
Ga0307982_1015196	3300031224	OL	cl_694	Spc18	100
Ga0307948_1012832	3300031221	OL	cl_694	Spc18	100
Ga0307974_1013151	3300031211	OL	cl_694	Spc18	100
Ga0307944_1003105	3300031396	OL	cl_694	Spc97	97
Ga0307932_1004301	3300031339	OL	cl_694	Spc97	97
Ga0307983_1002568	3300031269	OL	cl_694	Spc97	97
Ga0307958_1003547	3300031214	OL	cl_694	Spc97	97
Ga0302078_100030	3300028218	OL	cl_694	Spc97	97
Ga0302084_100296	3300028217	OL	cl_694	Spc97	97
Ga0302075_100225	3300028206	OL	cl_694	Spc97	97
Ga0307946_1005066	3300031684	OL	cl_694	Spc97	97
Ga0307978_1009036	3300031613	OL	cl_694	Spc97	97
Ga0307966_1015689	3300031607	OL	cl_694	Spc97	97
Ga0307945_1012266	3300031404	OL	cl_694	Spc97	97
Ga0307933_1005271	3300031403	OL	cl_694	Spc97	97
Ga0307963_1014151	3300031394	OL	cl_694	Spc97	97
Ga0307951_1010625	3300031335	OL	cl_694	Spc97	97
Ga0307934_1000391	3300031329	OL	cl_694	Spc97	97
Ga0307942_1013718	3300031225	OL	cl_694	Spc97	97
Ga0307982_1015196	3300031224	OL	cl_694	Spc97	97
Ga0307948_1012832	3300031221	OL	cl_694	Spc97	97
Ga0307974_1013151	3300031211	OL	cl_694	Spc97	97
Ga0222695_1002624	3300023253	AL	cl_1024	Spc11	100
Ga0222696_1002166	3300023233	AL	cl_1024	Spc11	100
Ga0222690_1000793	3300023227	AL	cl_1024	Spc11	100

Ga0222665_1003383	3300022864	AL	cl_1024	Spc11	100
Ga0208904_1006197	3300025669	AL	cl_1024	Spc11	100
Ga0222689_1000957	3300023231	AL	cl_1024	Spc11	97
Ga0222689_1000957	3300023231	AL	cl_1024	Spc33	100
Ga0208904_1006197	3300025669	AL	cl_1024	Spc33	100
Ga0302060_10025	3300028201	AL	cl_1024	Spc35	100
Ga0302060_10025	3300028201	AL	cl_1024	Spc40	100
Ga0222689_1000957	3300023231	AL	cl_1024	Spc52	100
Ga0208904_1006197	3300025669	AL	cl_1024	Spc52	100
Ga0302061_10032	3300028203	AL	cl_1024	Spc59	100
Ga0222638_1002074	3300023298	AL	cl_1024	Spc59	100
Ga0222695_1002624	3300023253	AL	cl_1024	Spc59	100
Ga0222696_1002166	3300023233	AL	cl_1024	Spc59	100
Ga0222689_1000957	3300023231	AL	cl_1024	Spc59	100
Ga0222690_1000793	3300023227	AL	cl_1024	Spc59	100
Ga0222665_1003383	3300022864	AL	cl_1024	Spc59	100
Ga0222699_1002408	3300022846	AL	cl_1024	Spc59	100
Ga0222656_1001806	3300022834	AL	cl_1024	Spc59	100
Ga0208904_1006197	3300025669	AL	cl_1024	Spc59	100
Ga0208905_1004525	3300025661	AL	cl_1024	Spc59	97
Ga0302067_10039	3300028204	AL	cl_1024	Spc71	100
Ga0302061_10032	3300028203	AL	cl_1024	Spc71	100
Ga0302060_10025	3300028201	AL	cl_1024	Spc71	100
Ga0222638_1002074	3300023298	AL	cl_1024	Spc71	100
Ga0222695_1002624	3300023253	AL	cl_1024	Spc71	100
Ga0222696_1002166	3300023233	AL	cl_1024	Spc71	100
Ga0222689_1000957	3300023231	AL	cl_1024	Spc71	100
Ga0222690_1000793	3300023227	AL	cl_1024	Spc71	100
Ga0222665_1003383	3300022864	AL	cl_1024	Spc71	100
Ga0222699_1002408	3300022846	AL	cl_1024	Spc71	100
Ga0222656_1001806	3300022834	AL	cl_1024	Spc71	100
Ga0208904_1006197	3300025669	AL	cl_1024	Spc71	100
Ga0208905_1004525	3300025661	AL	cl_1024	Spc71	100
Ga0208900_1004295	3300025433	AL	cl_1024	Spc71	100
Ga0302067_10039	3300028204	AL	cl_1024	Spc72	100
Ga0302060_10025	3300028201	AL	cl_1024	Spc72	100
Ga0222638_1002074	3300023298	AL	cl_1024	Spc72	100
Ga0222695_1002624	3300023253	AL	cl_1024	Spc72	100
Ga0222696_1002166	3300023233	AL	cl_1024	Spc72	100
Ga0222689_1000957	3300023231	AL	cl_1024	Spc72	100
Ga0222690_1000793	3300023227	AL	cl_1024	Spc72	100
Ga0222665_1003383	3300022864	AL	cl_1024	Spc72	100
Ga0222699_1002408	3300022846	AL	cl_1024	Spc72	100
Ga0222656_1001806	3300022834	AL	cl_1024	Spc72	100
Ga0208904_1006197	3300025669	AL	cl_1024	Spc72	100
Ga0208905_1004525	3300025661	AL	cl_1024	Spc72	100
Ga0208900_1004295	3300025433	AL	cl_1024	Spc72	100
Ga0302061_10032	3300028203	AL	cl_1024	Spc72	100
Ga0302067_10039	3300028204	AL	cl_1024	Spc128	100
Ga0302060_10025	3300028201	AL	cl_1024	Spc128	100
Ga0222638_1002074	3300023298	AL	cl_1024	Spc128	100
Ga0222695_1002624	3300023253	AL	cl_1024	Spc128	100
Ga0222696_1002166	3300023233	AL	cl_1024	Spc128	100

Ga0222689_1000957	3300023231	AL	cl_1024	Spc128	100
Ga0222690_1000793	3300023227	AL	cl_1024	Spc128	100
Ga0222665_1003383	3300022864	AL	cl_1024	Spc128	100
Ga0222699_1002408	3300022846	AL	cl_1024	Spc128	100
Ga0222656_1001806	3300022834	AL	cl_1024	Spc128	100
Ga0208904_1006197	3300025669	AL	cl_1024	Spc128	100
Ga0208905_1004525	3300025661	AL	cl_1024	Spc128	100
Ga0208900_1004295	3300025433	AL	cl_1024	Spc128	100
Ga0302061_10032	3300028203	AL	cl_1024	Spc128	100
Ga0302067_10039	3300028204	AL	cl_1024	Spc129	100
Ga0302061_10032	3300028203	AL	cl_1024	Spc129	100
Ga0302060_10025	3300028201	AL	cl_1024	Spc129	100
Ga0222638_1002074	3300023298	AL	cl_1024	Spc129	100
Ga0222695_1002624	3300023253	AL	cl_1024	Spc129	100
Ga0222696_1002166	3300023233	AL	cl_1024	Spc129	100
Ga0222689_1000957	3300023231	AL	cl_1024	Spc129	100
Ga0222690_1000793	3300023227	AL	cl_1024	Spc129	100
Ga0222665_1003383	3300022864	AL	cl_1024	Spc129	100
Ga0222699_1002408	3300022846	AL	cl_1024	Spc129	100
Ga0222656_1001806	3300022834	AL	cl_1024	Spc129	100
Ga0208904_1006197	3300025669	AL	cl_1024	Spc129	100
Ga0208905_1004525	3300025661	AL	cl_1024	Spc129	100
Ga0208900_1004295	3300025433	AL	cl_1024	Spc129	100
Ga0302061_10032	3300028203	AL	cl_1024	Spc141	100
Ga0222638_1002074	3300023298	AL	cl_1024	Spc141	100
Ga0222695_1002624	3300023253	AL	cl_1024	Spc141	100
Ga0222696_1002166	3300023233	AL	cl_1024	Spc141	100
Ga0222689_1000957	3300023231	AL	cl_1024	Spc141	100
Ga0222690_1000793	3300023227	AL	cl_1024	Spc141	100
Ga0222665_1003383	3300022864	AL	cl_1024	Spc141	100
Ga0222699_1002408	3300022846	AL	cl_1024	Spc141	100
Ga0222656_1001806	3300022834	AL	cl_1024	Spc141	100
Ga0208904_1006197	3300025669	AL	cl_1024	Spc141	100
Ga0208905_1004525	3300025661	AL	cl_1024	Spc141	97
Ga0302061_10032	3300028203	AL	cl_1024	Spc152	100
Ga0222638_1002074	3300023298	AL	cl_1024	Spc152	100
Ga0222695_1002624	3300023253	AL	cl_1024	Spc152	100
Ga0222696_1002166	3300023233	AL	cl_1024	Spc152	100
Ga0222689_1000957	3300023231	AL	cl_1024	Spc152	100
Ga0222690_1000793	3300023227	AL	cl_1024	Spc152	100
Ga0222665_1003383	3300022864	AL	cl_1024	Spc152	100
Ga0222699_1002408	3300022846	AL	cl_1024	Spc152	100
Ga0222656_1001806	3300022834	AL	cl_1024	Spc152	100
Ga0208904_1006197	3300025669	AL	cl_1024	Spc152	100
Ga0208905_1004525	3300025661	AL	cl_1024	Spc152	97
Ga0302088_100566	3300028225	OL	cl_4221	Spc18	100
Ga0302088_100566	3300028225	OL	cl_4221	Spc44	100
Ga0302088_100566	3300028225	OL	cl_4221	Spc97	100
Ga0307255_100262	3300028427	DL	cl_9027	Spc34	100
Ga0306904_101322	3300028358	DL	cl_9027	Spc34	100
Ga0307255_100262	3300028427	DL	cl_9027	Spc41	100
Ga0306904_101322	3300028358	DL	cl_9027	Spc41	100
Ga0307255_100262	3300028427	DL	cl_9027	Spc78	100

Ga0307255_100262	3300028427	DL	cl_9027	Spc122	100
Ga0306906_1001873	3300028374	RL13	cl_9176	Spc19	100
Ga0306906_1001873	3300028374	RL13	cl_9176	Spc19	97
Ga0306869_1003639	3300028377	RL6	cl_9221	Spc18	100
Ga0306912_1000992	3300028376	RL11	cl_9221	Spc18	100
Ga0306912_1000992	3300028376	RL11	cl_9221	Spc78	100
Ga0306912_1000992	3300028376	RL11	cl_9221	Spc122	100
Ga0136601_1000194	3300023049	RL2	cl_9451	Spc21	100
Ga0136601_1000194	3300023049	RL2	cl_9451	Spc44	100
Ga0306907_1002194	3300028366	RL13	sg_1234	Spc21	100
Ga0306907_1002194	3300028366	RL13	sg_1234	Spc34	100
Ga0306907_1002194	3300028366	RL13	sg_1234	Spc41	100
Ga0306907_1002194	3300028366	RL13	sg_1234	Spc78	100
Ga0306907_1002194	3300028366	RL13	sg_1234	Spc122	100
Ga0306907_1002194	3300028366	RL13	sg_1234	Spc164	97
Ga0306906_1001422	3300028374	RL13	sg_1352	Spc34	100
Ga0306906_1001422	3300028374	RL13	sg_1352	Spc41	100
Ga0306906_1001422	3300028374	RL13	sg_1352	Spc78	100
Ga0306906_1001422	3300028374	RL13	sg_1352	Spc97	100
Ga0306906_1001422	3300028374	RL13	sg_1352	Spc122	100
Ga0306906_1000248	3300028374	RL13	sg_1370	Spc34	100
Ga0306906_1000248	3300028374	RL13	sg_1370	Spc41	100
Ga0306869_1000373	3300028377	RL6	sg_1439	Spc78	100
Ga0306869_1000373	3300028377	RL6	sg_1439	Spc122	100
Ga0302060_10058	3300028201	AL	sg_14537	Spc61	100
Ga0302060_10058	3300028201	AL	sg_14537	Spc139	100
Ga0302060_10058	3300028201	AL	sg_14537	Spc154	100
Ga0302060_10058	3300028201	AL	sg_14537	Spc167	100
Ga0302067_10044	3300028204	AL	sg_14551	Spc60	100
Ga0302067_10044	3300028204	AL	sg_14551	Spc64	100
Ga0302067_10044	3300028204	AL	sg_14551	Spc136	100
Ga0302067_10044	3300028204	AL	sg_14551	Spc140	100
Ga0302067_10044	3300028204	AL	sg_14551	Spc153	100
Ga0302067_10044	3300028204	AL	sg_14551	Spc157	100
Ga0302067_10019	3300028204	AL	sg_14554	Spc14	100
Ga0302067_10019	3300028204	AL	sg_14554	Spc21	100
Ga0302067_10019	3300028204	AL	sg_14554	Spc32	100
Ga0302067_10019	3300028204	AL	sg_14554	Spc49	100
Ga0302067_10019	3300028204	AL	sg_14554	Spc51	100
Ga0302067_10019	3300028204	AL	sg_14554	Spc61	97
Ga0302067_10019	3300028204	AL	sg_14554	Spc62	100
Ga0302067_10019	3300028204	AL	sg_14554	Spc138	100
Ga0302067_10019	3300028204	AL	sg_14554	Spc139	97
Ga0302067_10019	3300028204	AL	sg_14554	Spc144	100
Ga0302067_10019	3300028204	AL	sg_14554	Spc155	100
Ga0302067_10019	3300028204	AL	sg_14554	Spc164	100
Ga0302067_10019	3300028204	AL	sg_14554	Spc167	100
Ga0302067_10019	3300028204	AL	sg_14554	Spc168	98
Ga0302075_100027	3300028206	OL	sg_14571	Spc76	97
Ga0302075_100027	3300028206	OL	sg_14571	Spc124	97
Ga0302068_100085	3300028219	AL	sg_14796	Spc60	100
Ga0302068_100085	3300028219	AL	sg_14796	Spc96	100
Ga0302068_100085	3300028219	AL	sg_14796	Spc140	100

Ga0302068_100085	3300028219	AL	sg_14796	Spc153	100
Ga0302064_100390	3300028221	AL	sg_14826	Spc21	100
Ga0302064_100390	3300028221	AL	sg_14826	Spc49	100
Ga0302064_100390	3300028221	AL	sg_14826	Spc51	100
Ga0302064_100390	3300028221	AL	sg_14826	Spc56	100
Ga0302064_100390	3300028221	AL	sg_14826	Spc164	100
Ga0302055_100068	3300028226	AL	sg_14916	Spc21	100
Ga0302055_100068	3300028226	AL	sg_14916	Spc51	100
Ga0302055_100068	3300028226	AL	sg_14916	Spc56	100
Ga0302055_100068	3300028226	AL	sg_14916	Spc164	100
Ga0306909_100614	3300028405	RL2	sg_1495	Spc96	100
Ga0302071_100505	3300028228	AL	sg_14959	Spc32	97
Ga0302071_100505	3300028228	AL	sg_14959	Spc60	100
Ga0302071_100505	3300028228	AL	sg_14959	Spc96	97
Ga0302071_100505	3300028228	AL	sg_14959	Spc140	100
Ga0302071_100505	3300028228	AL	sg_14959	Spc144	97
Ga0302071_100505	3300028228	AL	sg_14959	Spc153	100
Ga0302071_100505	3300028228	AL	sg_14959	Spc11	100
Ga0306866_1000581	3300028410	RL5	sg_1541	Spc17	100
Ga0306866_1000581	3300028410	RL5	sg_1541	Spc18	100
Ga0306866_1000581	3300028410	RL5	sg_1541	Spc21	100
Ga0306866_1000581	3300028410	RL5	sg_1541	Spc78	100
Ga0306866_1000581	3300028410	RL5	sg_1541	Spc122	100
Ga0306866_1000581	3300028410	RL5	sg_1541	Spc164	97
Ga0306910_1001842	3300028412	RL2	sg_1563	Spc51	100
Ga0306910_1001842	3300028412	RL2	sg_1563	Spc164	97
Ga0307254_100878	3300028435	DL	sg_1648	Spc17	100
Ga0307254_100926	3300028435	DL	sg_1650	Spc76	100
Ga0307254_100926	3300028435	DL	sg_1650	Spc124	100
Ga0307254_100801	3300028435	DL	sg_1655	Spc34	100
Ga0307254_100801	3300028435	DL	sg_1655	Spc41	100
Ga0307253_100977	3300028451	DL	sg_1676	Spc76	100
Ga0307253_100977	3300028451	DL	sg_1676	Spc124	100
Ga0307253_100979	3300028451	DL	sg_1677	Spc17	100
Ga0302058_100066	3300028302	AL	sg_16816	Spc21	100
Ga0302058_100066	3300028302	AL	sg_16816	Spc49	100
Ga0302058_100066	3300028302	AL	sg_16816	Spc51	100
Ga0302058_100066	3300028302	AL	sg_16816	Spc78	100
Ga0302058_100066	3300028302	AL	sg_16816	Spc122	100
Ga0302058_100066	3300028302	AL	sg_16816	Spc164	100
Ga0307253_101054	3300028451	DL	sg_1682	Spc34	100
Ga0307253_101054	3300028451	DL	sg_1682	Spc41	100
Matches to EF *Ca.* Chlorobium antarcticum spacers					
Ga0222641_1000777	3300022828	AL	cl_248	Spc230	97
Ga0222679_1001284	3300022858	AL	cl_248	Spc230	97
Ga0222698_1002050	3300022860	AL	cl_248	Spc230	97
Ga0222701_1001644	3300022884	AL	cl_248	Spc230	97
Ga0222689_1000893	3300023231	AL	cl_248	Spc230	97
Ga0208904_1004243	3300025669	AL	cl_248	Spc230	97
Ga0302061_10026	3300028203	AL	cl_248	Spc230	97
Ga0302067_10021	3300028204	AL	cl_248	Spc230	97
Matches to TB *Ca.* Chlorobium antarcticum spacers					

Ga0302061_10032	3300028203	AL	cl_1024	Spc241	100
Ga0302067_10039	3300028204	AL	cl_1024	Spc244	100
Ga0302060_10025	3300028201	AL	cl_1024	Spc244	100
Ga0222695_1002624	3300023253	AL	cl_1024	Spc244	100
Ga0222665_1003383	3300022864	AL	cl_1024	Spc244	100
Ga0208904_1006197	3300025669	AL	cl_1024	Spc244	100
Ga0208900_1004295	3300025433	AL	cl_1024	Spc244	100
Ga0302061_10032	3300028203	AL	cl_1024	Spc245	100
Ga0222638_1002074	3300023298	AL	cl_1024	Spc245	100
Ga0222696_1002166	3300023233	AL	cl_1024	Spc245	100
Ga0222689_1000957	3300023231	AL	cl_1024	Spc245	100
Ga0222690_1000793	3300023227	AL	cl_1024	Spc245	100
Ga0222699_1002408	3300022846	AL	cl_1024	Spc245	100
Ga0208905_1004525	3300025661	AL	cl_1024	Spc245	100
Ga0302067_10039	3300028204	AL	cl_1024	Spc245	97
Ga0302060_10025	3300028201	AL	cl_1024	Spc245	97
Ga0222695_1002624	3300023253	AL	cl_1024	Spc245	97
Ga0222665_1003383	3300022864	AL	cl_1024	Spc245	97
Ga0208904_1006197	3300025669	AL	cl_1024	Spc245	97
Ga0208900_1004295	3300025433	AL	cl_1024	Spc245	97
Ga0302060_10025	3300028201	AL	cl_1024	Spc236	97
Ga0302061_10032	3300028203	AL	cl_1024	Spc244	97
Ga0222638_1002074	3300023298	AL	cl_1024	Spc244	97
Ga0222696_1002166	3300023233	AL	cl_1024	Spc244	97
Ga0222689_1000957	3300023231	AL	cl_1024	Spc244	97
Ga0222690_1000793	3300023227	AL	cl_1024	Spc244	97
Ga0222699_1002408	3300022846	AL	cl_1024	Spc244	97
Ga0222656_1001806	3300022834	AL	cl_1024	Spc244	97
Ga0208905_1004525	3300025661	AL	cl_1024	Spc244	97
Ga0306906_1001873	3300028374	RL13	cl_9176	Spc252	100
Ga0306906_1001873	3300028374	RL13	cl_9176	Spc252	97
Ga0222684_1001894	3300023295	AL	sg_10581	Spc244	97
Ga0306906_1000248	3300028374	RL13	sg_1370	Spc243	100
Ga0302067_10044	3300028204	AL	sg_14551	Spc238	100
Ga0302067_10044	3300028204	AL	sg_14551	Spc244	97
Ga0302067_10044	3300028204	AL	sg_14551	Spc241	97
Ga0302068_100085	3300028219	AL	sg_14796	Spc245	100
Ga0302068_100085	3300028219	AL	sg_14796	Spc241	97
Ga0302071_100505	3300028228	AL	sg_14959	Spc241	100
Ga0302071_100505	3300028228	AL	sg_14959	Spc249	100
Ga0307254_100878	3300028435	DL	sg_1648	Spc252	100
Ga0307254_100745	3300028435	DL	sg_1649	Spc251	100
Ga0307253_100979	3300028451	DL	sg_1677	Spc251	100

[A] Viral contigs potentially associated with *Ca.* Chlorobium antarcticum (Fig. 9), identified through matches to spacers (see the "Methods" section). [B] The IMG Genome IDs of metagenomes from which the viral contigs were assembled. [C] The Antarctic system from which the viral contigs originated: AL, Ace Lake; DL, Deep Lake; OL, Organic Lake; RL, Rauer Lakes. [D] Some viral clusters contained contigs that originated from multiple systems: cl_268, DL, RL3 and Club Lake; cl_9221, RL6 and 11; cl_248 and cl_1024, AL; cl_694 and cl_4221, OL; cl_9027, DL; cl_9451, RL2; cl_9176, RL13. [E] *Ca.* Chlorobium antarcticum spacer sequences are provided in Additional file 1: Table S10. [F] The identity of *Ca.* Chlorobium antarcticum spacer matches to viral contigs.

Table S12 Host analysis of viral clusters and singletons with matches to EF and TB *Ca*. Chlorobium antarcticum spacers.

Host phylum/class (number of host contigs)[B]	Viral cluster and singletons[A]									
	cl_248	cl_9176	sg_10581	sg_1370	sg_14551	sg_14796	sg_14959	sg_1648	sg_1649	sg_1677
Chlorobi (21)	CPv, CPb	CPv	CPb, CPv	CPv	CPb, CPv	CPb, CPv	CPb, CPv	CPv	CPv	CPv
Actinobacteria (1)						S				
Bacteroidetes (0)	RZ									
Firmicutes (1)		L								
Betaproteobacteria (4)				P		T	T			
Deltaproteobacteria (1)				D						
Gammaproteobacteria (103)	KB, MA, M	M, KP, VC	M, KP, AJ	MA, M, ME, KP, LM, AJ, PP, PS, VC	MA, M, ME, KP, AJ	MA, M, KP, AJ	MA, M, KP, AJ, VC		KP	M
Verrucomicrobia (1)			V	V	V					
Unclassified (8)				U						

[A] TB *Ca*. Chlorobium antarcticum spacers matched viral contig cluster cl_9176 and singletons sg_10581, sg_1370, sg_14551, sg_14796, sg_14959, sg_1648, sg_1649, sg_1677 and EF *Ca*. Chlorobium antarcticum spacers matched viral contig cluster cl_248. All spacer-viral contig matches had 100% identity, except those in red font. The abbreviations listed below the viral contig name denote the taxonomies of spacer-containing host contigs: AJ, *Alcanivorax jadensis*; CPb, *Chlorobium phaeobacteroides*; CPv, *Chlorobium phaeovibrioides*; D, *Desulfurivibrio* sp.; KP, *Klebsiella pneumoniae*; L, *Lactobacillus* sp.; LM, *Legionella massiliensis*; M, *Marinobacter* sp.; MA, *Marinobacter antarcticus*; ME, *Marinobacter* sp. ELB17; P, *Polaromonas* sp.; PP, *Pseudomonas putida*; PS, *Pseudomonas stutzeri*; RZ, *Runella zeae*; S, *Streptomyces* sp.; T, *Thauera* sp.; U, Unclassified; V, *Verrucomicrobium* sp. 3C; VC, *Vibrio cholerae*; note that *Ca*. Chlorobium antarcticum equates to CPv as this was the most closely related species in the database. [B] The number of host contigs containing spacers that had 100% identity matches to at least one of the viral contigs are shown in parentheses.

Table S13 Description of *Ca.* Chlorobium antarcticum metabolic capacity and metadata.

Species name	*Candidatus* Chlorobium antarcticum
Species etymology	ant.arc'ti.cum. L. neut. adj. *antarcticum* southern, Antarctic
Species status	sp. nov.
Genome type	Metagenome-assembled genome
Genome status	Draft
IMG Bin ID	3300023061_2
Bin completeness	99.45%
Bin contamination	0.55%
Total base pair count	1,812,610 bp
Number of contigs and genes	27 contigs; 1,797 genes
GC mol %	52.39%
Region of origin	Antarctica
Geographic location	Ace Lake
Latitude	68°28' S
Longitude	78°11' E
Habitat	Meromictic, saline lake
Sampling date	3 December 2014
Lake Depth	19 m
Lake temperature	3.3 °C
Metabolic capacity	Green-colored, with chlorosomes for light harvesting;<br>Obligate anaerobic photolithoautotroph;<br>Electrons donated from sulfide by oxidation to sulfate using sulfide:quinone oxidoreductase, dissimilatory sulfite reductase, polysulfide reductase-like complex 3;<br>Photoassimilation of simple organic compounds (acetate, propionate, pyruvate);<br>CO_2 assimilation by reverse tricarboxylic acid cycle;<br>Glycogen storage and mobilization;<br>Nitrogenase for nitrogen fixation;<br>Ammonia uptake and assimilation;<br>ABC transporter systems for uptake of peptides, phosphate, zinc, iron, molybdate, cobalt, cobalamin;<br>Cobalamin biosynthesis via anaerobic pathway, and cobinamide salvaging;<br>Subtype I-E CRISPR-Cas system;<br>Type I and type IV restriction-modification systems;<br>ParDE, RelFG and BrnTA type II, and AbiE type IV toxin-antitoxin systems;<br>No capacity for thiosulfate oxidation;<br>No capacity for assimilatory sulfate reduction.
Sequencing technology	Illumina HiSeq 2500-1TB
Assembly software used	BFC version r181 [20]; SPAdes v3.11.1 [21, 22]
Binning software used	MetaBAT v0.32.5 [23]; CheckM v1.0.11 [24]

The *Ca.* Chlorobium antarcticum data are presented as per the recommendations for describing novel *Candidatus* species [25].

Table S14 AL, EF and TB metagenomes used for FR analyses of *Ca*. Chlorobium antarcticum MAGs.

System	Sample collection time period and depth	Merged metagenome name[A]	*Chlorobium* OTU relative abundance (%)[B]			Total number of reads[C]
			3–20 µm-filter	0.8–3 µm-filter	0.1–0.8 µm-filter	
Ace Lake	Nov 2008 12.8 m	AL Nov2008_I	42	62	81	204,878,852
	Nov 2013 13.5 m	AL Nov2013_I	12	21	33	86,383,986
	Jul 2014 13.5 m	AL Jul2014_I	2	5	6	78,035,526
	Aug 2014 14.5 m	AL Aug2014_I	1	5	5	82,792,076
	Oct 2014 13 m	AL Oct2014_I	0	1	1	70,579,806
	Dec 2014 13.4 m	AL Dec2014_I	39	57	59	140,544,592
Ellis Fjord	Oct 2014 45 m	EF_45m	14	49	48	322,272,730
Taynaya Bay	Nov 2014 11 m	TB_11m	6 (0.22–20 µm-filter)			91,287,184

[A] The metagenomes from the three filter fractions from each sampling date of the oxic-anoxic interface from AL, and from the EF oxic-anoxic interface, were combined to form merged metagenomes. [B] Relative abundance of *Chlorobium* OTUs in specific metagenomes. [C] The number of reads indicates the total number of reads in the merged metagenomes.

Table S15 Marker genes of Chlorobiaceae family members used for the phylogenetic analysis of *Ca*. Chlorobium antarcticum.

Organism	16S rRNA gene		FmoA protein	
	Accession ID[A]	Length (in bp)	Accession ID[A]	Length (in aa)
Chlorobaculum limnaeum	NZ_CP017305.1	1505	WP_069808958.1	366
Chlorobaculum macestae	NR_116056.1	1395	-	-
Chlorobaculum parvum	NC_011027.1	1507	WP_012502817.1	365
Chlorobaculum tepidum	NR_044685.2	1450	WP_010933165.1	366
Chlorobaculum thiosulfatiphilum	NR_029321.1	1388	WP_139457377.1	366
Chlorobium chlorochromatii	NC_007514.1	1506	WP_011362353.1	366
Chlorobium chlorovibrioides	Y10649.1	1466	-	-
Chlorobium ferrooxidans	Y18253.1	1804	WP_006366194.1	366
Chlorobium gokarna	AJ888464.1	1287	-	-
Chlorobium limicola	NC_010803.1	1504	WP_012466619.1	366
Chlorobium luteolum	NC_007512.1	1504	WP_011358231.1	366
Chlorobium phaeobacteroides	NC_010831.1	1507	WP_012474280.1	367
Chlorobium phaeovibrioides	NC_009337.1	1506	WP_011890560.1	366
Chloroherpeton thalassium	NC_011026.1	1501	WP_012499263.1	370
Pelodictyon phaeoclathratiforme	NC_011060.1	1502	WP_012507834.1	366
Prosthecochloris aestuarii	NC_011059.1	1506	WP_012506146.1	367
Prosthecochloris indica	NR_132595.1	1393	-	-
Prosthecochloris marina	-	-	WP_110023260.1	367
Prosthecochloris vibrioformis	M62791.1	1507	WP_068866593.1	367
AL *Ca*. Chlorobium antarcticum	IMG taxon ID: 3300023061 Gene ID: Ga0222700_1000006154	1505	IMG taxon ID: 3300023061 Gene ID: Ga0222700_1000003178	366
EF *Ca*. Chlorobium antarcticum	IMG taxon ID: 3300031631 Gene ID: Ga0307987_100000446	1505	IMG taxon ID: 3300031631 Gene ID: Ga0307987_1000002178	366
TB *Ca*. Chlorobium antarcticum	IMG taxon ID: 3300039187 Gene ID: Ga0400661_000002_151875_153376	1502	IMG taxon ID: 3300039187 Gene ID: Ga0400661_000007_23035_24135	366

[A] Accession IDs of 16S rRNA genes, FmoA proteins or genomes of the listed species.

References

1. Gray MJ, Escalante-Semerena JC. The cobinamide amidohydrolase (cobyric acid-forming) CbiZ enzyme: A critical activity of the cobamide remodeling system of *Rhodobacter sphaeroides*. Mol Microbiol. 2009;74:1198–210.
2. BioCyc. https://biocyc.org/ (2011). MetaCyc Pathway: adenosylcobalamin biosynthesis accessed between Dec 2020 and Jan 2021.
3. Karp PD, Billington R, Caspi R, Fulcher CA, Latendresse M, Kothari A, et al. The BioCyc collection of microbial genomes and metabolic pathways. Brief Bioinform. 2017. DOI: 10.1093/bib/bbx085.
4. Rodionov DA, Vitreschak AG, Mironov AA, Gelfand MS. Comparative genomics of the vitamin B12 metabolism and regulation in prokaryotes. J Biol Chem. 2003;278:41148–59.
5. Woodson JD, Zayas CL, Escalante-Semerena JC. A new pathway for salvaging the coenzyme B12 precursor cobinamide in archaea requires cobinamide-phosphate synthase (CbiB) enzyme activity. J Bacteriol. 2003;185:7193–201.
6. Woodson JD, Escalante-Semerena JC. CbiZ, an amidohydrolase enzyme required for salvaging the coenzyme B12 precursor cobinamide in archaea. Proc Natl Acad Sci U S A. 2004;101:3591–6.
7. Rodionov DA, Hebbeln P, Gelfand MS, Eitinger T. Comparative and functional genomic analysis of prokaryotic nickel and cobalt uptake transporters: evidence for a novel group of ATP-binding cassette transporters. J Bacteriol. 2006;188:317–27.
8. Taga ME, Larsen NA, Howard-Jones AR, Walsh CT, Walker GC. BluB cannibalizes flavin to form the lower ligand of vitamin B12. Nature. 2007;446:449–53.
9. Gray MJ, Tavares NK, Escalante-Semerena JC. The genome of *Rhodobacter Sphaeroides* strain 2.4.1 encodes functional cobinamide salvaging systems of archaeal and bacterial origins. Mol Microbiol. 2008;70:824–36.
10. Hazra AB, Han AW, Mehta AP, Mok KC, Osadchiy V, Begley TP, et al. Anaerobic biosynthesis of the lower ligand of vitamin B12. Proc Natl Acad Sci U S A. 2015;112:10792–7.
11. Bowers RM, Kyrpides NC, Stepanauskas R, Smith MH, Doud D, Reddy TBK, et al. Minimum information about a single amplified genome (MISAG) and a metagenome-assembled genome (MIMAG) of bacteria and archaea. Nat Biotechnol. 2017;35:725–31.
12. ExPASy BLAST. https://web.expasy.org/blast/ (1993). UniProtKB/Swiss-Prot database accessed between Oct 2020 and May 2021.
13. NCBI BLAST. https://blast.ncbi.nlm.nih.gov/Blast.cgi (1994). UniProtKB and RefSeq databases accessed between Oct 2020 and May 2021.
14. Kalvari I, Nawrocki EP, Argasinska J, Olvera NQ, Finn RD, Bateman A, et al. Non-coding RNA analysis using the Rfam database. Curr Protoc Bioinformatics. 2018;62:e51.
15. Kalvari I, Nawrocki EP, Palacios NQ, Argasinska J, Lamkiewicz K, Marz M, et al. Rfam 14: expanded coverage of metagenomic, viral and microRNA families. Nucleic Acids Res. 2021;49:D192–D200.
16. Boldyreva D, Babenko VV, Kanygina AV, Lunina ON, Letarova MA, Kostryukova ES, et al. Genome sequences of a green-colored *Chlorobium phaeovibrioides* strain containing two plasmids and a closely related plasmid-free brown-colored strain. Microbiol Resour Announc. 2020;9:e01172-19.
17. Llorens–Marès T, Liu Z, Allen LZ, Rusch DB, Craig MT, Dupont CL, et al. Speciation and ecological success in dimly lit waters: horizontal gene transfer in a green sulfur bacteria bloom unveiled by metagenomic assembly. ISME J. 2017;11:201–11.

18. Eisen JA, Nelson KE, Paulsen IT, Heidelberg JF, Wu M, Dodson RJ, et al. The complete genome sequence of *Chlorobium tepidum* TLS, a photosynthetic, anaerobic, green-sulfur bacterium. Proc Natl Acad Sci U S A. 2002;99:9509–14.
19. Mansor M, Macalady JL. Draft genome sequence of lampenflora *Chlorobium limicola* strain Frasassi in a sulfidic cave system. Genome Announc. 2016;4:e00357-16.
20. Li H. BFC: correcting Illumina sequencing errors. Bioinformatics. 2015;31:2885–7.
21. Nurk S, Bankevich A, Antipov D, Gurevich AA, Korobeynikov A, Lapidus A, et al. Assembling single-cell genomes and mini-metagenomes from chimeric MDA products. J Comput Biol. 2013;20:714–37.
22. Nurk S, Meleshko D, Korobeynikov A, Pevzner PA. MetaSPAdes: a new versatile metagenomic assembler. Genome Res. 2017;27:824–34.
23. Kang DD, Li F, Kirton E, Thomas A, Egan R, An H, et al. MetaBAT 2: an adaptive binning algorithm for robust and efficient genome reconstruction from metagenome assemblies. PeerJ. 2019;7:e7359.
24. Parks DH, Imelfort M, Skennerton CT, Hugenholtz P, Tyson GW. CheckM: assessing the quality of microbial genomes recovered from isolates, single cells, and metagenomes. Genome Res. 2015;25:1043–55.
25. Chuvochina M, Rinke C, Parks DH, Rappé MS, Tyson GW, Yilmaz P, et al. The importance of designating type material for uncultured taxa. Syst Appl Microbiol. 2019;42:15–21.
